# Preference of Bacterial Rhamnosyltransferases for 6-Deoxysugars Reveals a Strategy To Deplete O-Antigens

**DOI:** 10.1021/jacs.3c03005

**Published:** 2023-07-12

**Authors:** Alexa P. Harnagel, Mia Sheshova, Meng Zheng, Maggie Zheng, Karolina Skorupinska-Tudek, Ewa Swiezewska, Tania J. Lupoli

**Affiliations:** † Department of Chemistry, New York University, New York, New York 10003, United States; ‡ Institute of Biochemistry and Biophysics, Polish Academy of Sciences, Warsaw, 02-106, Poland

## Abstract

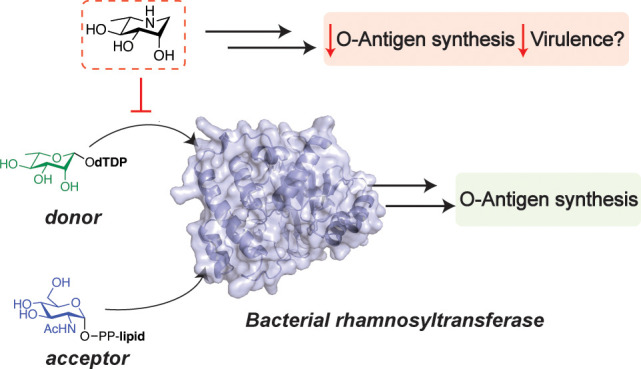

Bacteria synthesize hundreds of bacteria-specific or “rare” sugars that are absent in mammalian cells and enriched in 6-deoxy monosaccharides such as l-rhamnose (l-Rha). Across bacteria, l-Rha is incorporated into glycans by rhamnosyltransferases (RTs) that couple nucleotide sugar substrates (donors) to target biomolecules (acceptors). Since l-Rha is required for the biosynthesis of bacterial glycans involved in survival or host infection, RTs represent potential antibiotic or antivirulence targets. However, purified RTs and their unique bacterial sugar substrates have been difficult to obtain. Here, we use synthetic nucleotide rare sugar and glycolipid analogs to examine substrate recognition by three RTs that produce cell envelope components in diverse species, including a known pathogen. We find that bacterial RTs prefer pyrimidine nucleotide-linked 6-deoxysugars, not those containing a C6-hydroxyl, as donors. While glycolipid acceptors must contain a lipid, isoprenoid chain length, and stereochemistry can vary. Based on these observations, we demonstrate that a 6-deoxysugar transition state analog inhibits an RT *in vitro* and reduces levels of RT-dependent O-antigen polysaccharides in Gram-negative cells. As O-antigens are virulence factors, bacteria-specific sugar transferase inhibition represents a novel strategy to prevent bacterial infections.

Bacterial cell envelopes are rich in glycans that provide structural integrity and mediate intercellular interactions required for pathogenicity.^1−8^ These glycans are unique, as bacteria produce sugars absent in mammalian glycomes known as “rare” or “bacteria/prokaryote-specific”.^5,9−11^ Among the ∼700 rare monosaccharides, l-rhamnose (l-Rha) and other 6-deoxysugars that lack a C6-hydroxyl are enriched in bacteria compared to mammals.^12^ Across microbes, l-Rha is required for the construction of different cell envelope glycoconjugates.^9,12−15^ In the Gram-positive, Acid-fast *Mycobacterium* genus, which includes the pathogen *Mycobacterium tuberculosis* (Mtb), an α-l-Rha-(1→3)-α-d-GlcNAc linker between the peptidoglycan and arabinogalactan layers is essential for viability (Figure 1A, *left*).^16−^^20^ In many strains of the Gram-negative *Escherichia coli*, the same disaccharide is found in O-antigen (O-Ag) polysaccharides in lipopolysaccharide (LPS) (Figure 1A, *right*). O-Ags are required for virulence, and their sugar sequences distinguish serotypes.^9,13,21,22^ While the cell envelopes of *E. coli* and Mtb differ, l-Rha is incorporated by a dedicated rhamnosyltransferase (RT) called WbbL in both species.

**Figure 1:**
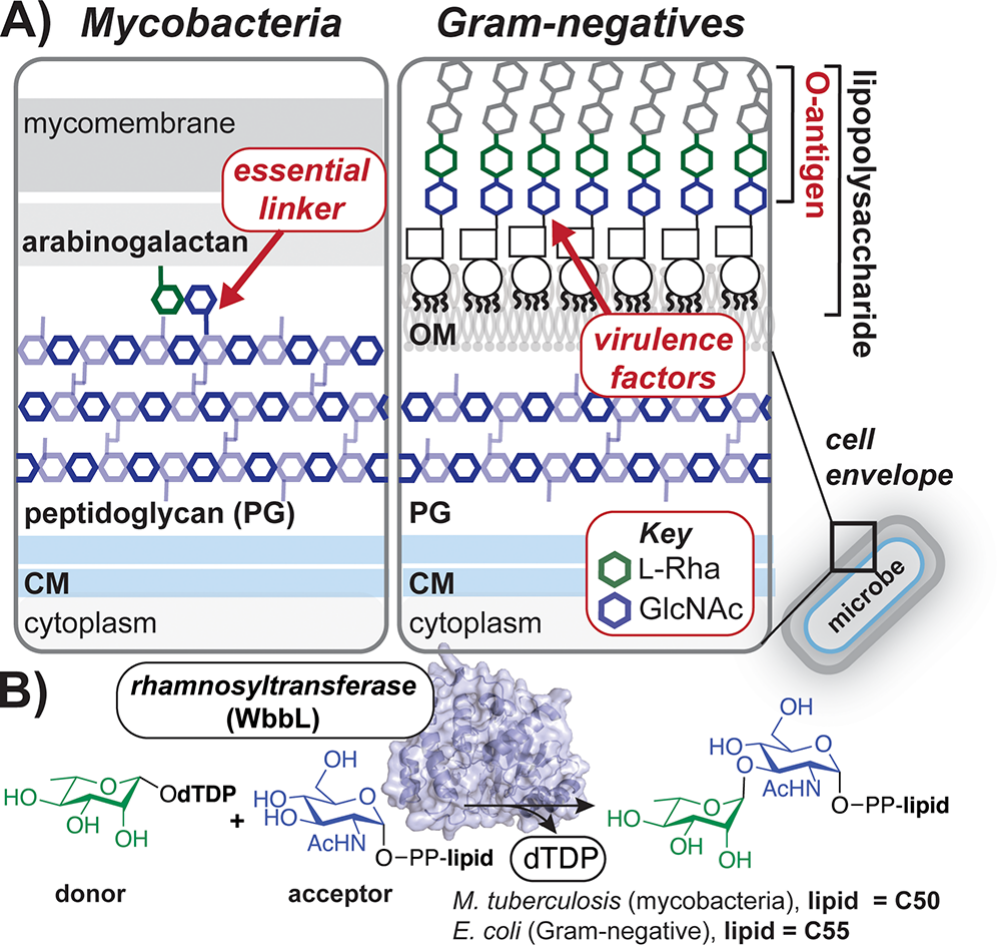
l-Rha is required for glycan assembly in diverse bacteria. (A) Schematic of mycobacterial and Gram-negative cell envelopes with the roles of l-Rha-GlcNAc linkages highlighted. (B) Rhamnosyltransferase-mediated synthesis of these linkages with a model of *E. coli* WbbL shown. (CM = cytoplasmic membrane; OM = outer membrane.)

WbbL transfers a sugar from the dTDP-β-l-Rha donor to an acceptor, lipid pyrophosphate-GlcNAc (Figure 1B).^23,24^*E. coli* utilizes the lipid carrier undecaprenyl pyrophosphate (C55-PP, Und-PP), while Mtb uses decaprenyl pyrophosphate (C50-PP). Despite the presence of *wbbL* in diverse bacterial species, little is known about the mechanisms by which glycolipid RTs select donors among the vast pool of cellular sugar metabolites.^17,25−28^ Like many O-Ag glycosyltransferases, WbbL is localized proximal to the cytoplasmic membrane, which complicates purification.^7,29,30^ Additionally, discrete donor and acceptor substrates are not readily accessible, as syntheses of activated β-l-sugars^31−38^ and glycolipids with long hydrophobic tails^39−43^ are limited.

Since WbbL is essential for virulence (*E. coli*) or survival (Mtb), obtaining a better understanding of substrate preferences would have implications in the design of both antibiotic and antivirulence agents. Resulting antivirulence strategies may avoid selective pressures that drive antibiotic resistance mechanisms.^44^ Here, we study three glycolipid RTs from different species, two from Gram-negatives, and one from mycobacteria. A collection of synthetic (deoxy)nucleoside diphosphate-sugar ((d)NDP-sugar) donor and glycolipid acceptor analogs are used to examine molecular recognition of substrates. These findings lead to a tactic to inhibit *E. coli* WbbL *in vitro* and in cells.

To evaluate donor recognition, we assessed binding of RT to dTDP-β-l-Rha analogs obtained by our synthetic and chemoenzymatic routes.^31,45^ WbbL is predicted to be membrane associated;^18,30,47^ hence, *E. coli* and Mtb WbbL membrane fractions were isolated. To broaden the scope of our study and obtain higher protein quantities, we identified a putative similar protein, RfbF, in the Gram-negative thermophile *Thermus thermophilus* using sequence-based analyses (Tables S1–S2, Figures S1–S2).^23,24,48−51^ Soluble RfbF isolation enabled isothermal titration calorimetry (ITC) experiments (Figures 2, S3–S13). dTDP-β-l-Rha bound RfbF with a stoichiometry of ∼1 and ∼8 μM affinity (Figure 2A–B, Table S3), which is in the concentration range of cellular nucleotide-sugars and the measured *K*_M_ (∼35 μM) with crude Mtb WbbL.^30^ An l-Rha-1-phosphate fragment demonstrated binding, but beyond a measurable *K*_D_ (>1 mM).^52^ Unlike reported RTs that glycosylate glycans, natural products or proteins,^47,53−56^ RfbF did not bind nucleotide alone (dTDP) at concentrations tested (Figures S3–S4). Stereochemical inversion at the C4-position using dTDP-6-deoxy-β-l-talose (dTDP-β-l-6dTal) led to comparable affinity as the native substrate, while altering the C2- and C4-positions with dTDP-β-l-fucose (dTDP-β-l-Fuc) produced an ∼15-fold enhancement in *K*_D_, indicating the C2-hydroxyl conformation is important for recognition. The consequence of C6-hydroxylation was assessed with dTDP-β-l-mannose (dTDP-β-l-Man) and the bacterial metabolite dTDP-α-Glc, which showed 60- and ∼75-fold increases in *K*_D_, respectively, compared to the native donor (Figure 2C, left). Similarly, alteration of the anomeric position in dTDP-α-l-Rha resulted in no detectable binding. Hence, changes to the anomeric or C6 position may cause steric clashes in the active site.

**Figure 2:**
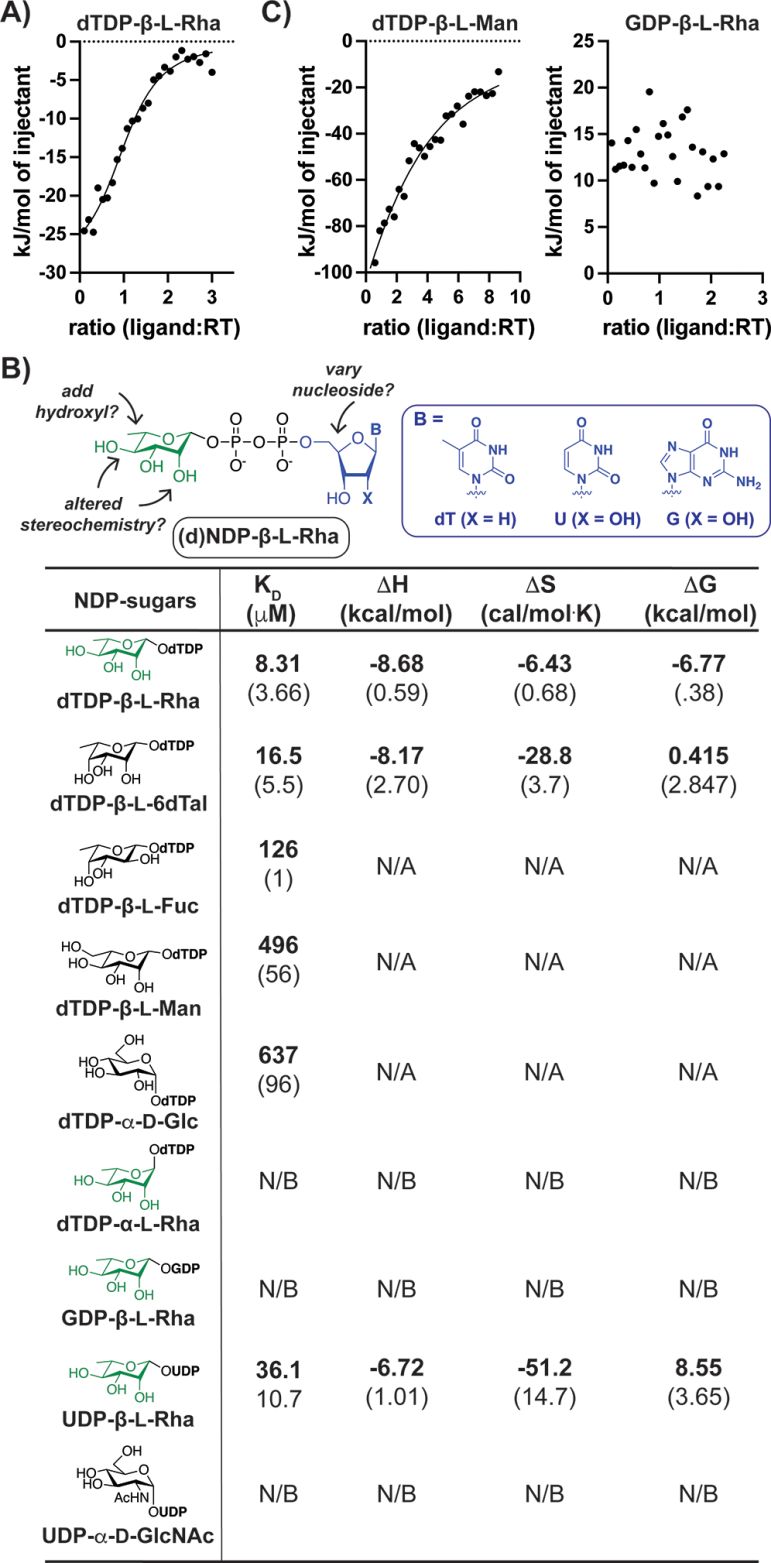
A bacterial RT prefers nucleotide-6-deoxysugars. (A) ITC binding curve of RT (RfbF) with the canonical donor demonstrating micromolar affinity. (B) Summary of ITC results for (d)NDP-sugars indicates that pyrimidine nucleotide-6-deoxy-l-sugars bind with the highest affinities. (C) Representative binding curves for (d)NDP-sugars that show weak (left) or no (right) binding.

We then tested GDP- and UDP-sugars as ligands, since these nucleotides commonly activate cellular sugars.^57−59^ GDP-β-l-Rha showed no binding to RfbF, likely due to the bulky purine (Figure 2C, right). UDP-β-l-Rha bound with an ∼4.5-fold increase in *K*_D_ relative to dTDP-β-l-Rha; however, the cellular metabolite UDP-α-GlcNAc did not bind at μM concentrations, indicating that a C2′-hydroxyl on ribose is accommodated, but additional changes in the sugar moiety are not. These observations suggested that a pyrimidine nucleotide-β-6-deoxysugar could serve as an RT donor.

We next sought to assay WbbL and RfbF using glycolipid acceptors. Inspired by observations that O-Ag biosynthetic enzymes accept substrates with varying lipid structures,^60−62^ we synthesized the following diverse glycolipids to broadly assess the promiscuity of RTs: GlcNAc-pyrophosphate-farnesyl (GlcNAc-PP-C15, **1a**), -geranylgeranyl (-C20, **1b**), -heptaprenyl (-C35, **1c**), and -undecaprenyl (-C55, **1d**) (Figure 3A). Each was accessed based on related glycolipid routes,^41−43,63^ beginning with phosphorylation of peracetylated GlcNAc to form 3,4,6-tri-*O*-acetyl-α-GlcNAc-1-phosphate. The phosphosugar was activated with *N*,*N*-carbonyldiimidazole, and monophosphorylated-isoprenoid coupling was optimized to give the indicated conditions (Figure 3A, table).

**Figure 3:**
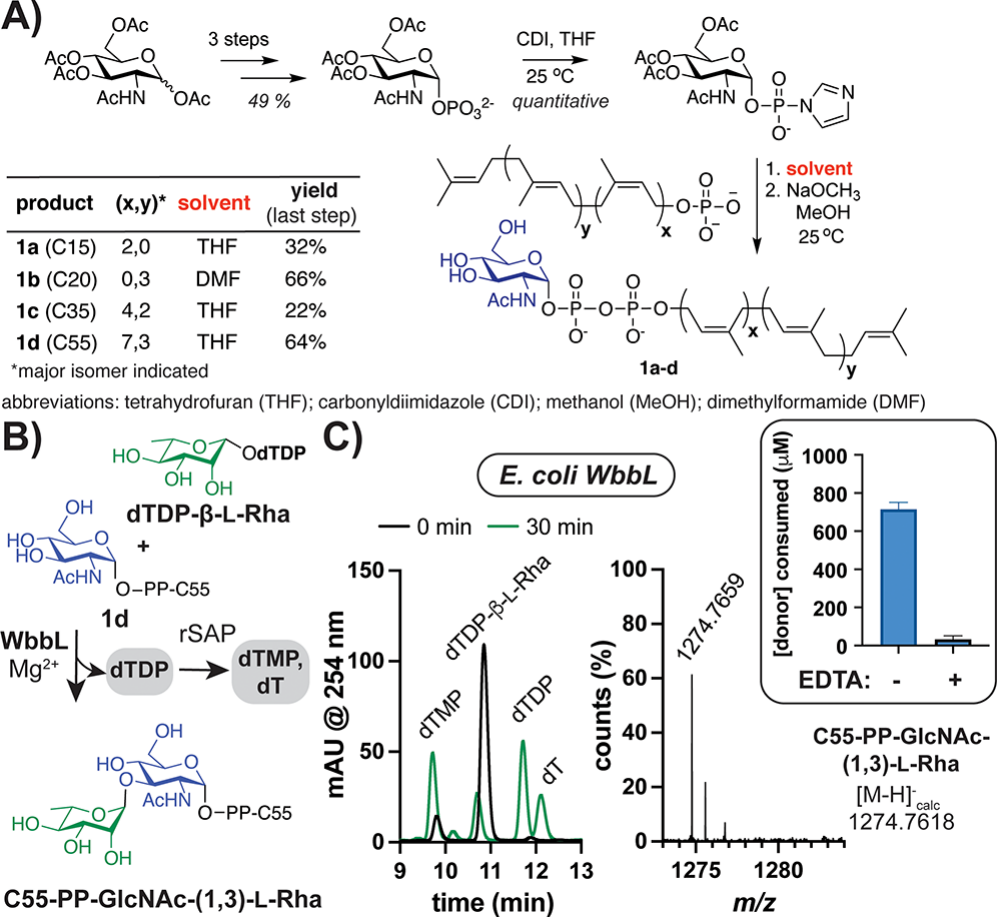
Synthetic glycolipids serve as RT acceptors. (A) Scheme with key steps to GlcNAc-PP-lipid analogs. (B) *E. coli* WbbL utilizes canonical substrates (1 mM each), releasing dTDP that is digested by phosphatase (rSAP). (C) HPLC (left) and HRMS (right) analyses show coupled product formation is metal-dependent (∓ 5 mM EDTA). Error bars represent standard deviation (SD) (*n* = 9). Some dTMP is present in the dTDP-sugar stock.

With putative acceptors in hand, we reconstituted RT activity. Commercial kits for UDP release do not detect dTDP (Figure S14). Hence, we developed HPLC- and high- resolution mass spectrometry (HRMS)-based assays using *E. coli* WbbL and the canonical acceptor (**1d**) with phosphatase (rSAP) to drive the reaction forward (Figures 3B–C, S15).^42^ Unlike previously reported, additional reducing agent was not required for activity.^30^ Since WbbL is a predicted metal-dependent GT-A (Figure S1),^30,64^ reaction and binding buffers contained Mg^2+^. Accordingly, the addition of EDTA inhibited RT reactions (Figures 3C, S16, S17). Heat treatment of WbbL or removal of acceptor resulted in no dTDP release as well (Figure S18a–b). When the noncanonical acceptors Ser or UDP-α-GlcNAc were used with the native donor, no turnover resulted (Figures 4A and S18c), suggesting a glycolipid is needed.^61^ Evaluation of glycolipids **1a**–**d** with dTDP-β-l-Rha revealed that *T. thermophilus* RfbF, and *E. coli* and Mtb WbbL use acceptors with variable lipid chain length, degree of unsaturation, and stereochemistry (Figures 4B, S17, Tables S4–S5). Surprisingly, Mtb WbbL showed optimal turnover with the predominantly *trans*-prenyl C20 substrate, which differs from the canonical acceptor containing *cis* alkenes proximal to the pyrophosphate.^40,65^ Conversely, *E. coli* WbbL preferred its native C55 acceptor. Overall, RTs could utilize glycolipids with chain lengths as short as C15.

**Figure 4:**
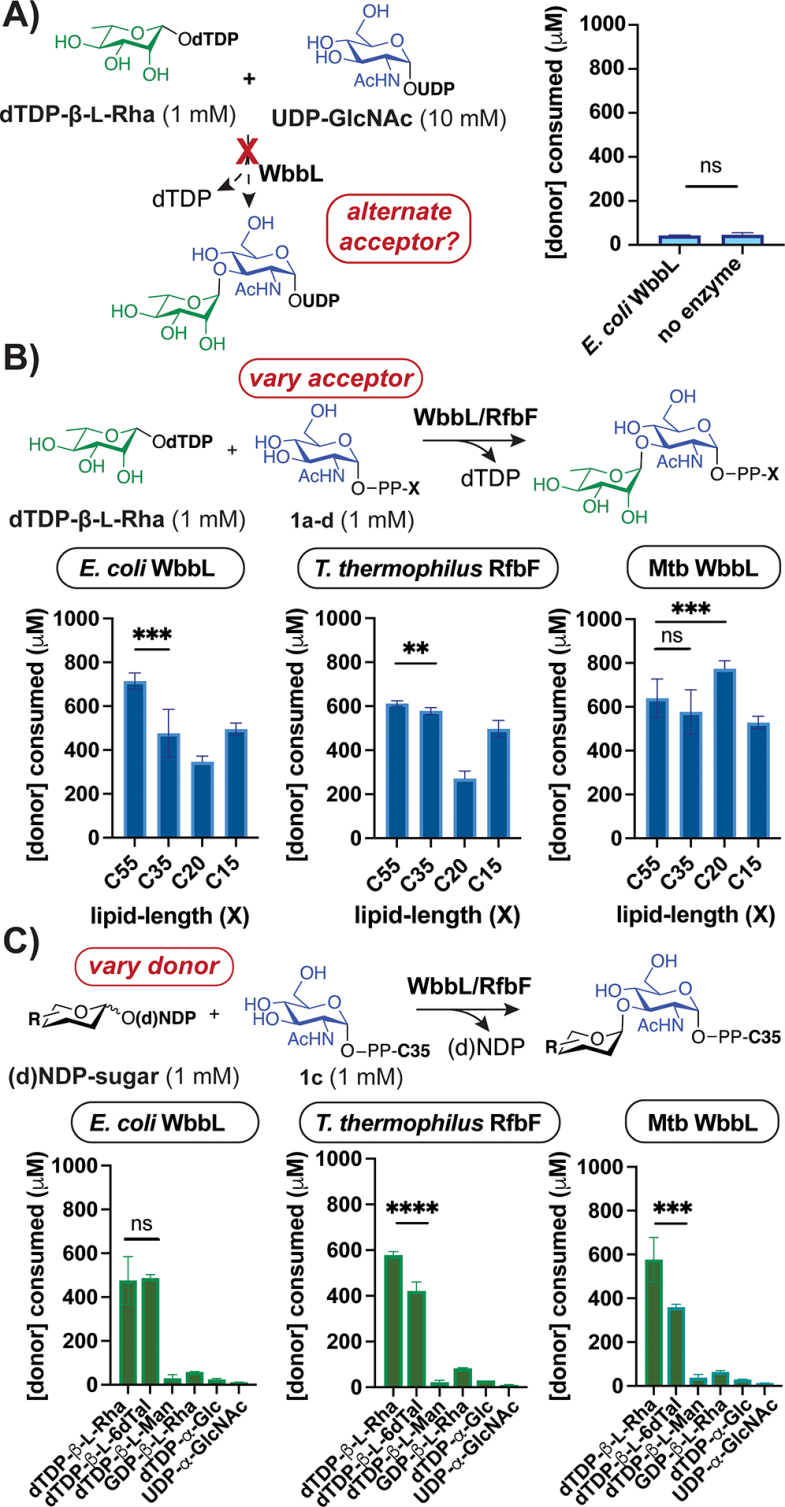
Diverse RTs accept a range of substrates. (A) Comparison of reactions ± *E. coli* WbbL indicates UDP-α-GlcNAc is not used as an acceptor (*t* = 1 h). (B) Evaluation of glycolipid acceptors shows various lipids are tolerated. (C) Analysis of donor substrates validates that bacterial RTs prefer dTDP-6-deoxysugars, not GDP-sugars or (d)NDP-sugars with a C6-hydroxyl (*t* = 30 min for B and C). **** *p* < 0.0001, *** *p* < 0.0002, ** *p* < 0.0021, ns: nonsignificant, paired *t* test used; error bars represent SD (*n* = 3–9).

Due to the improved solubility of C35 versus C55 glycolipid, we next analyzed noncanonical donors using **1c**. ITC analysis suggested that C4 inversion (dTDP-β-l-6dTal) does not affect RT binding, while addition of a C6-hydroxyl or purine nucleotide weakens binding. In the presence of **1c**, turnover of dTDP-β-l-6dTal was comparable to dTDP-β-l-Rha across RTs; however, dTDP-β-l-Man, GDP-β-l-Rha, dTDP-α-Glc, and UDP-α-GlcNAc demonstrated little to no coupling (Figures 4C, S19, Tables S4–S5). RT activity with various donors reflects ITC results and indicates RfbF functions similarly to WbbL.

In *E. coli*, WbbL is required to synthesize oligosaccharide units (O-units), which are flipped, polymerized, and ligated to Lipid A’s core sugars in a model O-Ag (O16) pathway, as well as others^7,13,66^ (Figure 5A). Since O-Ag are virulence factors,^21^ we next aimed to evaluate the cellular consequences of WbbL inhibition. As l-Rha-1-phosphate weakly bound to RfbF, we hypothesized that a reported 6-deoxy-iminosugar (**2**)^67,68^ resembling l-Rha could block the donor site. We synthesized^68^ and then titrated **2** into *E. coli* WbbL reactions containing the native acceptor with the donor present around cellular concentrations.^57^ The resulting half maximal inhibitory concentration (IC_50_) was ∼3 mM (Figure 5B). Direct binding of **2** to RfbF was not detected by ITC, indicating a *K*_D_ > 1 mM (Figure S20). **2** may instead bind the acceptor complex more tightly, as observed for other iminosugars with added nucleotide.^69^ Compared to structurally related l-monosaccharides (Figure 5B), **2** was a more potent WbbL inhibitor than l-Rha (∼53% versus 38% inhibition, respectively), likely because it acts as a transition state mimic.^70^ Lack of inhibition by l-Man reinforced the importance of a C6-deoxy in binding. *E. coli* WbbL docking experiments suggested that **2** binds like the native sugar in the donor site, while l-Man does not (Figure S21).

**Figure 5:**
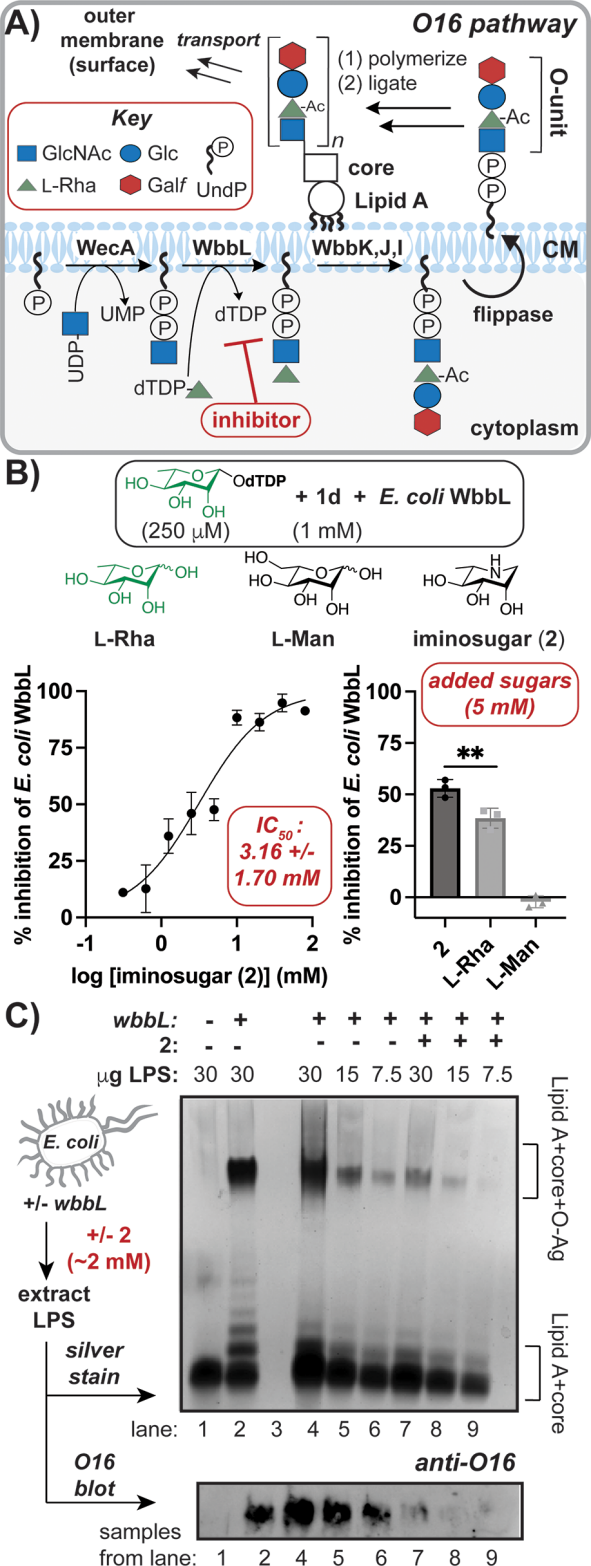
A 6-Deoxy-l-iminosugar inhibits *E. coli* WbbL-dependent O-Ag synthesis. (A) Schematic of O16 O-Ag model pathway, which requires WbbL. (B) *In vitro* inhibition assays indicate iminosugar **2** inhibits WbbL, while l-Man does not (IC_50_ with 95% confidence interval). (C) Addition of **2** to *E. coli* expressing *wbbL* reduces O-Ag synthesis as demonstrated by silver staining (top) and anti-O16 blotting (bottom) of purified LPS; Lipid A standard was utilized (Figure S26). Paired *t* test used; error bars represent SD (*n* = 3).

To evaluate the cellular effect of the iminosugar, an *E. coli* strain expressing *wbbL* (Figure S22) was grown with and without **2**. Following LPS extraction, *E. coli* cells exposed to **2** produced >50% less LPS than untreated samples when an equivalent number of cells was analyzed (Figures S23–S24), even though **2** was not toxic (Figure S25).^71−73^ To assess if the loss of LPS was due to diminished O-Ag, we analyzed extracted LPS by SDS-PAGE followed by silver staining and blotting for O16. As seen in Figure 5C, O-Ag levels decreased in samples containing **2** (lanes 7–9) versus untreated cells (lanes 4–6), with an ∼40% reduction calculated by densitometry (Figure S26).^74^ However, there was not complete loss of O-Ag as in *E. coli* lacking functional *wbbL* (lane 1) compared to cells with native *wbbL* (lane 2). Hence, the iminosugar is not an antibacterial, but impairs WbbL-dependent O-Ag synthesis, which represents a potential antivirulence strategy to impair host infection.^3,44,71,75^

In conclusion, we found that donor recognition by WbbL/RfbF is driven by three factors: the absence of a C6-hydroxyl, and the presence of a β-anomeric center and pyrimidine nucleotide. Binding was enthalpy-driven, with negative free energy only for the canonical donor. We hypothesize that as *K*_D_ increases, an unfavorable entropic contribution results from less desolvation and/or conformational freedom of non-native ligands upon binding.^53,76−83^ Exclusion of activated α-sugars and C6-hydroxyl sugars prevents “incorrect” sugar incorporation into glycans, as cellular UDP-α-GlcNAc and dTDP-α-Glc concentrations (∼200 μM) are below measured *K*_D_ values.^57,59,84−87^ Notably, a natural product RT utilized dTDP-α-Glc as a donor, demonstrating different preferences exist.^88^ WbbL proteins transferred dTDP-β-l-6dTal^89^ to glycolipids, implying that bacterial polysaccharides can be engineered, as explored by others.^7,90−94^ Acceptor analysis indicated that membrane fractions containing endogenous lipids could be used to assess preferences. While RTs can recognize short lipids, *E. coli* WecA, the first transferase in the O-Ag pathway (Figure 5A), requires at least a C35 lipid substrate; hence, shorter lipid lengths are sufficient for later stages of assembly.^60,61,95^ Based on predicted structural similarity across these RTs, it is perhaps unsurprising that consistent substrate recognition trends were observed.

In contrast to well-studied bacterial glycosyltransferases, including the peptidoglycan precursor synthase MurG,^96,97^ few RT inhibitors exist.^67,68^ Since MurG uses the donor UDP-α-GlcNAc, methods to modulate MurG are not transferable to WbbL. Further, while the toxin colicin M depletes O-Ag by targeting carrier lipid modification, it also affects essential biosynthetic pathways.^98^ We found that **2** reduces cellular O-Ag levels and is nontoxic; however, like other iminosugars, **2** requires improvement before assessing virulence effects in a host.^67,99^ Our findings will inform substrate analogue design to discover more potent inhibitors, and probe surface glycan biosynthesis mechanisms and host–pathogen interactions.

## Supporting Information Available

Tables S1–S8, Figures S1–S26; description and schemes of experimental methods for nucleotide sugar and glycolipid syntheses; compound characterization (NMR, mass spectrometry); methods for plasmid construction, protein expression and purification (Tables S6–S8 contain information on plasmids, primers and strains that were utilized); and additional analyses of enzymatic activities of WbbL and RfbF, confirmation of coupled products (HPLC and mass spectrometry analyses), and bioinformatic analyses (PDF)

## References

[ref1] Silhavy T. J.; Kahne D.; Walker S. The bacterial cell envelope. Cold Spring Harb. Perspect. Biol. 2010, 2 (5), a000414 10.1101/cshperspect.a000414. 20452953 PMC2857177

[ref2] Herget S.; Toukach P. V.; Ranzinger R.; Hull W. E.; Knirel Y. A.; von der Lieth C. W. Statistical analysis of the Bacterial Carbohydrate Structure Data Base (BCSDB): characteristics and diversity of bacterial carbohydrates in comparison with mammalian glycans. BMC Struct. Biol. 2008, 8, 35 10.1186/1472-6807-8-35. 18694500 PMC2543016

[ref3] Reeves P. Role of O-antigen variation in the immune response. Trends Microbiol. 1995, 3 (10), 381–386. 10.1016/S0966-842X(00)88983-0. 8564356

[ref4] McCallum K. L.; Schoenhals G.; Laakso D.; Clarke B.; Whitfield C. A high-molecular-weight fraction of smooth lipopolysaccharide in Klebsiella serotype O1:K20 contains a unique O-antigen epitope and determines resistance to nonspecific serum killing. Infect. Immun. 1989, 57 (12), 3816–3822. 10.1128/iai.57.12.3816-3822.1989. 2478478 PMC259910

[ref5] Mostowy R. J.; Holt K. E. Diversity-Generating Machines: Genetics of Bacterial Sugar-Coating. Trends Microbiol. 2018, 26 (12), 1008–1021. 10.1016/j.tim.2018.06.006. 30037568 PMC6249986

[ref6] Bertani B.; Ruiz N. Function and Biogenesis of Lipopolysaccharides. EcoSal Plus 2018, 8 (1), 1–19. 10.1128/ecosalplus.ESP-0001-2018. PMC6091223 30066669

[ref7] Zheng M.; Zheng M.; Epstein S.; Harnagel A. P.; Kim H.; Lupoli T. J. Chemical Biology Tools for Modulating and Visualizing Gram-Negative Bacterial Surface Polysaccharides. ACS Chem. Biol. 2021, 16 (10), 1841–1865. 10.1021/acschembio.1c00341. 34569792

[ref8] Reeves P. R., Cunneen M. M. Biosynthesis of O-antigen chains and assembly. Microbial Glycobiology; Holst O., Brennan P. J., von Itzstein M., Eds.; Academic Press: 2010; pp 319–335.

[ref9] Imperiali B. Bacterial carbohydrate diversity - a Brave New World. Curr. Opin. Chem. Biol. 2019, 53, 1–8. 10.1016/j.cbpa.2019.04.026. 31176085 PMC6893104

[ref10] Tra V. N.; Dube D. H. Glycans in pathogenic bacteria--potential for targeted covalent therapeutics and imaging agents. Chem. Commun. 2014, 50 (36), 4659–4673. 10.1039/C4CC00660G. PMC4049282 24647371

[ref11] Clark E. L.; Emmadi M.; Krupp K. L.; Podilapu A. R.; Helble J. D.; Kulkarni S. S.; Dube D. H. Development of Rare Bacterial Monosaccharide Analogs for Metabolic Glycan Labeling in Pathogenic Bacteria. ACS Chem. Biol. 2016, 11 (12), 3365–3373. 10.1021/acschembio.6b00790. 27766829 PMC5161589

[ref12] Adibekian A.; Stallforth P.; Hecht M.-L.; Werz D. B.; Gagneux P.; Seeberger P. H. Comparative bioinformatics analysis of the mammalian and bacterial glycomes. Chem. Sci. 2011, 2 (2), 337–344. 10.1039/C0SC00322K.

[ref13] Liu B.; Furevi A.; Perepelov A. V.; Guo X.; Cao H.; Wang Q.; Reeves P. R.; Knirel Y. A.; Wang L.; Widmalm G. Structure and genetics of *Escherichia coli* O antigens. FEMS Microbiol. Rev. 2020, 44 (6), 655–683. 10.1093/femsre/fuz028. 31778182 PMC7685785

[ref14] Toukach P. V.; Egorova K. S. Carbohydrate structure database merged from bacterial, archaeal, plant and fungal parts. Nucleic Acids Res. 2016, 44 (D1), D1229–D1236. 10.1093/nar/gkv840. 26286194 PMC4702937

[ref15] Rojas-Macias M. A.; Stahle J.; Lutteke T.; Widmalm G. Development of the ECODAB into a relational database for *Escherichia coli* O-antigens and other bacterial polysaccharides. Glycobiology 2015, 25 (3), 341–347. 10.1093/glycob/cwu116. 25352573

[ref16] Brennan P. J.; Nikaido H. The envelope of mycobacteria. Annu. Rev. Biochem. 1995, 64, 29–63. 10.1146/annurev.bi.64.070195.000333. 7574484

[ref17] Mills J. A.; Motichka K.; Jucker M.; Wu H. P.; Uhlik B. C.; Stern R. J.; Scherman M. S.; Vissa V. D.; Pan F.; Kundu M.; Ma Y. F.; McNeil M. Inactivation of the mycobacterial rhamnosyltransferase, which is needed for the formation of the arabinogalactan-peptidoglycan linker, leads to irreversible loss of viability. J. Biol. Chem. 2004, 279 (42), 43540–43546. 10.1074/jbc.M407782200. 15294902

[ref18] Wu Q.; Zhou P.; Qian S.; Qin X.; Fan Z.; Fu Q.; Zhan Z.; Pei H. Cloning, expression, identification and bioinformatics analysis of Rv3265c gene from *Mycobacterium tuberculosis in Escherichia coli*. Asian Pac. J. Trop. Med. 2011, 4 (4), 266–270. 10.1016/S1995-7645(11)60083-7. 21771467

[ref19] Grzegorzewicz A. E.; de Sousa-d’Auria C.; McNeil M. R.; Huc-Claustre E.; Jones V.; Petit C.; Angala S. K.; Zemanova J.; Wang Q.; Belardinelli J. M.; Gao Q.; Ishizaki Y.; Mikusova K.; Brennan P. J.; Ronning D. R.; Chami M.; Houssin C.; Jackson M. Assembling of the *Mycobacterium tuberculosis* Cell Wall Core. J. Biol. Chem. 2016, 291 (36), 18867–18879. 10.1074/jbc.M116.739227. 27417139 PMC5009262

[ref20] Rahlwes K. C.; Sparks I. L.; Morita Y. S. Cell Walls and Membranes of Actinobacteria. Subcell. Biochem. 2019, 92, 417–469. 10.1007/978-3-030-18768-2_13. 31214994

[ref21] Raetz C. R.; Whitfield C. Lipopolysaccharide endotoxins. Annu. Rev. Biochem. 2002, 71, 635–700. 10.1146/annurev.biochem.71.110601.135414. 12045108 PMC2569852

[ref22] Valvano M. A. Export of O-specific lipopolysaccharide. Front. Biosci. 2003, 8 (6), 452–471. 10.2741/1079. 12700099

[ref23] Jumper J.; Evans R.; Pritzel A.; Green T.; Figurnov M.; Ronneberger O.; Tunyasuvunakool K.; Bates R.; Zidek A.; Potapenko A.; Bridgland A.; Meyer C.; Kohl S. A. A.; Ballard A. J.; Cowie A.; Romera-Paredes B.; Nikolov S.; Jain R.; Adler J.; Back T.; Petersen S.; Reiman D.; Clancy E.; Zielinski M.; Steinegger M.; Pacholska M.; Berghammer T.; Bodenstein S.; Silver D.; Vinyals O.; Senior A. W.; Kavukcuoglu K.; Kohli P.; Hassabis D. Highly accurate protein structure prediction with AlphaFold. Nature 2021, 596 (7873), 583–589. 10.1038/s41586-021-03819-2. 34265844 PMC8371605

[ref24] Varadi M.; Anyango S.; Deshpande M.; Nair S.; Natassia C.; Yordanova G.; Yuan D.; Stroe O.; Wood G.; Laydon A.; Zidek A.; Green T.; Tunyasuvunakool K.; Petersen S.; Jumper J.; Clancy E.; Green R.; Vora A.; Lutfi M.; Figurnov M.; Cowie A.; Hobbs N.; Kohli P.; Kleywegt G.; Birney E.; Hassabis D.; Velankar S. AlphaFold Protein Structure Database: massively expanding the structural coverage of protein-sequence space with high-accuracy models. Nucleic Acids Res. 2022, 50 (D1), D439–D444. 10.1093/nar/gkab1061. 34791371 PMC8728224

[ref25] Rubires X.; Saigi F.; Pique N.; Climent N.; Merino S.; Alberti S.; Tomas J. M.; Regue M. A gene (wbbL) from *Serratia marcescens* N28b (O4) complements the rfb-50 mutation of *Escherichia coli* K-12 derivatives. J. Bacteriol. 1997, 179 (23), 7581–7586. 10.1128/jb.179.23.7581-7586.1997. 9393727 PMC179713

[ref26] Izquierdo L.; Merino S.; Regue M.; Rodriguez F.; Tomas J. M. Synthesis of a *Klebsiella pneumoniae* O-antigen heteropolysaccharide (O12) requires an ABC 2 transporter. J. Bacteriol. 2003, 185 (5), 1634–1641. 10.1128/JB.185.5.1634-1641.2003. 12591881 PMC148082

[ref27] Han X.; Shi Q.; Mao Y.; Quan J.; Zhang P.; Lan P.; Jiang Y.; Zhao D.; Wu X.; Hua X.; Yu Y. Emergence of Ceftazidime/Avibactam and Tigecycline Resistance in Carbapenem-Resistant *Klebsiella pneumoniae* Due to In-Host Microevolution. Front. Cell. Infect. Microbiol. 2021, 11, 757470 10.3389/fcimb.2021.757470. 34760723 PMC8573091

[ref28] Shoji M.; Sato K.; Yukitake H.; Kamaguchi A.; Sasaki Y.; Naito M.; Nakayama K. Identification of genes encoding glycosyltransferases involved in lipopolysaccharide synthesis in *Porphyromonas gingivalis*. Mol. Oral Microbiol. 2018, 33 (1), 68–80. 10.1111/omi.12200. 28972686

[ref29] Pettit N.; Styslinger T.; Mei Z.; Han W.; Zhao G.; Wang P. G. Characterization of WbiQ: An alpha1,2-fucosyltransferase from *Escherichia coli* O127:K63(B8), and synthesis of H-type 3 blood group antigen. Biochem. Biophys. Res. Commun. 2010, 402 (2), 190–195. 10.1016/j.bbrc.2010.08.087. 20801103 PMC3441828

[ref30] Grzegorzewicz A. E.; Ma Y.; Jones V.; Crick D.; Liav A.; McNeil M. R. Development of a microtitre plate-based assay for lipid-linked glycosyltransferase products using the mycobacterial cell wall rhamnosyltransferase WbbL. Microbiology 2008, 154 (12), 3724–3730. 10.1099/mic.0.2008/023366-0. 19047740 PMC2717732

[ref31] Zheng M.; Zheng M.; Lupoli T. J. Expanding the Substrate Scope of a Bacterial Nucleotidyltransferase via Allosteric Mutations. ACS Infect. Dis. 2022, 8 (10), 2035–2044. 10.1021/acsinfecdis.2c00402. 36106727 PMC10322145

[ref32] Sabesan S.; Neira S. Synthesis of glycosyl phosphates and azides. Carbohydr. Res. 1992, 223, 169–185. 10.1016/0008-6215(92)80015-S.

[ref33] Kirschning A.; Bechthold A. F.-W.; Rohr J. Chemical and biochemical aspects of deoxysugars and deoxy sugar oligosaccharides. Bioorganic Chemistry; Springer: Berlin, Heidelberg, 1997; Vol. 188, pp 1–84.

[ref34] Zhao Y.; Thorson J. S. A Methodological Comparison: The Advantage of Phosphorimidates in Expanding the Sugar Nucleotide Repertoire. J. Org. Chem. 1998, 63 (21), 7568–7572. 10.1021/jo981265n. 11672420

[ref35] Oberthur M.; Leimkuhler C.; Kahne D. A practical method for the stereoselective generation of β-2-deoxy glycosyl phosphates. Org. Lett. 2004, 6 (17), 2873–2876. 10.1021/ol049187f. 15330636

[ref36] Timmons S. C.; Jakeman D. L. Stereospecific synthesis of sugar-1-phosphates and their conversion to sugar nucleotides. Carbohydr. Res. 2008, 343 (5), 865–874. 10.1016/j.carres.2008.01.046. 18299123

[ref37] Wagner G. K.; Pesnot T.; Field R. A. A survey of chemical methods for sugar-nucleotide synthesis. Nat. Prod. Rep. 2009, 26 (9), 1172–1194. 10.1039/b909621n. 19693414

[ref38] Wagstaff B. A.; Rejzek M.; Kuhaudomlarp S.; Hill L.; Mascia I.; Nepogodiev S. A.; Dorfmueller H. C.; Field R. A. Discovery of an RmlC/D fusion protein in the microalga *Prymnesium parvum* and its implications for NDP-β-l-rhamnose biosynthesis in microalgae. J. Biol. Chem. 2019, 294 (23), 9172–9185. 10.1074/jbc.RA118.006440. 31010825 PMC6556577

[ref39] Chen L.; Men H.; Ha S.; Ye X. Y.; Brunner L.; Hu Y.; Walker S. Intrinsic lipid preferences and kinetic mechanism of *Escherichia coli* MurG. Biochemistry 2002, 41 (21), 6824–6833. 10.1021/bi0256678. 12022887

[ref40] Ye X. Y.; Lo M. C.; Brunner L.; Walker D.; Kahne D.; Walker S. Better substrates for bacterial transglycosylases. J. Am. Chem. Soc. 2001, 123 (13), 3155–3156. 10.1021/ja010028q. 11457035

[ref41] Liu F.; Vijayakrishnan B.; Faridmoayer A.; Taylor T. A.; Parsons T. B.; Bernardes G. J.; Kowarik M.; Davis B. G. Rationally designed short polyisoprenol-linked PglB substrates for engineered polypeptide and protein N-glycosylation. J. Am. Chem. Soc. 2014, 136 (2), 566–569. 10.1021/ja409409h. 24377322

[ref42] Li L.; Woodward R. L.; Han W.; Qu J.; Song J.; Ma C.; Wang P. G. Chemoenzymatic synthesis of the bacterial polysaccharide repeating unit undecaprenyl pyrophosphate and its analogs. Nat. Protoc. 2016, 11 (7), 1280–1298. 10.1038/nprot.2016.067. 27336706 PMC5953200

[ref43] Hsu T. W.; Hsu H. C.; Chan H. Y.; Fang J. M. A Terpyridine Zinc Complex for Selective Detection of Lipid Pyrophosphates: A Model System for Monitoring Bacterial O- and N-Transglycosylations. J. Org. Chem. 2020, 85 (19), 12747–12753. 10.1021/acs.joc.0c01252. 32885656

[ref44] Hotinger J. A.; Morris S. T.; May A. E. The Case against Antibiotics and for Anti-Virulence Therapeutics. Microorganisms 2021, 9 (10), 2049–2101. 10.3390/microorganisms9102049. 34683370 PMC8537500

[ref45] Zheng M.; Zheng M.; Kim H.; Lupoli T. Feedback inhibition of bacterial nucleotidylransferases by rare nucleotide l-sugars restricts substrate promiscuity. J. Am. Chem. Soc. 2023, 10.1021/jacs.3c02319. PMC10375476 37283497

[ref47] Wagstaff B. A.; Zorzoli A.; Dorfmueller H. C. NDP-rhamnose biosynthesis and rhamnosyltransferases: building diverse glycoconjugates in nature. Biochem. J. 2021, 478 (4), 685–701. 10.1042/BCJ20200505. 33599745

[ref48] Lombard V.; Golaconda Ramulu H.; Drula E.; Coutinho P. M.; Henrissat B. The carbohydrate-active enzymes database (CAZy) in 2013. Nucleic Acids Res. 2014, 42 (D1), D490–D495. 10.1093/nar/gkt1178. 24270786 PMC3965031

[ref49] Drula E.; Garron M. L.; Dogan S.; Lombard V.; Henrissat B.; Terrapon N. The carbohydrate-active enzyme database: functions and literature. Nucleic Acids Res. 2022, 50 (D1), D571–D577. 10.1093/nar/gkab1045. 34850161 PMC8728194

[ref50] Sievers F.; Wilm A.; Dineen D.; Gibson T. J.; Karplus K.; Li W.; Lopez R.; McWilliam H.; Remmert M.; Soding J.; Thompson J. D.; Higgins D. G. Fast, scalable generation of high-quality protein multiple sequence alignments using Clustal Omega. Mol. Syst. Biol. 2011, 7, 539 10.1038/msb.2011.75. 21988835 PMC3261699

[ref51] Kilinc M.; Jia K.; Jernigan R. L. Improved global protein homolog detection with major gains in function identification. Proc. Natl. Acad. Sci. U. S. A. 2023, 120 (9), e2211823120 10.1073/pnas.2211823120. 36827259 PMC9992864

[ref52] Turnbull W. B.; Daranas A. H. On the value of c: can low affinity systems be studied by isothermal titration calorimetry?. J. Am. Chem. Soc. 2003, 125 (48), 14859–14866. 10.1021/ja036166s. 14640663

[ref53] He C.; Liu N.; Li F.; Jia X.; Peng H.; Liu Y.; Xiao Y. Complex Structure of *Pseudomonas aeruginosa* Arginine Rhamnosyltransferase EarP with Its Acceptor Elongation Factor P. J. Bacteriol. 2019, 201 (13), e00128–19. 10.1128/JB.00128-19. 31010899 PMC6560138

[ref54] Steiner K.; Hagelueken G.; Messner P.; Schaffer C.; Naismith J. H. Structural basis of substrate binding in WsaF, a rhamnosyltransferase from *Geobacillus stearothermophilus*. J. Mol. Biol. 2010, 397 (2), 436–447. 10.1016/j.jmb.2010.01.035. 20097205 PMC3898925

[ref55] Isiorho E. A.; Liu H. W.; Keatinge-Clay A. T. Structural studies of the spinosyn rhamnosyltransferase, SpnG. Biochemistry 2012, 51 (6), 1213–1222. 10.1021/bi201860q. 22283226 PMC3285110

[ref56] Zong G.; Li J.; Gao Y.; Fei S.; Liu X.; Wang X.; Shen Y. Overexpression, purification, biochemical and structural characterization of rhamnosyltransferase UGT89C1 from *Arabidopsis thaliana*. Protein Expr. Purif. 2019, 156, 44–49. 10.1016/j.pep.2018.12.007. 30597216

[ref57] Bochner B. R.; Ames B. N. Complete analysis of cellular nucleotides by two-dimensional thin layer chromatography. J. Biol. Chem. 1982, 257 (16), 9759–69. 10.1016/S0021-9258(18)34138-3. 6286632

[ref58] Thibodeaux C. J.; Melancon C. E. 3rd; Liu H. W. Natural-product sugar biosynthesis and enzymatic glycodiversification. Angew. Chem., Int. Ed. 2008, 47 (51), 9814–9859. 10.1002/anie.200801204. PMC2796923 19058170

[ref59] Samuel G.; Reeves P. Biosynthesis of O-antigens: genes and pathways involved in nucleotide sugar precursor synthesis and O-antigen assembly. Carbohydr. Res. 2003, 338 (23), 2503–2519. 10.1016/j.carres.2003.07.009. 14670712

[ref60] Woodward R.; Yi W.; Li L.; Zhao G.; Eguchi H.; Sridhar P. R.; Guo H.; Song J. K.; Motari E.; Cai L.; Kelleher P.; Liu X.; Han W.; Zhang W.; Ding Y.; Li M.; Wang P. G. In vitro bacterial polysaccharide biosynthesis: defining the functions of Wzy and Wzz. Nat. Chem. Biol. 2010, 6 (6), 418–423. 10.1038/nchembio.351. 20418877 PMC2921718

[ref61] Yi W.; Yao Q.; Zhang Y.; Motari E.; Lin S.; Wang P. G. The wbnH gene of *Escherichia coli* O86:H2 encodes an α-1,3-*N*-acetylgalactosaminyl transferase involved in the O-repeating unit biosynthesis. Biochem. Biophys. Res. Commun. 2006, 344 (2), 631–639. 10.1016/j.bbrc.2006.03.181. 16630548

[ref62] Reid A. J.; Scarbrough B. A.; Williams T. C.; Gates C. E.; Eade C. R.; Troutman J. M. General Utilization of Fluorescent Polyisoprenoids with Sugar Selective Phosphoglycosyltransferases. Biochemistry 2020, 59 (4), 615–626. 10.1021/acs.biochem.9b01026. 31876413 PMC7132332

[ref63] Bosco M.; Massarweh A.; Iatmanen-Harbi S.; Bouhss A.; Chantret I.; Busca P.; Moore S. E.; Gravier-Pelletier C. Synthesis and biological evaluation of chemical tools for the study of Dolichol Linked Oligosaccharide Diphosphatase (DLODP). Eur. J. Med. Chem. 2017, 125, 952–964. 10.1016/j.ejmech.2016.10.013. 27769035

[ref64] Lairson L. L.; Henrissat B.; Davies G. J.; Withers S. G. Glycosyltransferases: structures, functions, and mechanisms. Annu. Rev. Biochem. 2008, 77, 521–555. 10.1146/annurev.biochem.76.061005.092322. 18518825

[ref65] Mahapatra S.; Yagi T.; Belisle J. T.; Espinosa B. J.; Hill P. J.; McNeil M. R.; Brennan P. J.; Crick D. C. Mycobacterial lipid II is composed of a complex mixture of modified muramyl and peptide moieties linked to decaprenyl phosphate. J. Bacteriol. 2005, 187 (8), 2747–2757. 10.1128/JB.187.8.2747-2757.2005. 15805521 PMC1070386

[ref66] Stenutz R.; Weintraub A.; Widmalm G. The structures of *Escherichia coli* O-polysaccharide antigens. FEMS Microbiol. Rev. 2006, 30 (3), 382–403. 10.1111/j.1574-6976.2006.00016.x. 16594963

[ref67] Lucas R.; Balbuena P.; Errey J. C.; Squire M. A.; Gurcha S. S.; McNeil M.; Besra G. S.; Davis B. G. Glycomimetic inhibitors of mycobacterial glycosyltransferases: targeting the TB cell wall. Chembiochem 2008, 9 (14), 2197–2199. 10.1002/cbic.200800189. 18780384

[ref68] Dharuman S.; Wang Y.; Crich D. Alternative synthesis and antibacterial evaluation of 1,5-dideoxy-1,5-imino-l-rhamnitol. Carbohydr. Res. 2016, 419, 29–32. 10.1016/j.carres.2015.10.015. 26623949 PMC4698172

[ref69] Qiao L.; Murray B. W.; Shimazaki M.; Schultz J.; Wong C. H. Synergistic Inhibition of Human α-1,3-Fucosyltransferase V. J. Am. Chem. Soc. 1996, 118 (33), 7653–7662. 10.1021/ja960274f.

[ref70] Conforti I.; Marra A. Iminosugars as glycosyltransferase inhibitors. Org. Biomol. Chem. 2021, 19 (25), 5439–5475. 10.1039/D1OB00382H. 33881114

[ref71] Jorgenson M. A.; Young K. D. Interrupting Biosynthesis of O Antigen or the Lipopolysaccharide Core Produces Morphological Defects in *Escherichia coli* by Sequestering Undecaprenyl Phosphate. J. Bacteriol. 2016, 198 (22), 3070–3079. 10.1128/JB.00550-16. 27573014 PMC5075036

[ref72] Westphal O.; Jann K. Bacterial lipopolysaccharides extraction with phenol-water and further applications of the procedure. Methods in Carbohydrate Chemistry 1965, 5, 83–91.

[ref73] Tsai C. M.; Frasch C. E. A sensitive silver stain for detecting lipopolysaccharides in polyacrylamide gels. Anal. Biochem. 1982, 119 (1), 115–119. 10.1016/0003-2697(82)90673-X. 6176137

[ref74] King J. D.; Berry S.; Clarke B. R.; Morris R. J.; Whitfield C. Lipopolysaccharide O antigen size distribution is determined by a chain extension complex of variable stoichiometry in *Escherichia coli* O9a. Proc. Natl. Acad. Sci. U. S. A. 2014, 111 (17), 6407–6412. 10.1073/pnas.1400814111. 24733938 PMC4035927

[ref75] Stevenson G.; Neal B.; Liu D.; Hobbs M.; Packer N. H.; Batley M.; Redmond J. W.; Lindquist L.; Reeves P. Structure of the O antigen of *Escherichia coli* K-12 and the sequence of its rfb gene cluster. J. Bacteriol. 1994, 176 (13), 4144–4156. 10.1128/jb.176.13.4144-4156.1994. 7517391 PMC205614

[ref76] Larson M. R.; Biddle K.; Gorman A.; Boutom S.; Rosenshine I.; Saper M. A. *Escherichia coli* O127 group 4 capsule proteins assemble at the outer membrane. PLoS One 2021, 16 (11), e0259900 10.1371/journal.pone.0259900. 34780538 PMC8592465

[ref77] Thiyagarajan N.; Pham T. T.; Stinson B.; Sundriyal A.; Tumbale P.; Lizotte-Waniewski M.; Brew K.; Acharya K. R. Structure of a metal-independent bacterial glycosyltransferase that catalyzes the synthesis of histo-blood group A antigen. Sci. Rep. 2012, 2, 940 10.1038/srep00940. 23230506 PMC3516806

[ref78] Nurisso A.; Blanchard B.; Audfray A.; Rydner L.; Oscarson S.; Varrot A.; Imberty A. Role of water molecules in structure and energetics of *Pseudomonas aeruginosa* lectin I interacting with disaccharides. J. Biol. Chem. 2010, 285 (26), 20316–20327. 10.1074/jbc.M110.108340. 20410292 PMC2888444

[ref79] Esposito D.; Gunster R. A.; Martino L.; El Omari K.; Wagner A.; Thurston T. L. M.; Rittinger K. Structural basis for the glycosyltransferase activity of the *Salmonella* effector SseK3. J. Biol. Chem. 2018, 293 (14), 5064–5078. 10.1074/jbc.RA118.001796. 29449376 PMC5892559

[ref80] Perez S.; Tvaroska I. Carbohydrate-protein interactions: molecular modeling insights. Adv. Carbohydr. Chem. Biochem. 2014, 71, 9–136. 10.1016/B978-0-12-800128-8.00001-7. 25480504

[ref81] Navarra G.; Zihlmann P.; Jakob R. P.; Stangier K.; Preston R. C.; Rabbani S.; Smiesko M.; Wagner B.; Maier T.; Ernst B. Carbohydrate-Lectin Interactions: An Unexpected Contribution to Affinity. Chembiochem 2017, 18 (6), 539–544. 10.1002/cbic.201600615. 28076665

[ref82] Hariharan P.; Balasubramaniam D.; Peterkofsky A.; Kaback H. R.; Guan L. Thermodynamic mechanism for inhibition of lactose permease by the phosphotransferase protein IIAGlc. Proc. Natl. Acad. Sci. U. S. A. 2015, 112 (8), 2407–2412. 10.1073/pnas.1500891112. 25675534 PMC4345578

[ref83] Velazquez Campoy A.; Freire E. ITC in the post-genomic era···? Priceless. Biophys. Chem. 2005, 115 (2–3), 115–124. 10.1016/j.bpc.2004.12.015. 15752592

[ref84] Qu H.; Xin Y.; Dong X.; Ma Y. An rmlA gene encoding d-glucose-1-phosphate thymidylyltransferase is essential for mycobacterial growth. FEMS Microbiol. Lett. 2007, 275 (2), 237–243. 10.1111/j.1574-6968.2007.00890.x. 17784859

[ref85] Giraud M. F.; Naismith J. H. The rhamnose pathway. Curr. Opin. Struct. Biol. 2000, 10 (6), 687–696. 10.1016/S0959-440X(00)00145-7. 11114506

[ref86] Ahmadipour S.; Beswick L.; Miller G. J. Recent advances in the enzymatic synthesis of sugar-nucleotides using nucleotidylyltransferases and glycosyltransferases. Carbohydr. Res. 2018, 469, 38–47. 10.1016/j.carres.2018.09.002. 30265902

[ref87] Blankenfeldt W.; Asuncion M.; Lam J. S.; Naismith J. H. The structural basis of the catalytic mechanism and regulation of glucose-1-phosphate thymidylyltransferase (RmlA). EMBO J. 2000, 19 (24), 6652–6663. 10.1093/emboj/19.24.6652. 11118200 PMC305900

[ref88] Chen Y. L.; Chen Y. H.; Lin Y. C.; Tsai K. C.; Chiu H. T. Functional characterization and substrate specificity of spinosyn rhamnosyltransferase by in vitro reconstitution of spinosyn biosynthetic enzymes. J. Biol. Chem. 2009, 284 (11), 7352–7363. 10.1074/jbc.M808441200. 19126547 PMC2652332

[ref89] Gaugler R. W.; Gabriel O. Biological mechanisms involved in the formation of deoxy sugars. VII. Biosynthesis of 6-deoxy-l-talose. J. Biol. Chem. 1973, 248 (17), 6041–6049. 10.1016/S0021-9258(19)43505-9. 4199258

[ref90] Yi W.; Liu X.; Li Y.; Li J.; Xia C.; Zhou G.; Zhang W.; Zhao W.; Chen X.; Wang P. G. Remodeling bacterial polysaccharides by metabolic pathway engineering. Proc. Natl. Acad. Sci. U. S. A. 2009, 106 (11), 4207–4212. 10.1073/pnas.0812432106. 19251666 PMC2657399

[ref91] Weyant K. B.; Mills D. C.; DeLisa M. P. Engineering a new generation of carbohydrate-based vaccines. Curr. Opin. Chem. Eng. 2018, 19, 77–85. 10.1016/j.coche.2017.12.009. 30568873 PMC6296769

[ref92] Dumont A.; Malleron A.; Awwad M.; Dukan S.; Vauzeilles B. Click-mediated labeling of bacterial membranes through metabolic modification of the lipopolysaccharide inner core. Angew. Chem., Int. Ed. 2012, 51 (13), 3143–3146. 10.1002/anie.201108127. 22323101

[ref93] Andolina G.; Wei R.; Liu H.; Zhang Q.; Yang X.; Cao H.; Chen S.; Yan A.; Li X. D.; Li X. Metabolic Labeling of Pseudaminic Acid-Containing Glycans on Bacterial Surfaces. ACS Chem. Biol. 2018, 13 (10), 3030–3037. 10.1021/acschembio.8b00822. 30230814

[ref94] Moulton K. D.; Adewale A. P.; Carol H. A.; Mikami S. A.; Dube D. H. Metabolic Glycan Labeling-Based Screen to Identify Bacterial Glycosylation Genes. ACS Infect. Dis. 2020, 6 (12), 3247–3259. 10.1021/acsinfecdis.0c00612. 33186014 PMC7808405

[ref95] Al-Dabbagh B.; Mengin-Lecreulx D.; Bouhss A. Purification and characterization of the bacterial UDP-GlcNAc:undecaprenyl-phosphate GlcNAc-1-phosphate transferase WecA. J. Bacteriol. 2008, 190 (21), 7141–7146. 10.1128/JB.00676-08. 18723618 PMC2580700

[ref96] Ha S.; Gross B.; Walker S. E. Coli MurG: a paradigm for a superfamily of glycosyltransferases. Curr. Drug Targets Infect. Disord. 2001, 1 (2), 201–213. 10.2174/1568005014606116. 12455415

[ref97] Liu Y.; Breukink E. The Membrane Steps of Bacterial Cell Wall Synthesis as Antibiotic Targets. Antibiotics 2016, 5 (3), 28–50. 10.3390/antibiotics5030028. 27571111 PMC5039524

[ref98] Harkness R. E.; Braun V. Inhibition of lipopolysaccharide O-antigen synthesis by colicin M. J. Biol. Chem. 1989, 264 (25), 14716–14722. 10.1016/S0021-9258(18)63757-3. 2475490

[ref99] Compain P.; Martin O. R. Carbohydrate mimetics-based glycosyltransferase inhibitors. Bioorg. Med. Chem. 2001, 9 (12), 3077–3092. 10.1016/S0968-0896(01)00176-6. 11711283

