# Supplementary material for: Preference of Bacterial Rhamnosyltransferases for 6-Deoxysugars Reveals a Strategy To Deplete O-Antigens
Source: J Am Chem Soc. 2023 Jul 12;145(29):15639–46. doi: 10.1021/jacs.3c03005 (PMC10375533; doi:10.1021/jacs.3c03005)
Supplement: Supplementary file 1 — ja3c03005_si_001.pdf [file ja3c03005_si_001.pdf]

**Supplemental Information**

# Preference of bacterial rhamnosyltransferases for 6-deoxysugars reveals a strategy to deplete O-antigens

Alexa P. Harnagel,[1] Mia Sheshova,[1] Meng Zheng,[1] Maggie Zheng,[1] Karolina Skorupinska-Tudek,[2] Ewa Swiezewska,[2] Tania J. Lupoli*[1]

[1] Department of Chemistry, New York University, New York, NY 10003, USA
[2] Institute of Biochemistry and Biophysics, Polish Academy of Sciences, Warsaw 02-160, Poland

**TABLE OF CONTENTS**

**Supplemental Figures**

**Table S1.** Percent identities from primary sequence analyses of bacterial rhamnosyltransferases (RTs). 8

**Table S2.** Top twenty hits of putative protein homologs for *T. thermophilus rfbF* using "PRotein Ortholog Search Tool" (PROST)* 8

**Figure S1.** Primary sequence alignments of RTs reveal conservation of a N/DXD motif and H predicted to mediate metal coordination. 9

**Figure S2.** Alignment of predicted protein structures of each RT indicates structural similarity between WbbL and RfbF proteins. 10

**Figure S3.** Titration of 5 mM dTDP into 110 µM RfbF does not result in a measurable binding event. 11

**Figure S4.** Titration of 10 mM L-Rhamnose-1-phosphate into 110 µM RfbF indicates a binding event outside the limit of the instrument. 12

**Figure S5.** Titration of 1 mM dTDP-β-L-Rha into 110 µM RfbF provides a $K_D$ = 8.31 ± 3.66 µM. 13

**Figure S6.** Titration of 750 µM dTDP-6-deoxy-β-L-talose into 110 µM RfbF provides a $K_D$ = 16.5 ± 5.5 µM. 14

**Figure S7.** Titration of 1 mM dTDP-β-L-fucose into 110 µM RfbF provides a $K_D$ = 126 ± 1 µM. 15

**Figure S8.** Titration of 1.1 mM dTDP-β-L-mannose into 110 µM RfbF provides a $K_D$ = 496 ± 56 µM. 16

**Figure S9.** Titration of 2 mM dTDP-α-Glucose into 110 µM WT RfbF provides a $K_D$ = 637 ± 96 µM. 17

**Figure S10.** Titration of 1.5 mM dTDP-α-L-rhamnose into 110 µM RfbF indicates no measurable binding. 18

**Figure S11.** Titration of 1 mM UDP-β-L-rhamnose into 110 µM RfbF provides a $K_D$ = 36.1 ± 10.7 µM. 19

**Figure S12.** Titration of 750 µM GDP-β-L-rhamnose into 110 µM RfbF indicates no measurable binding. 20
**Figure S13.** Titration of 2.5 mM UDP-α-*N*-acetyl-glucosamine into 110 µM RfbF indicates no measurable binding. 21
**Table S3.** N-values calculated from ITC analyses of *T. thermophilus* RfbF with ligands that produced sigmoidal binding curves. 21
**Figure S14** Commercial UDP-Glo Glycosyltransferase Kit (Promega) does not detect changes in concentration of dTDP. 22
**Figure S15.** Standard HPLC traces of byproducts (dT, dTMP, dTDP) of rSAP-treated RT reactions. 22
**Figure S16.** RT reactions are nearly completed within t = 24 hr. 23
**Figure S17.** Initial rates of RTs reveal broad acceptor lipid-length tolerance. 24
**Figure S18.** WbbL/RfbF require glycolipid acceptors for turnover of the native donor. 25
**Figure S19.** Initial rates of RTs reveal tolerance for different dTDP-6-deoxysugar donors. 26
**Table S4.** Specific activities of *E. coli* WbbL, Mtb WbbL, and *T. thermophilus* RfbF in the presence of various donor and acceptor substrates. 27
**Table S5.** High resolution mass spectrometry (ESI) of RT reactions. 28
**Figure S20.** Titration of 5 mM iminosugar (2) into 110 µM RfbF indicates no measurable binding. 29
**Figure S21.** Iminosugar (2) and L-mannose dock differently to the predicted substrate binding pocket of WbbL. 30
**Figure S22.** *E. coli* MG1655 carrying (pBAD33-C-term-FLAG *E. coli wbbL*) express WbbL upon induction. 31
**Figure S23.** Mass of extracted LPS +/- *wbbL* +/- 2 indicates that the amount of LPS is reduced in cells expressing WbbL in the presence of compound 2. 32
**Figure S24.** Biological replicates of cultures grown +/- compound 2 were normalized to contain an equal number of cells in each experiment prior to extraction. 33
**Figure S25.** Iminosugar 2 is not toxic to *E. coli* cells with or without *wbbL*. 34

**Figure S26.** Biological replicates of silver stain and O-16 blots following LPS extraction of *E. coli* cultures +/- 2 (Figure 5C). 35
**Table S6.** Plasmids used in this study 37
**Table S7.** Primers used in this study 38
**Table S8.** Strains used in this study 38

**Supplemental Methods.**
General Methods. 39

**Methods for protein and cellular biochemistry/bioinformatics used in this study.**
Bioinformatic approach to identify *Thermus thermophilus* RfbF as a putative glycolipid RT 39
Cloning of expression vectors for *E. coli* WbbL, Mtb WbbL1, and *T. thermophilus* RfbF overexpression in *E. coli.* 40
Overexpression and purification of *T. thermophilus* RfbF. 40
Overexpression and membrane purification of *E. coli* WbbL and Mtb WbbL. 41
Isothermal Titration Calorimetry (ITC) binding measurements. 41
Specific activity assays for bacterial rhamnosyltransferases from different species. 42
*In vitro* inhibition assay of *E. coli* WbbL with monosaccharides and compound 2. 42
Cellular inhibition of *E. coli* WbbL with compound 2 followed by LPS extraction. 43
Analysis of potential minimum inhibitory concentration of 2 with *E. coli*. 43
Silver staining and western blot visualization of LPS extractions 43
Determination of colony forming units (CFUs) for *E. coli* +/- *wbbL* grown +/- compound 2. 44
Docking of ligands to *E. coli* WbbL model. 44

**Methods for syntheses of compounds used in this study.**
Nucleotide sugars. 45
2-Acetamido-3, 4, 6-tri-*O*-acetyl-2-deoxy-D-glucopyranose (**S1**) 45

2-Acetamido-3,4,6-tri-*O*-acetyl-2-deoxy-α-D-glucopyranose 1-dibenzylphosphate (**S2**) 45
2-Acetamido-3,4,6-tri-*O*-acetyl-2-deoxy-α-D-glucopyranose 1-phosphate (**S3**) 46
2-Acetamido-3,4,6-tri-*O*-acetyl-2-deoxy-α-D-glucopyranose 1-phosphoimidazolide **(S4)** 46
Farnesyl-, geranylgeranyl-, heptaprenyl- and undecaprenyl monophosphates 47
P[1]-2-Acetamido-3,4,6-tri-*O*-acetyl-2-deoxy-α-D-glucopyranosyl P[2] -(2Z,6Z)-3,7,11-trimethyldodeca- 2,6,10-trien-1-yl diphosphate (**S5**) 47
P[1]-2-Acetamido-3,4,6-tri-*O*-acetyl-2-deoxy-α-D-glucopyranosyl P[2]-(2E,6E,10E)-3,7,11,15-tetramethyl hexadeca-2,6,10,14-tetraen-1-yl diphosphate **(S6)** 48
P[1]-2-Acetamido-3,4,6-tri-*O*-acetyl-2-deoxy-α-D-glucopyranosyl P[2] -(2Z,6Z,10Z,14Z,18E,22E)-3,7,11,15,19,23,27-heptamethyloctacosa-2,6,10,14,18,22,26-heptaen-1-yl diphosphate (**S7**) 49
P[1]-2-Acetamido-3,4,6-tri-*O*-acetyl-2-deoxy-α-D-glucopyranosyl P[2] -(2Z,6Z,10Z,14Z,18Z,22Z,26Z,30E,34E,38E)-3,7, 11,15,19,23,27,31,35,39,43 -undecamethyltetratetraconta-2,6,10,14,18,22,26,30,34,38,42-undecaen-1-yl Diphosphate (**S8**) 50
1,5-dideoxy-1,5-imino-L-rhamnitol (**2**) 51
$^{1}$H NMR 2-Acetamido-3, 4, 6-tri-*O*-acetyl-2-deoxy-D-glucopyranose **(S1)** 52
$^{13}$C NMR 2-Acetamido-3, 4, 6-tri-*O*-acetyl-2-deoxy-D-glucopyranose **(S1)** 53
$^{1}$H NMR 2-Acetamido-3,4,6-tri-*O*-acetyl-2-deoxy-α-D-glucopyranose 1-dibenzylphosphate **(S2)** 54
$^{13}$C NMR 2-Acetamido-3,4,6-tri-*O*-acetyl-2-deoxy-α-D-glucopyranose 1-dibenzylphosphate **(S2)** 55
$^{31}$P NMR 2-Acetamido-3,4,6-tri-*O*-acetyl-2-deoxy-α-D-glucopyranose 1-dibenzylphosphate **(S2)** 56
$^{1}$H NMR 2-Acetamido-3,4,6-tri-*O*-acetyl-2-deoxy-α-D-glucopyranose 1-phosphate **(S3)** 57
$^{13}$C NMR 2-Acetamido-3,4,6-tri-*O*-acetyl-2-deoxy-α-D-glucopyranose 1-phosphate **(S3)** 58
$^{31}$P NMR 2-Acetamido-3,4,6-tri-*O*-acetyl-2-deoxy-α-D-glucopyranose 1-phosphate **(S3)** 59
$^{31}$P NMR 2-Acetamido-3,4,6-tri-*O*-acetyl-2-deoxy-α-D-glucopyranose 1-phosphoimidazolide **(S4)** 60

$^{1}H$ NMR $P^1$-2-Acetamido-3,4,6-tri-*O*-acetyl-2-deoxy-α-D-glucopyranosyl $P^2$ -(2Z,6Z)-3,7,11-trimethyldodeca- 2,6,10-trien-1-yl diphosphate **(S5)** 61

$^{13}C$ NMR $P^1$-2-Acetamido-3,4,6-tri-*O*-acetyl-2-deoxy-α-D-glucopyranosyl $P^2$ -(2Z,6 Z)-3,7,11-trimethyldodeca- 2,6,10-trien-1-yl diphosphate **(S5)** 62

$^{31}P$ NMR $P^1$-2-Acetamido-3,4,6-tri-*O*-acetyl-2-deoxy-α-D-glucopyranosyl $P^2$ -(2Z,6Z)-3,7,11-trimethyldodeca- 2,6,10-trien-1-yl diphosphate **(S5)** 63

$^{1}H$ NMR $P^1$-2-Acetamido-3,4,6-tri-*O*-acetyl-2-deoxy-α-D-glucopyranosyl $P^2$-(2E,6E,10E)-3,7,11,15-tetramethyl hexadeca-2,6,10,14-tetraen-1-yl diphosphate **(S6)** 64

$^{13}C$ NMR $P^1$-2-Acetamido-3,4,6-tri-*O*-acetyl-2-deoxy-α-D-glucopyranosyl $P^2$-(2E,6E,10E)-3,7,11,15-tetramethyl hexadeca-2,6,10,14-tetraen-1-yl diphosphate **(S6)** 65

$^{31}P$ NMR $P^1$-2-Acetamido-3,4,6-tri-*O*-acetyl-2-deoxy-α-D-glucopyranosyl $P^2$-(2E,6E,10E)-3,7,11,15-tetramethyl hexadeca-2,6,10,14-tetraen-1-yl diphosphate **(S6)** 66

$^{1}H$ NMR $P^1$-2-Acetamido-3,4,6-tri-*O*-acetyl-2-deoxy-α-D-glucopyranosyl $P^2$ -(2Z,6Z,10Z,14Z,18E,22E)-3,7,11,15,19,23,27-heptamethyloctacosa-2,6,10,14,18,22,26-heptaen-1-yl diphosphate **(S7)** 67

$^{13}C$ NMR $P^1$-2-Acetamido-3,4,6-tri-*O*-acetyl-2-deoxy-α-D-glucopyranosyl $P^2$ -(2Z,6Z,10Z,14Z,18E,22E)-3,7,11,15,19,23,27-heptamethyloctacosa-2,6,10,14,18,22,26-heptaen-1-yl diphosphate **(S7)** 68

$^{31}P$ NMR $P^1$-2-Acetamido-3,4,6-tri-*O*-acetyl-2-deoxy-α-D-glucopyranosyl $P^2$ -(2Z,6Z,10Z,14Z,18E,22E)-3,7,11,15,19,23,27-heptamethyloctacosa-2,6,10,14,18,22,26-heptaen-1-yl diphosphate **(S7)** 69

$^{1}H$ NMR $P^1$-2-Acetamido-3,4,6-tri-*O*-acetyl-2-deoxy-α-D-glucopyranosyl $P^2$ -(2Z,6Z,10Z,14Z,18Z,22Z,26Z,30E,34E,38E)-3,7, 11,15,19,23,27,31,35,39,43 -undecamethyltetratetraconta-2,6,10,14,18,22,26,30,34,38,42-undecaen-1-yl Diphosphate **(S8)** 70

$^{13}C$ NMR $P^1$-2-Acetamido-3,4,6-tri-*O*-acetyl-2-deoxy-α-D-glucopyranosyl $P^2$ -(2Z,6Z,10Z,14Z,18Z,22Z,26Z,30E,34E,38E)-3,7, 11,15,19,23,27,31,35,39,43 -undecamethyltetratetraconta-2,6,10,14,18,22,26,30,34,38,42-undecaen-1-yl Diphosphate **(S8)** 71

$^{31}$P NMR P$^{1}$-2-Acetamido-3,4,6-tri-*O*-acetyl-2-deoxy-α-D-glucopyranosyl P$^{2}$ -(2Z,6Z,10Z,14Z,18Z,22Z,26Z,30E,34E,38E)-3,7, 11,15,19,23,27,31,35,39,43 -undecamethyltetratetraconta-2,6,10,14,18,22,26,30,34,38,42-undecaen-1-yl Diphosphate **(S8)** 72

$^{1}$H NMR 1-5-dideoxy-1,5-imino-L-rhamnitol **(2)** 73

$^{13}$C NMR 1-5-dideoxy-1,5-imino-L-rhamnitol **(2)** 74

**References** 75

**Table S1. Percent identities from primary sequence analyses of bacterial rhamnosyl-transferases (RTs).***

| Protein** (Uniprot entry) | *S. flexneri* RfbF (P37782) | *T. thermophilus* RfbF (Q72KB9) | *E. coli* WbbL (P36667) | Mtb WbbL1 (P9WMY3) |
|---|---|---|---|---|
| *S. flexneri* RfbF | 100.00 | 20.61 | 19.01 | 21.01 |
| *T. thermophilus* RfbF | 20.61 | 100.00 | 14.75 | 28.10 |
| *E. coli* WbbL | 19.01 | 14.75 | 100.00 | 28.48 |
| Mtb WbbL1 | 21.01 | 28.10 | 25.48 | 100.00 |

* Alignments generated with Clustal Omega.[1] Please see the "Supplemental Methods" section for more details.
** *Shigella flexneri*, *Thermus thermophilus*, *Escherichia coli*, *Mycobacterium tuberculosis* (Mtb)

**Table S2. Top twenty hits of putative protein homologs for *T. thermophilus rfbF* using "PRotein Ortholog Search Tool" (PROST[2]).***

| Uniprot Entry | **Organism (strain)** (*wbbL/rfbF* genes highlighted in red) | Gene | e-value** |
|---|---|---|---|
| A0R5Z2 | *Mycolicibacterium smegmatis* (strain ATCC 700084) | *glfT1* | 3.39E-44 |
| P9WMX2 | *Mycobacterium tuberculosis* (strain CDC 1551) | *glfT1* | 1.27E-40 |
| P9WMX3 | *Mycobacterium tuberculosis* (strain ATCC 25618) | *glfT1* | 1.27E-40 |
| P37782 | *Shigella flexneri* | *rfbF* | 1.29E-32 |
| D4GU63 | *Haloferax volcanii* (strain ATCC 29605) | *agl10* | 4.48E-22 |
| P9WMY2 | *Mycobacterium tuberculosis* (strain CDC 1551) | *wbbL* | 8.22E-22 |
| P9WMY3 | *Mycobacterium tuberculosis* (strain ATCC 25618) | *wbbL* | 8.22E-22 |
| B3VA59 | *Methanococcus voltae* | *aglC* | 9.85E-18 |
| P33695 | *Rhizobium meliloti* (strain 1021) | *exoM* | 1.88E-17 |
| D4GYH3 | *Haloferax volcanii* (strain ATCC 29605) | *aglG* | 2.49E-16 |
| P26403 | *Salmonella typhimurium* (strain ATCC 700720) | *rfbN* | 5.19E-16 |
| D4GYG7 | *Haloferax volcanii* (strain ATCC 29605) | *aglE* | 3.54E-14 |
| D4GYH2 | *Haloferax volcanii* (strain ATCC 29605) | *aglI* | 1.30E-13 |
| P9WLV3 | *Mycobacterium tuberculosis* (strain ATCC 25618) | *wbbL2* | 3.79E-12 |
| P64868 | *Mycobacterium bovis* (strain ATCC BAA-935) | *wbbL2* | 3.79E-12 |
| P9WLV2 | *Mycobacterium tuberculosis* (strain CDC 1551) | *wbbL2* | 3.79E-12 |
| P33691 | *Rhizobium meliloti* (strain 1021) | *exoA* | 1.98E-11 |
| P36667 | *Escherichia coli* (strain K12) | *wbbL* | 3.61E-11 |
| P47271 | *Mycoplasma genitalium* (strain ATCC 33530) | MG025 | 1.72E-10 |
| P75086 | *Mycoplasma pneumoniae* (strain ATCC 29342) | MPN_028 | 2.23E-09 |

*The following url was used for the PROST search: https://mesihk.github.io/prost
**Note that an e-value cutoff of 0.001 is expected to yield 1/5 hits with no structural similarity. All of the indicated hits are far below this cutoff.

```
S.      MNSNIYAVIVTYNPELKN--LNALITELKEQNCYVVVVDNRT-NFTLKDKLADIEKVHLI    57
T.      MSERVCAVIVTYNRKALLRECLKAVLSQTRPPDHVLVVDNASTDGTPEMLQEEFPQVEVL    60
E.      ---MVYIIIVSHGHEDYIKKLLENLNADDE-HYKIIVRDNKD-SLLLKQICQHYAGLDYI    55
M.      ------VVAVTYSPGPHLERFLASLSLATERPVSVLLADNGSTDGTPQAAVQRYPNVRLL    54
               : *::.           :    .    ::: **   .   :        :  :

S.      CLGRNE----GIAKAQNIGIRYSLE--------KGAEKIIFFDQDSRIRNEFIKKLSCYM    105
T.      RLPENQGGAGGFHEGMKRA-----------YE-QGVDWLWLMDDDTIPKAKALEALLEAA    108
E.      -SGGVY----GFGHNNNIAVAYVK----EKYRPADDDYILFLNPDIIMKHDDLLTYIKYV    106
M.      PTGANL----GYGTAVNRTIAQLGEMAGDAGEPWVDDWVIVANPDVQWGPGSIDALLDAA    110
                  *     :                   : : . : *       :

S.      D-NENAKIAGPVFIDRDKSHYYPICNIKKNGLREKIHVTEG----QTPFKS---------    151
T.      RL--------PL--DPRPRV-LASRQLLPNGLPHPTTAFVNPTDPRHPFL----WLRLRP    153
E.      ESKRYAFSTLCLFRDEAKSLHDYSVRKFPVLSDFIVSFMLGINKTKIP-------KESIY    159
M.      SRWPRAGALGPLIRDPDGSV-YPSARQMPSLIRGGMHAVLGPFWPRNPWTTAYRQERLEP    169
                   :  *          .              .    : *

S.      ---SV--TISSGTMVSKEVFEIVGMMDEELFIDYVDTEWCLRCLNYGILVHIIPDIKMVH    206
T.      RYRPIRWALFTSVLLHRSLVEEHGLPHKAFFIWEDDLEYTARALRRGLGLQ-VRDSEVIH    212
E.      SDTVVDWCAGSFMLVRFSDFVRVNGFDQGYFMYCEDIDLCLRLSLAGVRLHYVPAFHAIH    219
M.      SERPVGWLSGSCLLVRRSAFGQVGGFDERYFMYMEDVDLGDRLGKAGWLSVYVPSAEVLH    229
            :     :  ::  . .   .  .:  *:   * :   *    *     :   . :*

S.      AIGDKSVKICGINIPI-HSPVRRYYRVRNAFLLLRKNHVPLLLS--IREVVFSLIHTTLI    263
T.      KSASKP------YISATTGENRLFYGVRNRIWVLRSPAFGPLGKAFLALQLLFGLLTYLA    266
E.      YAHHDN------------------------RSFFSKAFRWHLKST--FRYLARKRILSNRN    254
M.      HKAHSTGRDPASHLAAHHKSTYIFLADRHSGWWRAPLRWTLRGS--LALRSHLMVRSSLR    287
            .                                      .  :       : :

S.      IATQKNKIEYMKKHILATLDGIRGITGGGRYNA       296
T.      FHPSQKSLQEIGRALRAGLTTSPC---------       290
E.      FD--RISSVFHP---------------------       264
M.      RSRRRKLKLVEGRH-------------------       301
            :
```

**Figure S1. Primary sequence alignments of RTs reveal conservation of a N/DXD motif and H predicted to mediate metal coordination.** Predicated conserved metal coordinating residues are boxed in red. The following sequences are shown: *S. flexneri* RfbF (S), *T. thermophilus* RfbF (T), *E. coli* WbbL (E), Mtb WbbL (M). Alignments were performed using Clustal Omega.[1]

A)

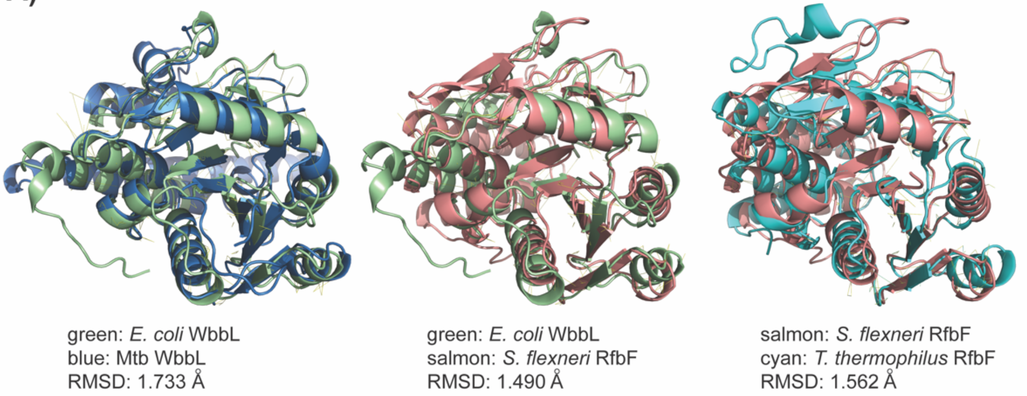


B)

| Protein model | average pLDDT |
|---|---|
| *E. coli* WbbL | 91.85 |
| Mtb WbbL | 89.50 |
| *S. flexneri* RfbF | 92.08 |
| *T. thermophilus* RfbF | 90.75 |

**Figure S2. Alignment of predicted protein models of each RT indicates structural similarity between WbbL and RfbF proteins. (A)** Alignments of indicated proteins performed using PyMOL (The PyMOL Molecular Graphics System, Version 2.0 Schrödinger, LLC.). Because there are no solved protein structures of WbbL or RfbF, the AlphaFold Monomer v2.0 pipeline was used to model each protein of interest.[3-4] Indicated RMSD values (root-mean-square deviation) of aligned atoms were based on C-alpha backbone alignment. For comparison, <3 Å RMSD is typical for alignment of homologous protein structures.[5-6] (B) Average per-residue confidence scores (pLDDTs) for indicated AlphaFold structures indicates at least "Confident" model results (90 > pLDDT > 70).

**A)**

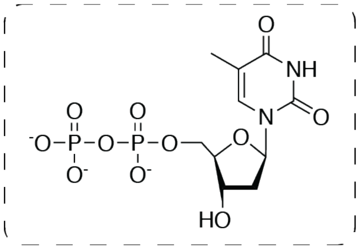

**B)**

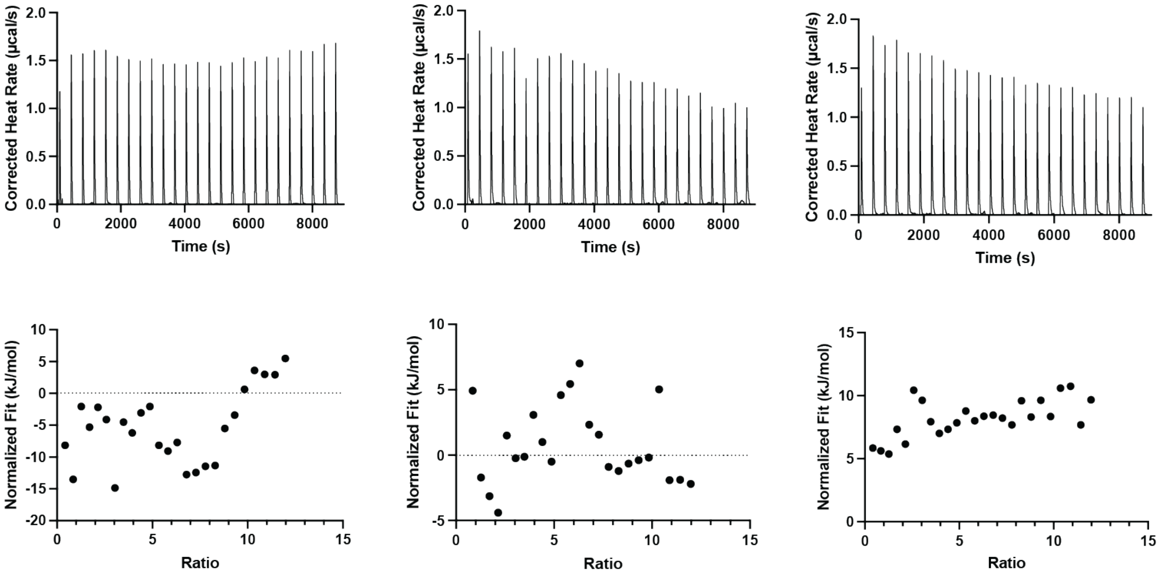


**Figure S3. Titration of 5 mM dTDP into 110 µM RfbF does not result in a measurable binding event by isothermal titration calorimetry (ITC) analysis.** A) Structure of dTDP. B) Triplicate binding curves (top) of dTDP into 110 µM RfbF and normalized fits (bottom). A control experiment (5 mM dTDP into buffer) is subtracted from the raw data profiles to obtain normalized fits (bottom). Under these conditions, no binding profile is observed. For all ITC data, ratio indicates (ligand:RT) ratio.

**A)**

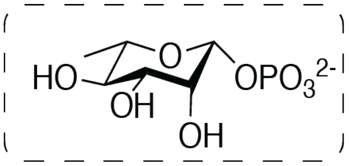


**B)**

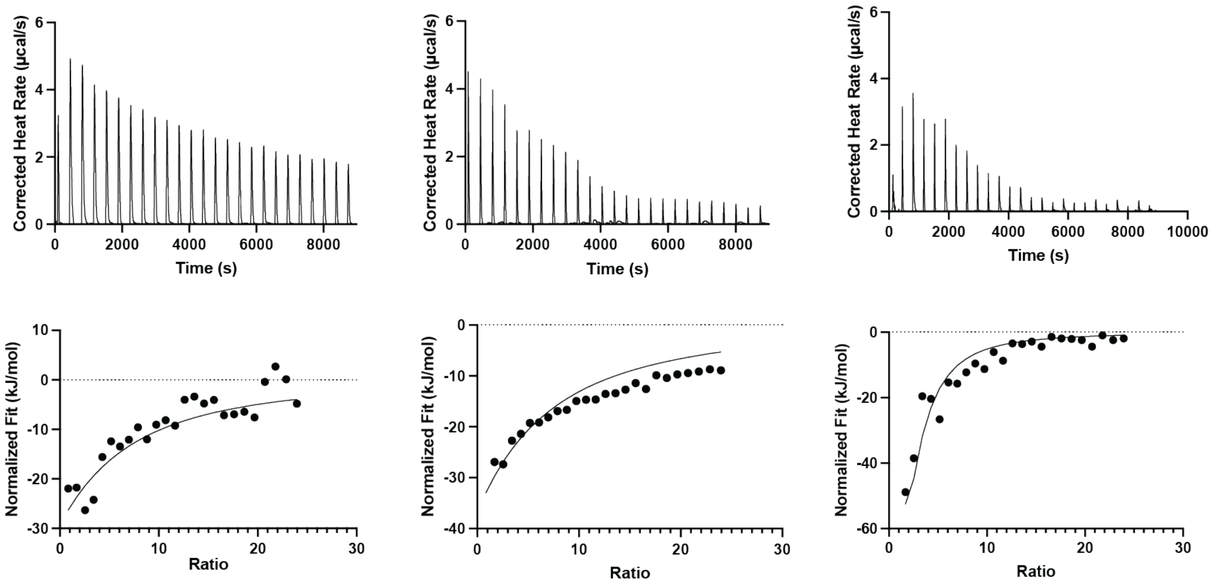


**Figure S4. Titration of 10 mM L-Rhamnose-1-phosphate into 110 µM RfbF indicates a binding event outside the limit of the instrument.** A) Structure of L-Rha-1-phosphate (L-Rha-1-phosphate); B) Triplicate binding curves (top) of L-Rha-1-phosphate into 110 µM RfbF and normalized fits (bottom). A control experiment (10 mM L-Rha-1-phosphate into buffer) is subtracted from the raw data profiles to obtain normalized fits (bottom). Under these conditions, a binding profile is observed beyond the limit that can be measured by the instrument $K_D$ (>1 mM). As the binding affinity decreases, the sigmoidal profile is reduced to a hyperbolic profile, which can only provide estimations of ΔH, ΔS, and ΔG.

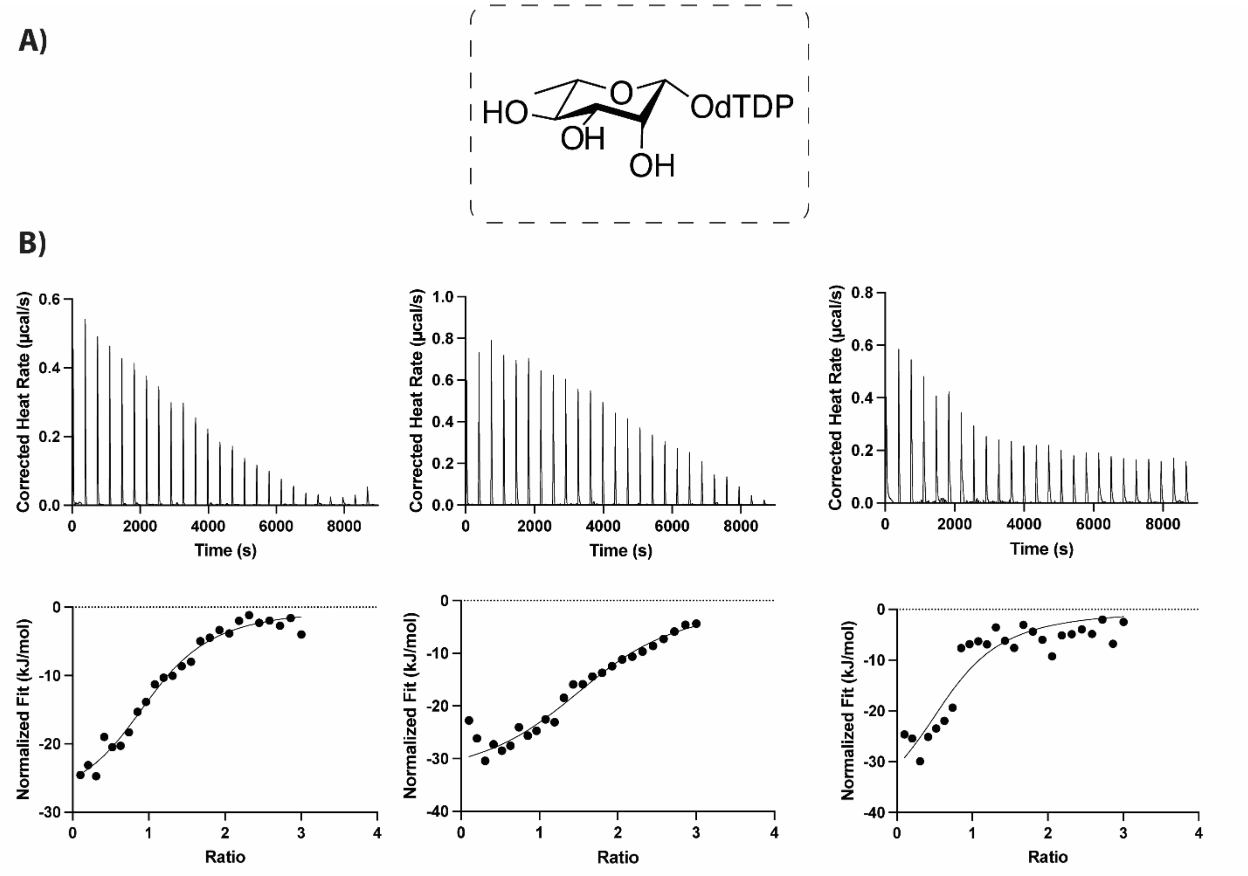


**Figure S5. Titration of 1 mM dTDP-β-L-Rha into 110 µM RfbF provides a $K_D$ = 8.31 ± 3.66 µM.** A) Structure dTDP-β-L-Rha; B) Triplicate binding curves (top) of dTDP-β-L-Rha into 110 µM RfbF and normalized fits (bottom). A control experiment (1 mM dTDP-β-L-Rha into buffer) is subtracted from the raw data profiles to obtain normalized fits (bottom), producing a sigmoidal binding curve.

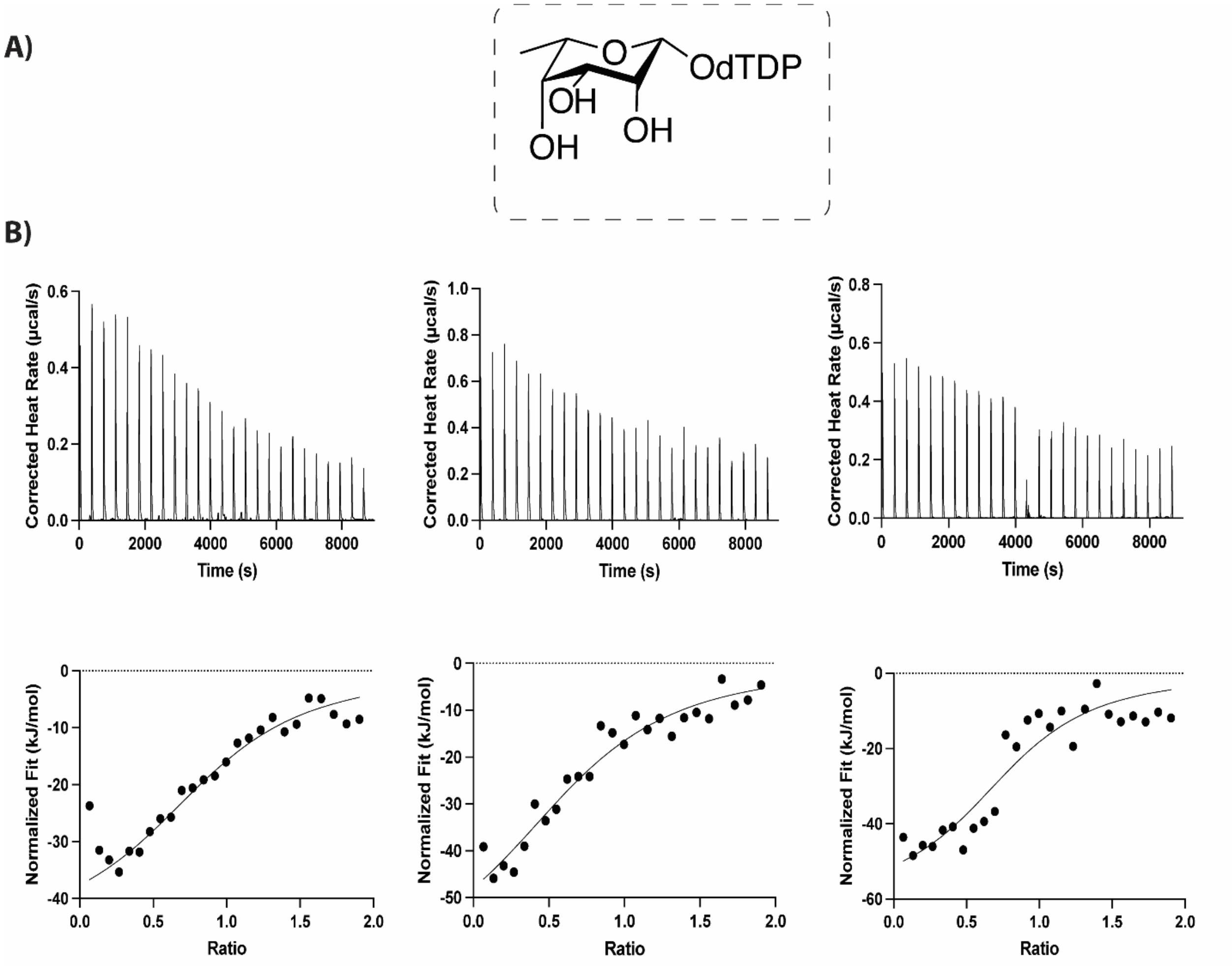


**Figure S6. Titration of 750 µM dTDP-6-deoxy-β-L-talose into 110 µM RfbF provides a $K_D$ = 16.5 ± 5.5 µM.** A) Structure dTDP-β-L-6dTal; (B) Triplicate binding curves (top) of 750 µM dTDP-β-L-6dTal into 110 µM RfbF and normalized fits (bottom). A control experiment (750 µM dTDP-β-L-6dTal into buffer) is subtracted from the raw data profiles to obtain normalized fits (bottom), producing a sigmoidal binding curve.

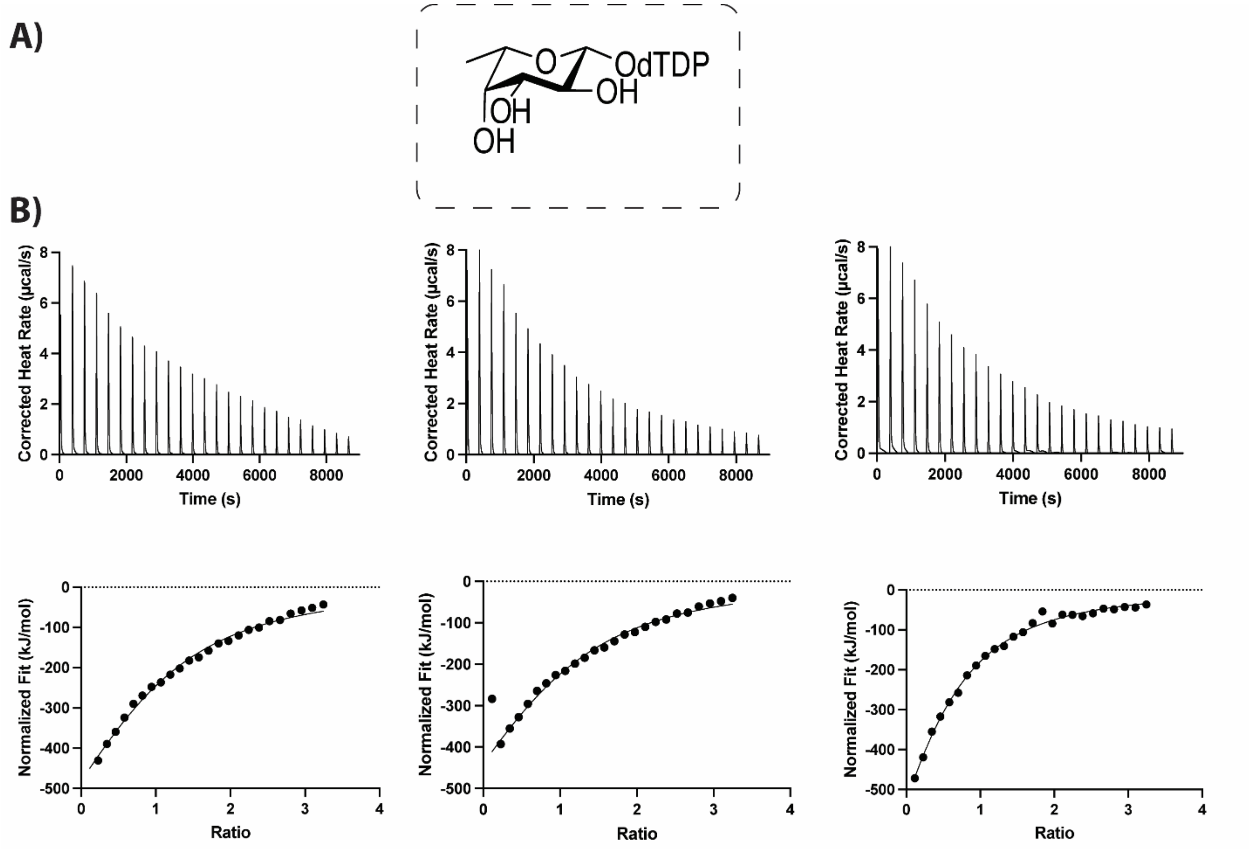


**Figure S7. Titration of 1 mM dTDP-β-L-fucose into 110 μM RfbF provides a $K_D$ = 126 ± 1 μM.** A) Structure dTDP-β-L-fucose (dTDP-β-L-Fuc); B) Triplicate binding curves (top) of 1 mM dTDP-β-L-Fuc into 110 μM RfbF and normalized fits (bottom). A control experiment (1 mM dTDP-β-L-Fuc into buffer) is subtracted from the raw data profiles to obtain normalized fits (bottom), producing a hyperbolic binding curve. Due to the lack of saturation obtained, reliable thermodynamic parameters cannot be reported.

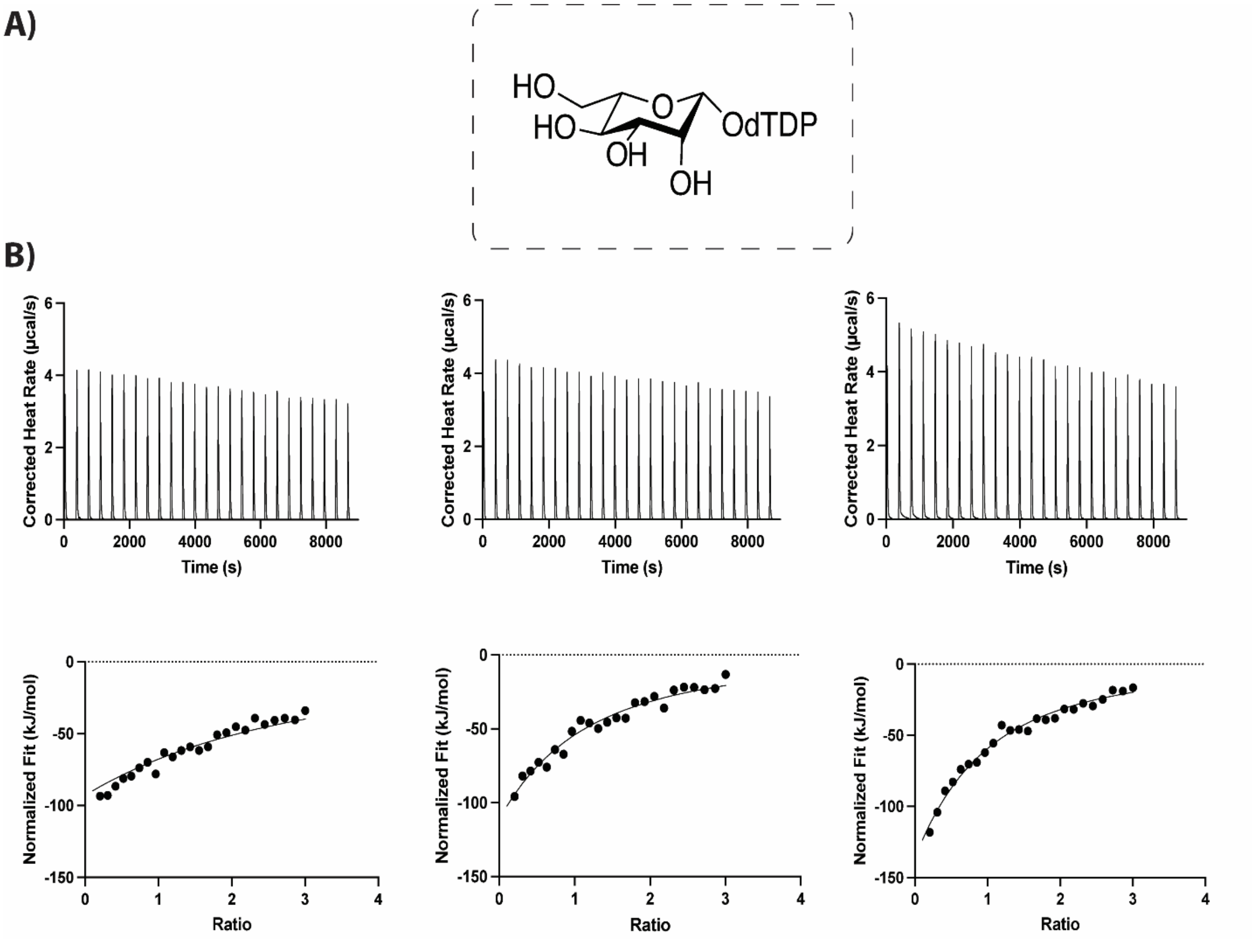


**Figure S8. Titration of 1.1 mM dTDP-β-L-mannose into 110 µM RfbF provides a $K_D$ = 496 ± 56 µM.** A) Structure dTDP-β-L-Man; B) Triplicate binding curves (top) of 1.1 mM dTDP-β-L-Man into 110 µM RfbF and normalized fits (bottom). A control experiment (1.1 mM dTDP-Man into buffer) is subtracted from the raw data profiles to obtain normalized fits (bottom), producing a hyperbolic binding curve. Due to the lack of saturation obtained, reliable thermodynamic parameters cannot be reported.

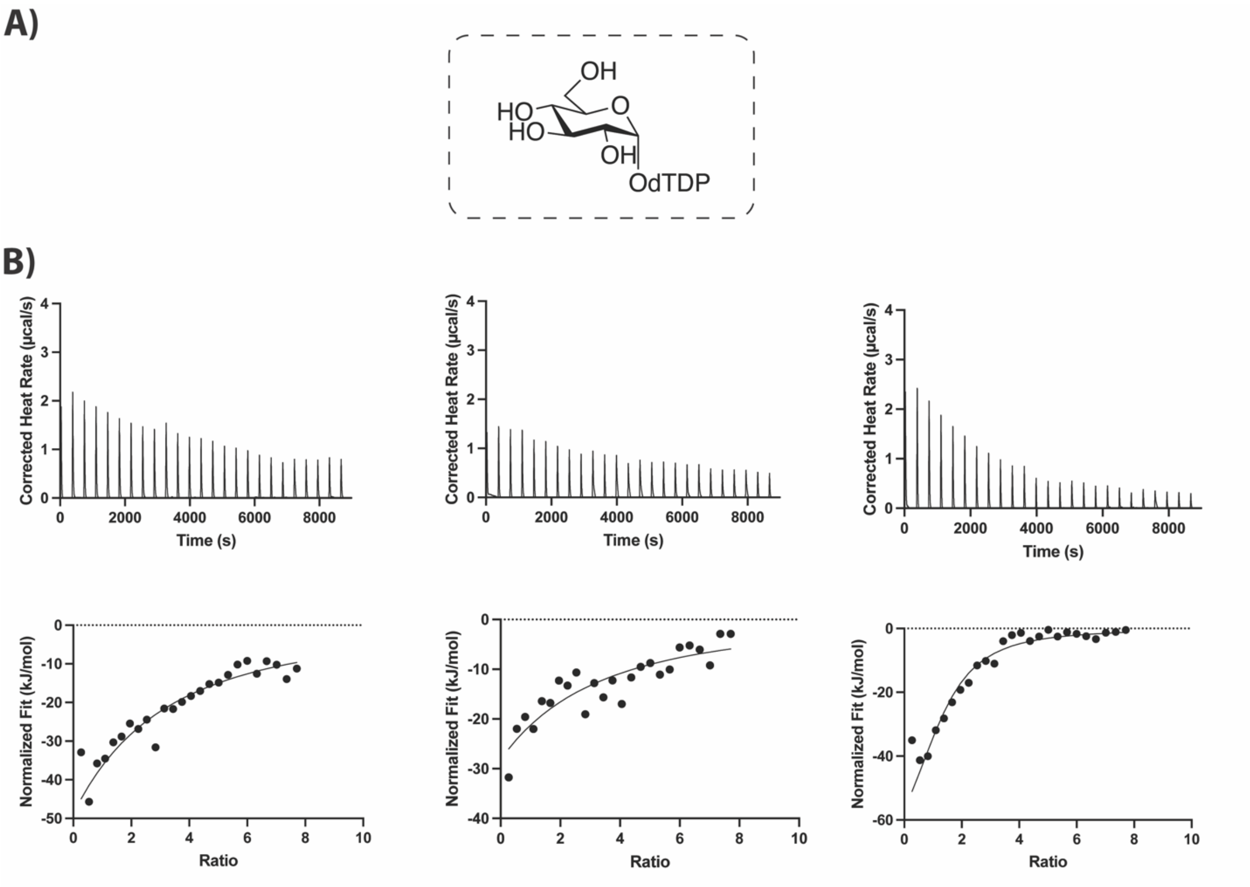


**Figure S9. Titration of 2 mM dTDP-$\alpha$-D-glucose into 110 µM RfbF provides a $K_D$ = 637 ± 96 µM.** A) Structure of dTDP-$\alpha$-Glc; B) Triplicate binding curves (top) of 2 mM dTDP-$\alpha$-Glc into 110 µM RfbF and normalized fits (bottom). A control experiment (2 mM dTDP-$\alpha$-Glc into buffer) is subtracted from the raw data profiles. Due to the lack of saturation obtained, reliable thermodynamic parameters cannot be reported.

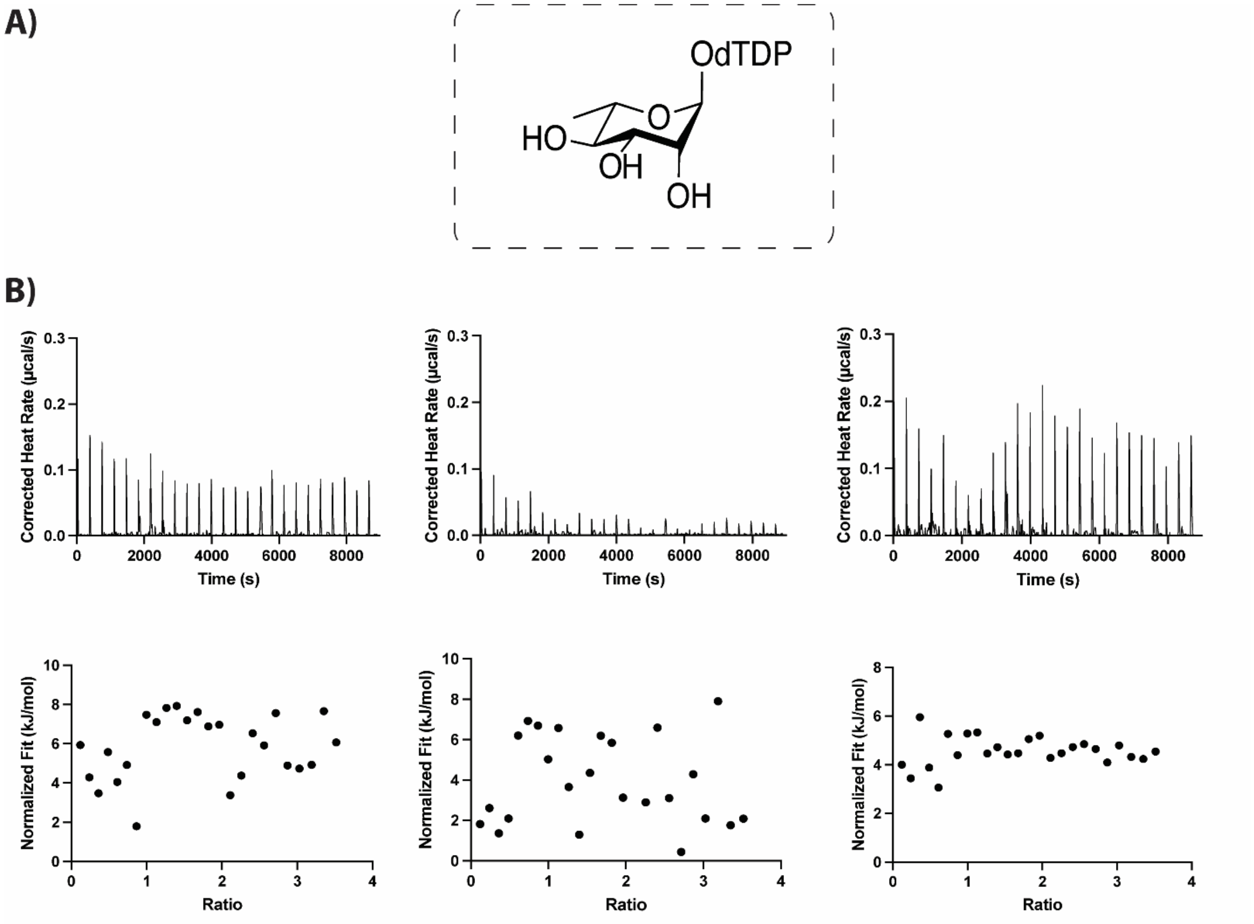


**Figure S10. Titration of 1.5 mM dTDP-α-L-rhamnose into 110 µM RfbF indicates no measurable binding.** A) Structure of dTDP-α-L-Rha; B) Triplicate binding curves (top) of 1.5 mM dTDP-α-L-Rha into 110 µM RfbF and normalized fits (bottom). A control experiment (1.5 mM dTDP-α-L-Rha into buffer) is subtracted from the raw data profiles.

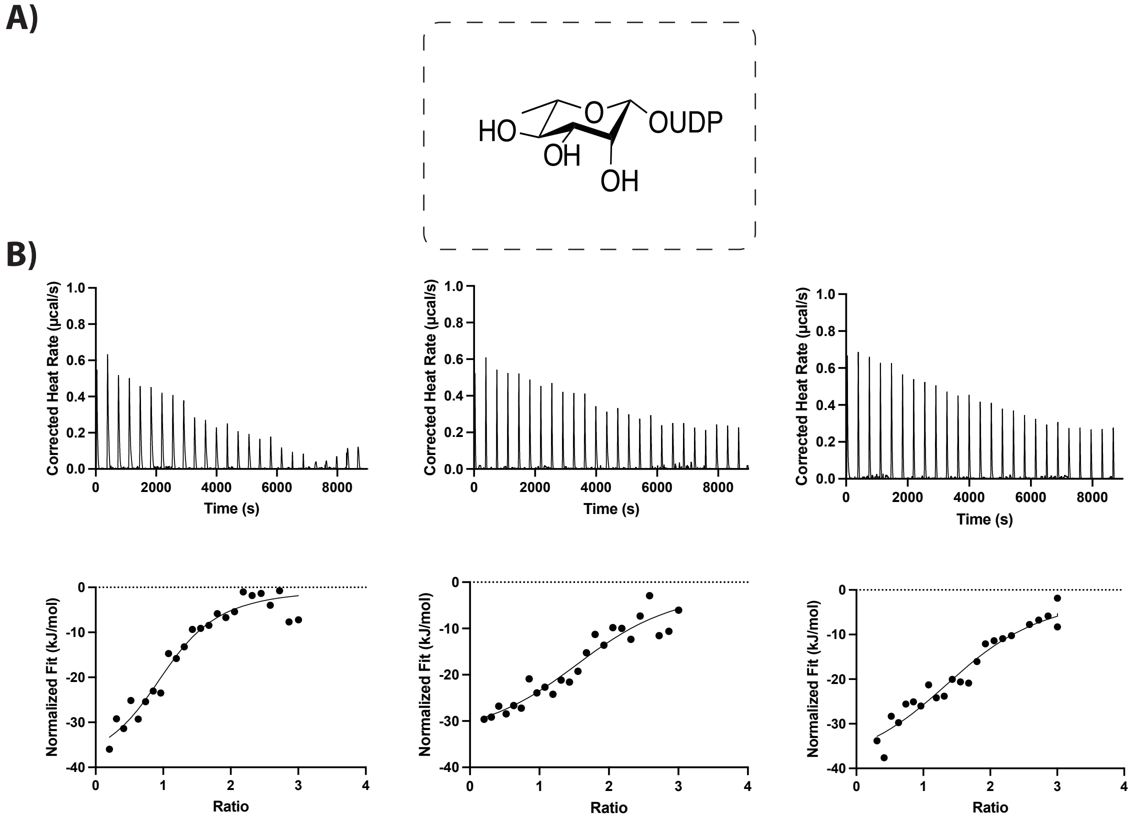


**Figure S11. Titration of 1 mM UDP-β-L-rhamnose into 110 µM RfbF provides a $K_D$ = 36.1 ± 10.7 µM.** A) Structure UDP-β-L-Rha; B) Triplicate binding curves (top) of 1 mM UDP-β-L-Rha into 110 µM RfbF and normalized fits (bottom). A control experiment (1 mM UDP-β-L-Rha into buffer) is subtracted from the raw data profiles to obtain normalized fits (bottom) to obtain a sigmoidal binding curve.

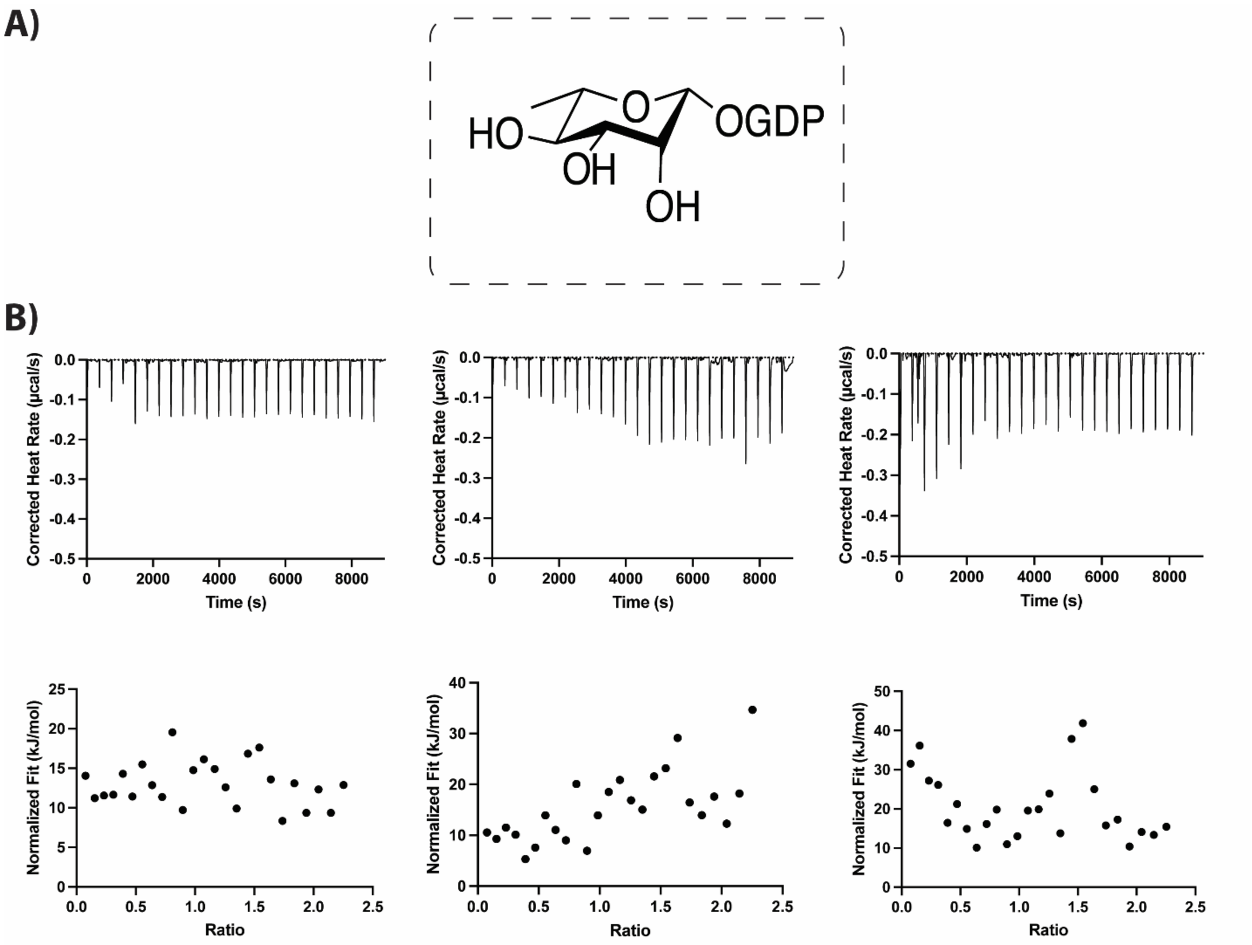


**Figure S12. Titration of 750 µM GDP-β-L-rhamnose into 110 µM RfbF indicates no measurable binding.** A) Structure of GDP-β-L-Rha; B) Triplicate binding curves (top) of 750 µM GDP-β-L-Rha into 110 µM RfbF and normalized fit (bottom). A control experiment (750 µM GDP-β-L-Rha into buffer) is subtracted from the raw data profiles to obtain normalized fits (bottom).

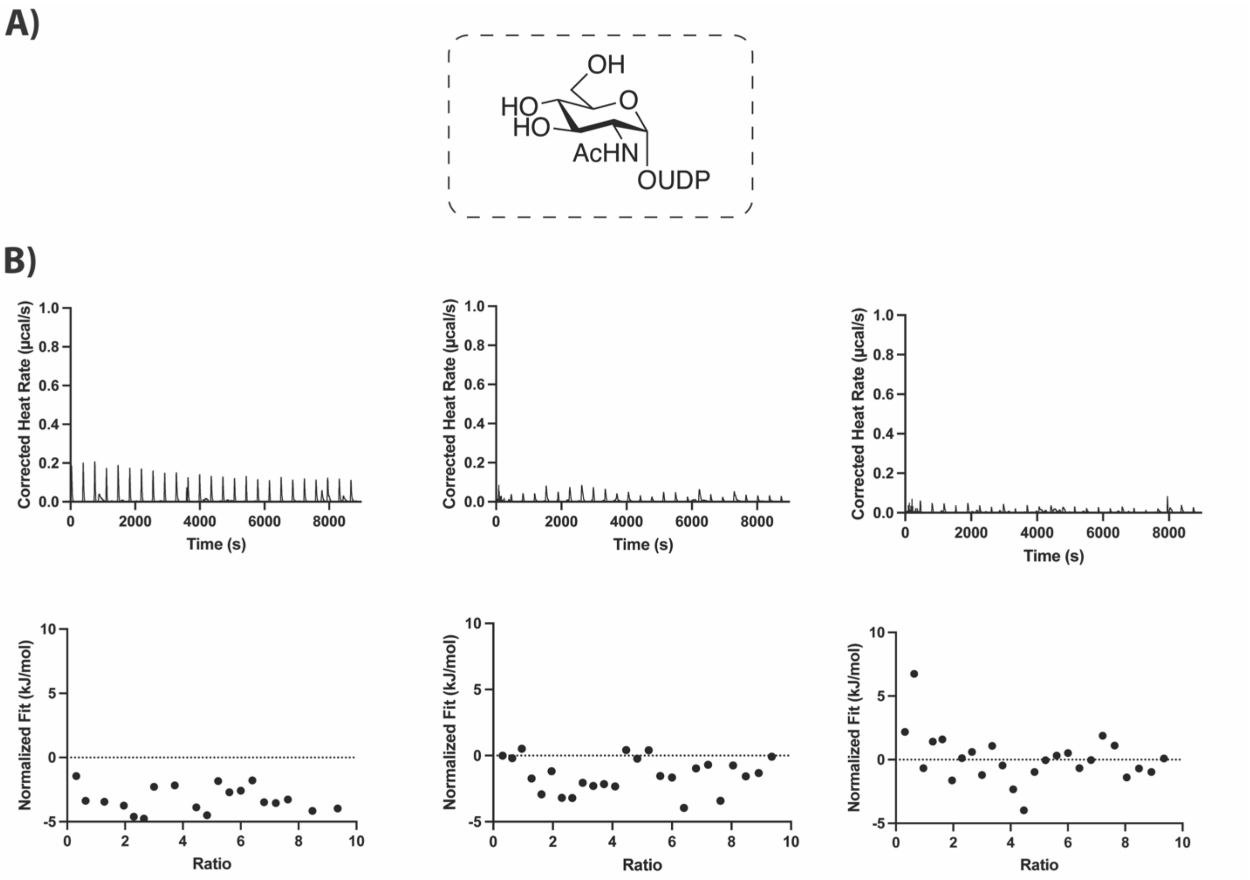


**Figure S13. Titration of 2.5 mM UDP-α-*N*-acetyl-D-glucosamine into 110 µM RfbF indicates no measurable binding.** A) Structure of UDP-α-D-GlcNAc; B) Triplicate binding curves (top) of 2.5 mM UDP-α-D-GlcNAc into 110 µM RfbF and normalized fit (bottom). A control experiment (2.5 mM UDP-α-D-GlcNAc into buffer) is subtracted from the raw data profiles to obtain normalized fits (bottom).

**Table S3. N-values calculated from ITC analyses of *T. thermophilus* RfbF with ligands that produced sigmoidal binding curves.**

| (d)NDP-sugar | n-value* |
|---|---|
| dTDP-β-L-Rha | 1.10 (0.03) |
| dTDP-β-L-6dTal | 0.934 (0.028) |
| UDP-β-L-Rha | 1.30 (0.28) |

*Parentheses indicate standard deviation (SD) for n = 3 experiments.

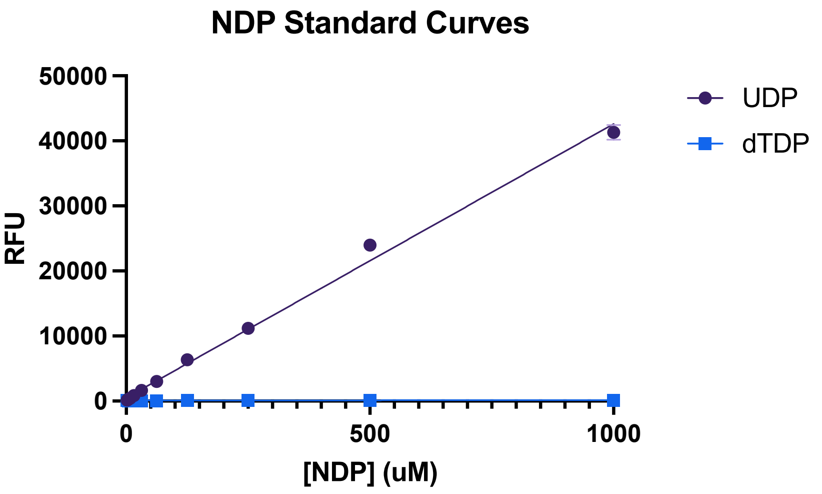


**Figure S14. Commercial UDP-Glo Glycosyltransferase Assay (Promega) does not detect changes in concentration of dTDP.** UDP-Glo reagents are sensitive to changes in the concentration of UDP (dark purple, 1000 μM to 1.95 μM), which results in a linear standard curve ($R^2$ = 0.99). UDP-Glo reagents are not sensitive to changes in concentration of dTDP (blue, 1000 μM to 1.95 μM). Error bars represent standard deviation (SD) (n = 2).

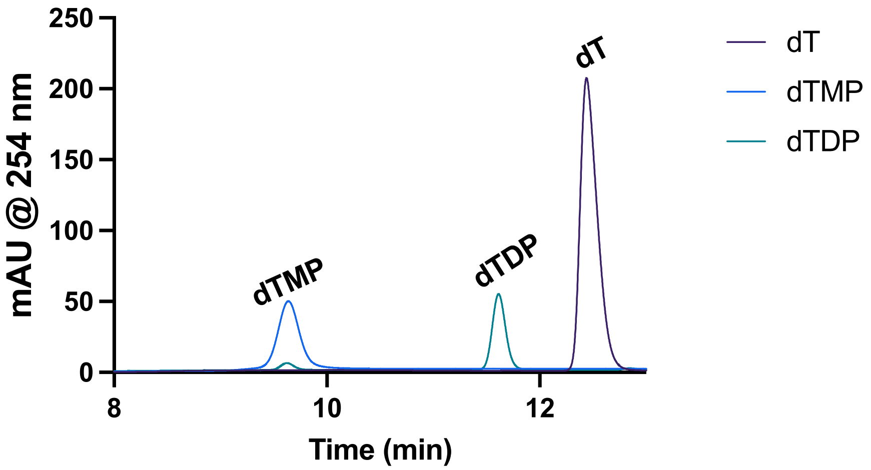


**Figure S15. Standard HPLC traces of byproducts (dT, dTMP, dTDP) of rSAP-treated RT reactions.** 15 μL of each (dTDP, dTMP or dT) was analyzed by reverse phase HPLC equipped with a Phenomenex 5 μm, 150 x 21.2 mm, NX-C18 110 Å Gemini column with solvent A (50 mM triethylammonium bicarbonate (TEAB), pH 8.0) and solvent B (acetonitrile) using the following linear gradient: 0% B (t = 0-5 min), 0% to 5% B (t = 5-25 min), 5-90% B (t = 25-35 min). Absorbance at wavelength $\lambda$ = 254 nm was monitored. dTDP is the native leaving group of the donor (dTDP-β-L-Rha); dTDP is digested by shrimp alkaline phosphatase (rSAP) into dTMP and dT in RT assays.

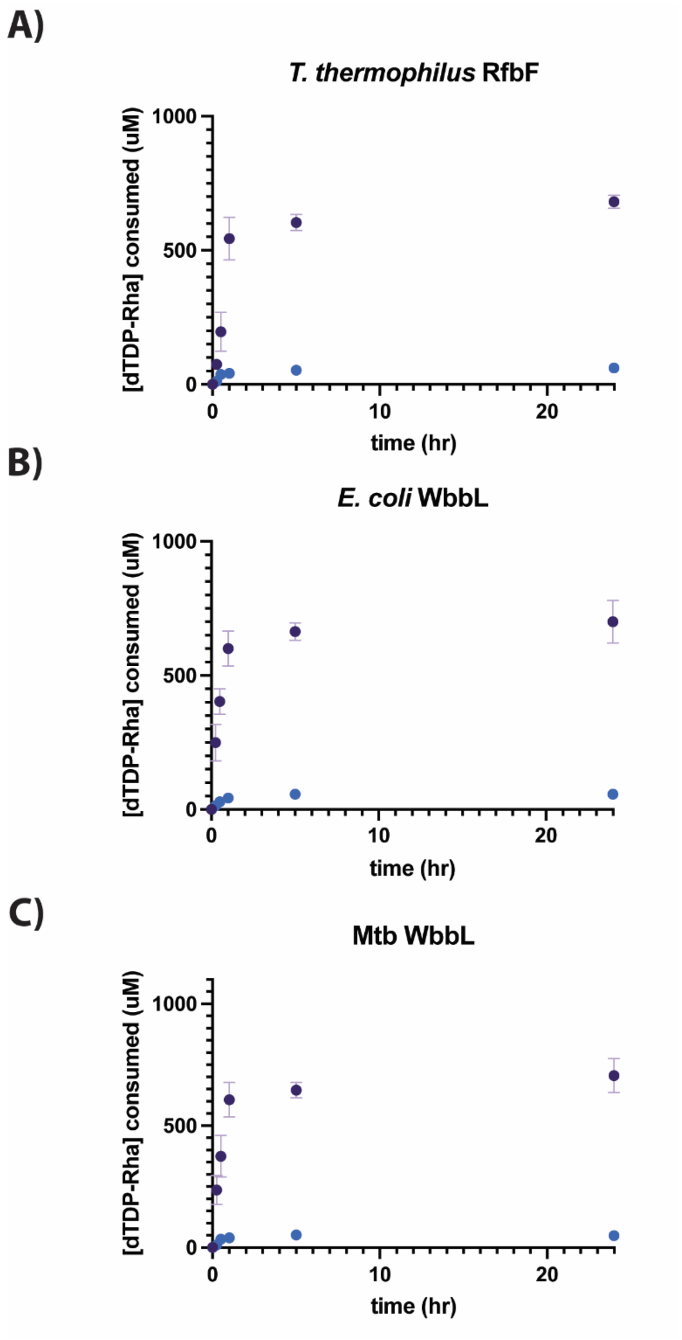


**Figure S16. RT reactions are nearly completed within t = 24 hr.** Reactions containing the following RTs: (A) RfbF (0.155 mg/mL), (B) *E. coli* WbbL (0.081 mg/mL), and (C) Mtb WbbL (0.111 mg/mL). 1 mM dTDP-β-L-Rha and 1 mM GlcNAc-PP-C55 (dark purple) were added to reactions; identical reactions with 5 mM EDTA added (blue). Consumption of dTDP-β-L-Rha was calculated by subtracting concentration of dTDP-β-L-Rha at t = 0. Thymidine containing compounds present in the initial reaction (dTMP, dTDP) were also background subtracted using t = 0. Reactions were incubated as described in Methods. Error bars represent SD (n = 3).

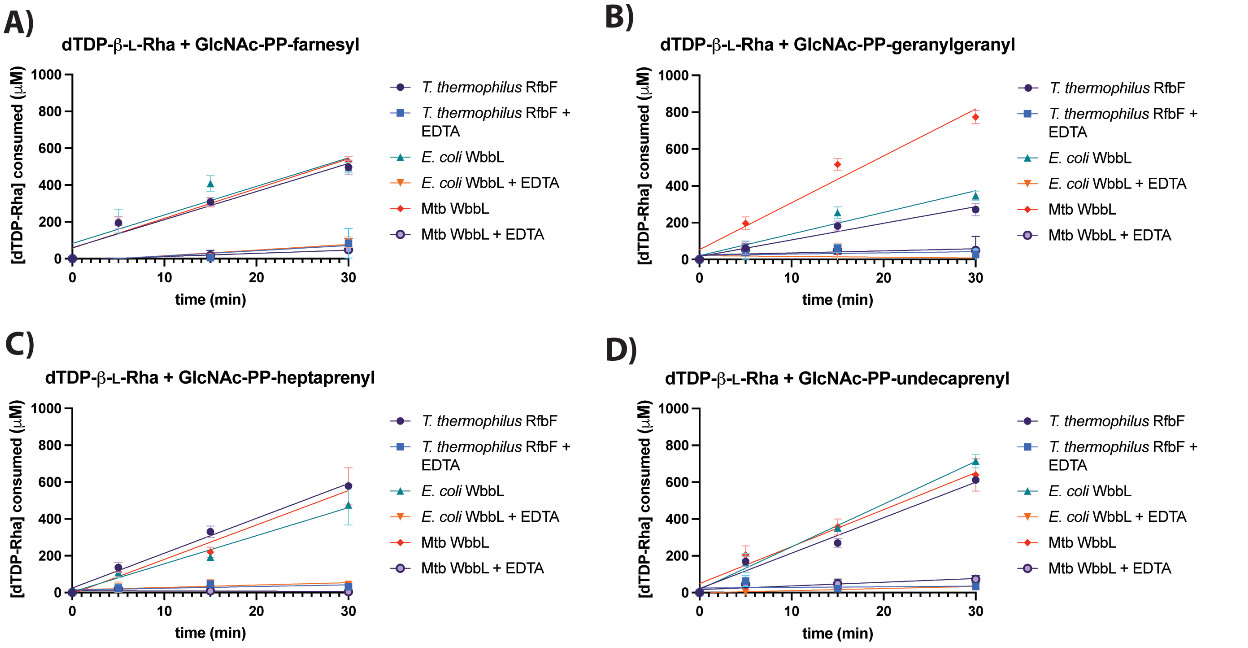


**Figure S17. Initial rates of RTs reveal broad acceptor lipid-length tolerance**. Initial rate analysis of *T. thermophilus* RfbF (0.155 mg/mL), *E. coli* WbbL (0.081 mg/mL), and Mtb WbbL (0.111 mg/mL) in the presence of 1 mM dTDP-β-L-Rha and 1 mM of one of the following as the acceptor: A) GlcNAc-PP-farnesyl, B) GlcNAc-PP-geranylgeranyl, C) GlcNAc-PP-heptaprenyl, and D) GlcNAc-PP-undecaprenyl. Reactions in the presence of 5 mM EDTA serve as a control and indicate basal hydrolysis of nucleotide-sugar donor. Both Mtb and *E. coli* WbbL were analyzed as membrane preparations from *E. coli*, but endogenous lipid did not appear to affect their utilization of different lipid lengths. Error bars represent SD (n = 9).

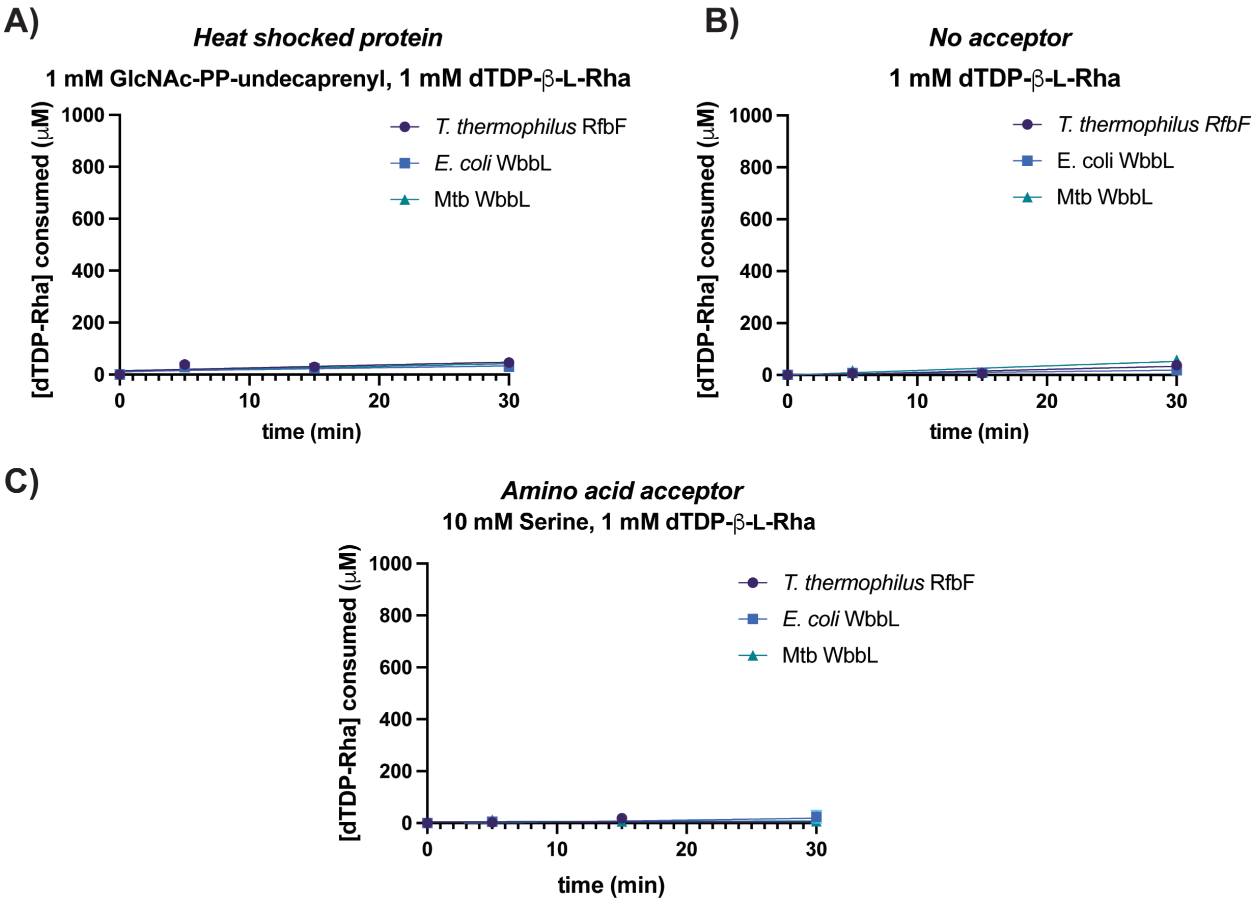


**Figure S18. WbbL/RfbF require glycolipid acceptors for turnover of the native donor.** Initial rate analysis of indicated RTs (A) with glycolipid acceptor and native donor following heat treatment (105 °C, t = 5 min); (B) with no acceptor added and the native donor; (C) with an amino acid acceptor that contains a free alcohol and the native donor. Besides the indicated changes, reactions were carried out as described in **Figure S17**.

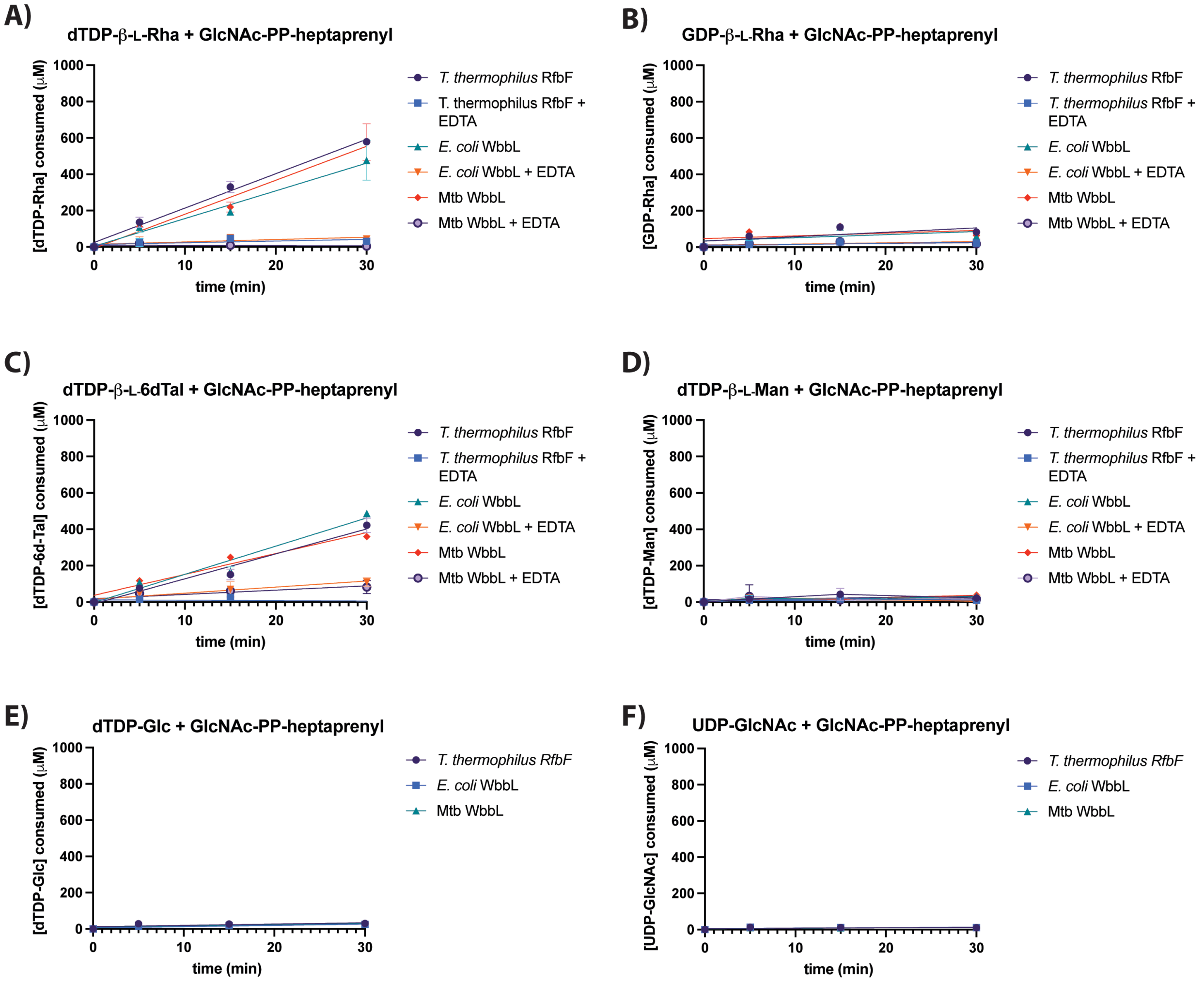


**Figure S19. Initial rates of RTs reveal utilization of canonical and non-canonical dTDP-6-deoxysugar donors**. Initial rate analysis of *T. thermophilus* RfbF (0.155 mg/mL), *E. coli* WbbL (0.081 mg/mL), and Mtb WbbL (0.111 mg/mL) in the presence of 1 mM GlcNAc-PP-C35 and 1 mM of one of the following possible donors: A) dTDP-β-L-Rha;, B) GDP-β-L-Rha, C) dTDP-β-L-6dTal, D) dTDP-β-L-Man, E) dTDP-α-D-Glc, and F) UDP-α-D-GlcNAc. Many of the corresponding reactions are also shown in the presence of 5 mM EDTA. Error bars represent SD (n = 3-9).

**Table S4. Specific activities of *E. coli* WbbL, Mtb WbbL, and *T. thermophilus* RfbF in the presence of various donor and acceptor substrates.***

| | ***E. coli* WbbL** | **Mtb WbbL** | ***T. thermophilus* RfbF** |
|---|---|---|---|
| **Acceptor** | $\mu$M min$^{-1}$ mg$^{-1}$ | $\mu$M min$^{-1}$ mg$^{-1}$ | $\mu$M min$^{-1}$ mg$^{-1}$ |
| GlcNAc-PP-C15 | 1.46x10$^{4}$ | 1.86x10$^{4}$ | 9.53x10$^{3}$ |
| | (1.80x10$^{3}$) | (1.45X10$^{3}$) | (3.72x10$^{2}$) |
| GlcNAc-PP-C20 | 2.29x10$^{4}$ | 1.41x10$^{4}$ | 5.64 x10$^{3}$ |
| | (8.15x10$^{2}$) | (1.07x10$^{3}$) | (2.98x10$^{2}$) |
| GlcNAc-PP-C35 | 1.68x10$^{4}$ | 1.82x10$^{4}$ | 1.18x10$^{4}$ |
| | (8.04x10$^{2}$) | (1.54x10$^{3}$) | (2.69x10$^{2}$) |
| GlcNAc-PP-C55 | 1.81x10$^{4}$ | 2.78 x10$^{4}$ | 1.20x10$^{4}$ |
| | (8.61x10$^{2}$) | (6.32x10$^{2}$) | (3.66x10$^{2}$) |
| **Donor** | $\mu$M min$^{-1}$ mg$^{-1}$ | $\mu$M min$^{-1}$ mg$^{-1}$ | $\mu$M min$^{-1}$ mg$^{-1}$ |
| dTDP-β-L-Rha | 1.68x10$^{4}$ | 1.82x10$^{4}$ | 1.18x10$^{4}$ |
| | (8.04x10$^{2}$) | (1.54x10$^{3}$) | (2.69x10$^{2}$) |
| dTDP-β-L-6dTal | 1.04x10$^{4}$ | 1.85x10$^{4}$ | 8.56x10$^{3}$ |
| | (4.35x10$^{2}$) | (7.03x10$^{2}$) | (3.34x10$^{2}$) |
| dTDP-β-L-Man | 1.34x10$^{2}$ | 9.18x10$^{2}$ | 4.45x10$^{2}$ |
| | (4.39x10$^{2}$) | (2.55x10$^{2)}$ | (1.94x10$^{2}$) |
| GDP-β-L-Rha | 4.16x10$^{2}$ | 2.04x10$^{3}$ | 1.52x10$^{3}$ |
| | (1.80x10$^{2}$) | (6.16x10$^{2}$) | (2.79x10$^{2}$) |
| dTDP-α-D-Glc | 8.51x10$^{2}$ | 6.74x10$^{2}$ | 4.50x10$^{2}$ |
| | (3.95x10$^{2}$) | (1.84x10$^{2}$) | (1.63x10$^{2}$) |
| UDP- α-D-GlcNAc | 2.89x10$^{2}$ | 2.72x10$^{2}$ | 1.27x10$^{2}$ |
| | (1.52x10$^{2}$) | (8.77x10$^{1}$) | (8.17x10$^{1}$) |

* n = 9, parenthesis represent standard deviation. Data is calculated from curves shown in **Figures S17 and S19**.

**Table S5. High resolution mass spectrometry (ESI) of RT reactions.**

| product | Calculated $[M-H]^-$ | Observed $[M-H]^-$ |
|---|---|---|
| | ***E. coli*** **WbbL** | |
| α-L-Rha-(1→3)-α-D-GlcNAc-PP-C55 | 1274.7618 | 1274.7659 |
| α-L-Rha-(1→3)-α-D-GlcNAc-PP-C35 | 1002.5114 | 1002.5186 |
| α-L-Rha-(1→3)-α-D-GlcNAc-PP-C20 | 798.3236 | 798.3227 |
| α-L-Rha-(1→3)-α-D-GlcNAc-PP-C15 | 364.6269 | 364.6279 |
| α-L-6dTal-(1→3)-α-D-GlcNAc-PP-C35 | 1002.5114 | 1002.5140 |
| | **Mtb WbbL** | |
| α-L-Rha-(1→3)-α-D-GlcNAc-PP-C55 | 1274.7618 | 1274.7652 |
| α-L-Rha-(1→3)-α-D-GlcNAc-PP-C35 | 1002.5114 | 1002.5198 |
| α-L-Rha-(1→3)-α-D-GlcNAc-PP-C20 | 798.3236 | 798.3236 |
| α-L-Rha-(1→3)-α-D-GlcNAc-PP-C15 | 730.2610 | 730.2612 |
| α-L-6dTal-(1→3)-α-D-GlcNAc-PP-C35 | 500.7521 | 500.7520 |
| | ***T. thermophilus*** **RfbF** | |
| α-L-Rha-(1→3)-α-D-GlcNAc-PP-C55 | 1274.7618 | 1274.7612 |
| α-L-Rha-(1→3)-α-D-GlcNAc-PP-C35 | 1002.5114 | 1002.5035 |
| α-L-Rha-(1→3)-α-D-GlcNAc-PP-C20 | 798.3236 | 798.3225 |
| α-L-Rha-(1→3)-α-D-GlcNAc-PP-C15 | 730.2610 | 730.2624 |
| α-L-6dTal-(1→3)-α-D-GlcNAc-PP-C35 | 1002.5114 | 1002.5093 |

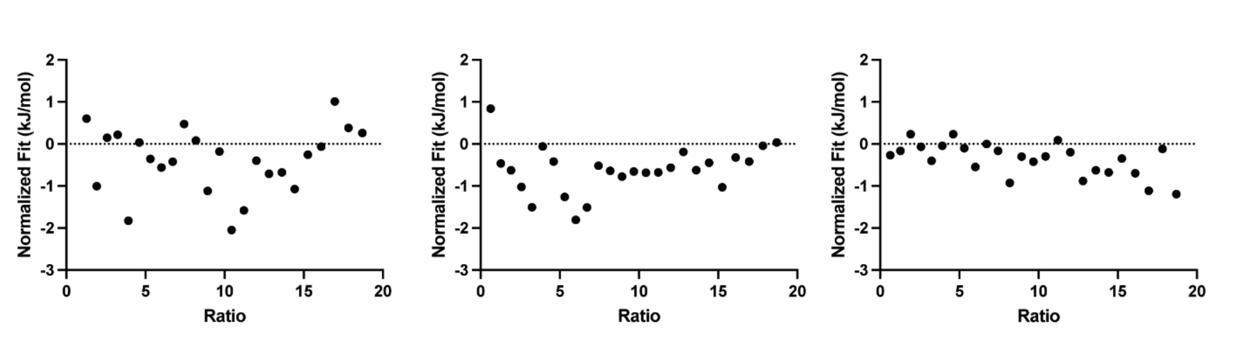


**Figure S20. Titration of 5 mM iminosugar (2) into 110 µM RfbF indicates no measurable binding.** Normalized fits of 5 mM **2** into 110 µM RfbF. A control experiment (5 mM **2** into buffer) is subtracted from the raw data profiles to obtain normalized fits.

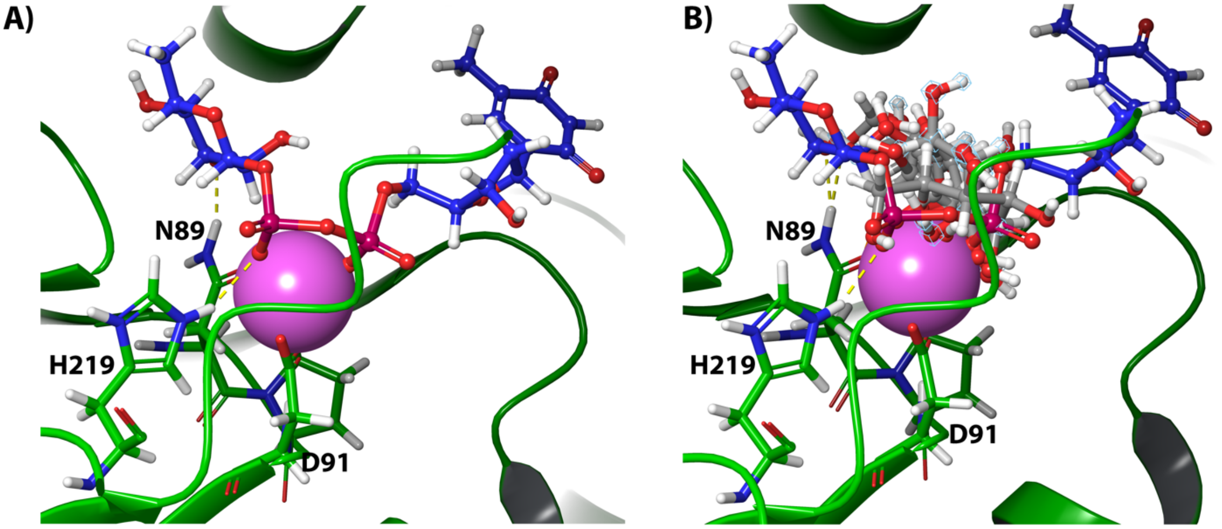


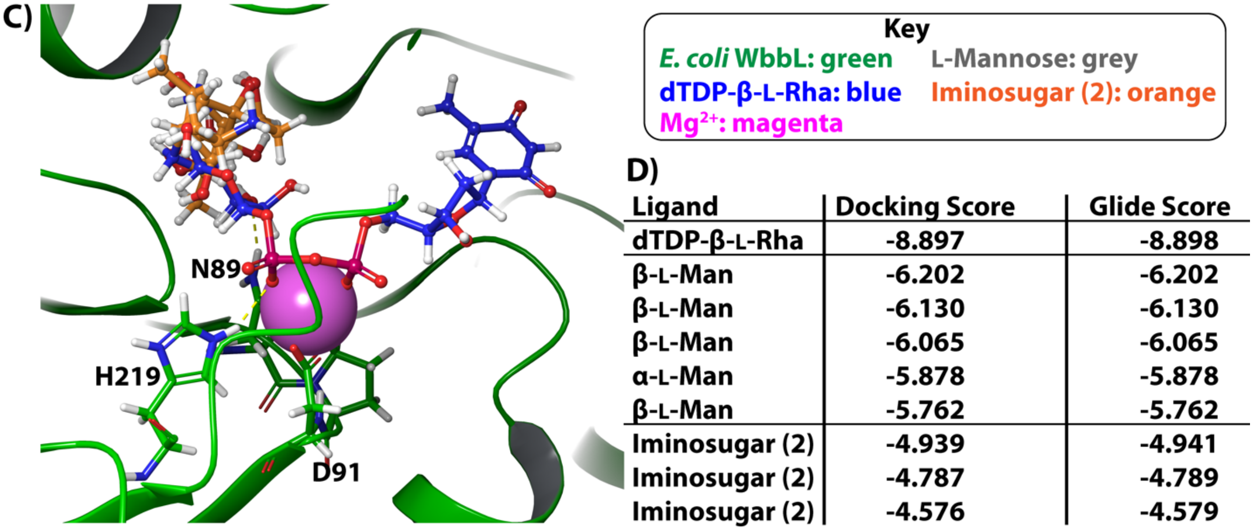


D)

| Ligand | Docking Score | Glide Score |
|---|---|---|
| dTDP-β-L-Rha | -8.897 | -8.898 |
| β-L-Man | -6.202 | -6.202 |
| β-L-Man | -6.130 | -6.130 |
| β-L-Man | -6.065 | -6.065 |
| α-L-Man | -5.878 | -5.878 |
| β-L-Man | -5.762 | -5.762 |
| Iminosugar (2) | -4.939 | -4.941 |
| Iminosugar (2) | -4.787 | -4.789 |
| Iminosugar (2) | -4.576 | -4.579 |

**Figure S21. Iminosugar (2) and L-Mannose dock differently to the predicted substrate binding pocket of WbbL.** (A) dTDP-β-L-Rha (blue) docked into the AlphaFold predicted model of *E. coli* WbbL (showing the NXD motif and conserved H) using Schrodinger Glide.[3-4] This pose shows interactions between the phosphates and $Mg^{2+}$ that would likely occur in the active site. (B) Five poses of L-Man docked into predicted substrate binding pocket with the lowest docking scores; all associate with the $Mg^{2+}$ ion. (C) Three poses of iminosugar (**2**) docked into predicted substrate binding pocket with the lowest docking scores indicate that iminosugar binds deeper in the binding pocket than L-Man, and overlaps with the L-Rha moiety in the docked WbbL-dTDP-β-L-Rha complex. (D) Table with predicted docking and ligand scores (units of kcal/mol) for parts A-C. See the Supplemental Methods section for more details.

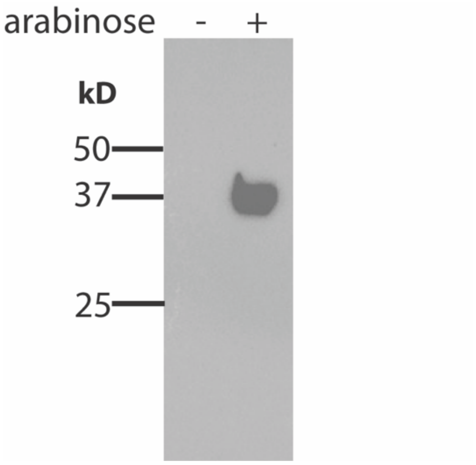


**Figure S22. *E. coli* MG1655 carrying (pBAD33-C-term-FLAG *E. coli wbbL*) express WbbL upon induction with arabinose.** Indicated strain was grown in LB (chloramphenicol 30 μg/mL) to log-phase at 25 °C prior to induction with 1.33 mM arabinose for t = 3 hr (+ sample). Sample was also collected pre-induction (- sample). For each sample, 5 mL of cells were pelleted prior to sonication (5 min, 30 sec on, 30 sec off, 50% amplitude), and resuspension in 6x laemmli buffer prior to analysis by SDS-PAGE. Samples were transferred to PVDF membrane prior to blotting with 1:2,000 anti-FLAG-HRP and imaging with ECL reagent (Bio-Rad). Predicted molecular weight ~32 kD.

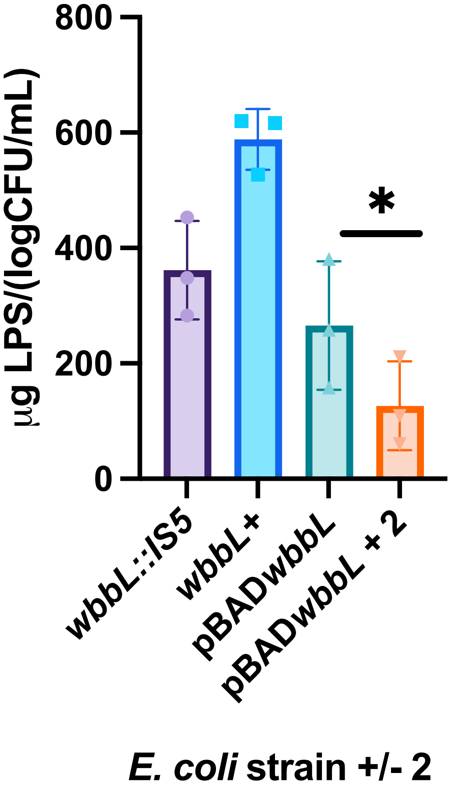


**Figure S23. Mass of extracted LPS +/- *wbbL* +/- 2 indicates that the amount of LPS is reduced in cells expressing WbbL in the presence of compound 2.** Number of cells per treated or untreated culture were normalized (**Figure S24**) prior to LPS extraction and mass of recovered LPS was recorded. Mass of LPS (μg) per number of cells/mL (colony forming units/mL or CFU/mL) in sample is reduced when cells express WbbL in the presence of compound **2 (~2 mM)** (compare green to orange bars). These results suggest that LPS has less attached O-antigen. The purple bar serves as a negative control, in which the strain used (*E. coli* MG16155) has a *wbbL::*IS5 insertion, which results in loss of function and no O-antigen.[7] The blue bar represents the positive control, in which the former strain has a functional copy of *wbbL* added and the resulting *E. coli* (*wbbL*+) produces O-antigen. The green and orange bars represent the MG1655 strain (*wbbL::*IS5 insertion) containing pAPH343 (pBAD33-C-term-FLAG *E. coli* WbbL), where WbbL is expressed upon addition of arabinose (1.33 mM). Bars indicate SD (n = 3). * p<0.0332. Paired t-test was used for comparison.

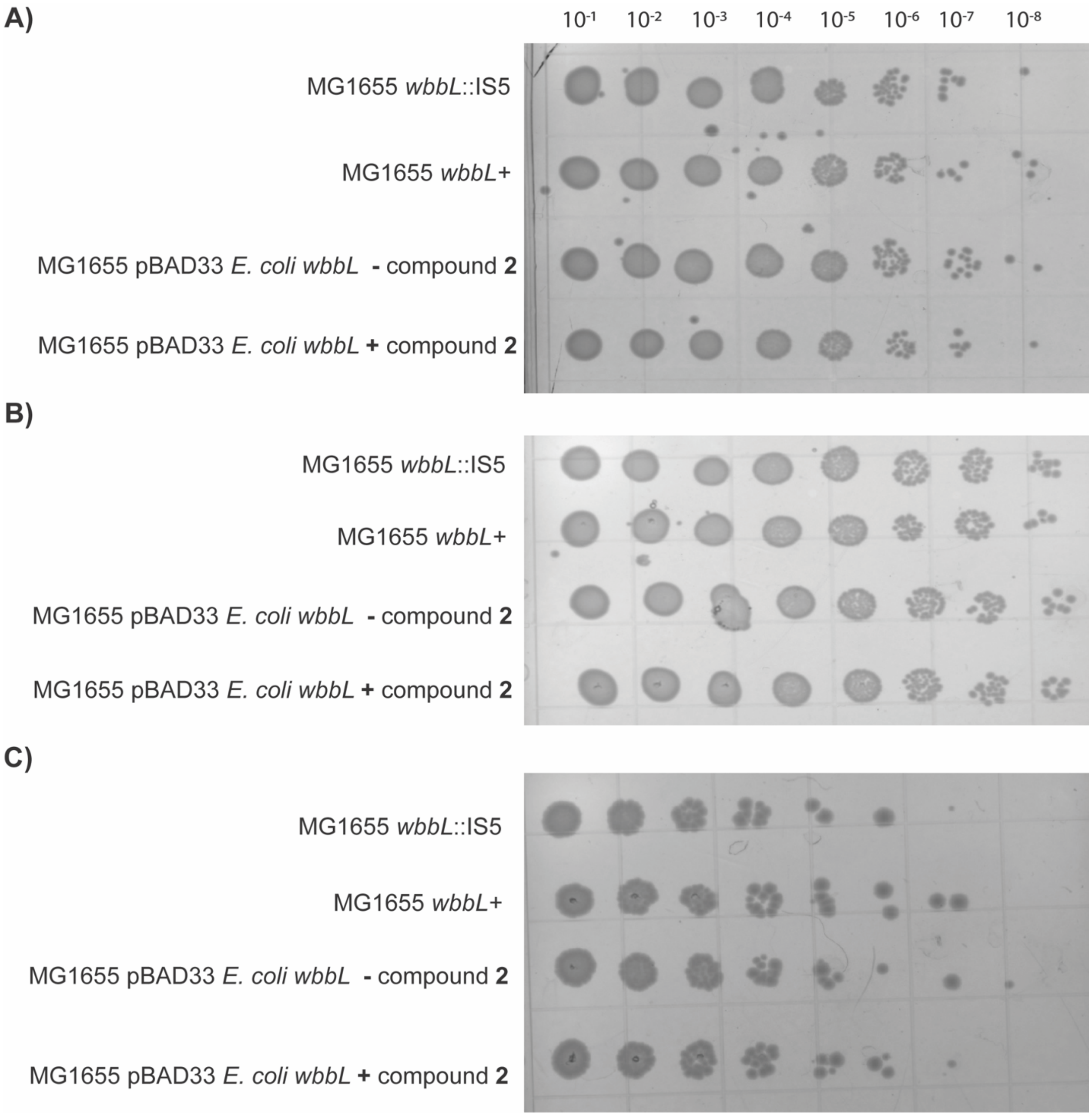


**Figure S24. Biological replicates of cultures grown +/- compound 2 were normalized to contain an equal number of cells in each experiment prior to extraction.** Cultures were normalized to an equal optical density at wavelength 600 nm ($OD_{600}$), serial diluted, plated, and incubated overnight at 37 °C. Colonies were counted the following day. Number of cells were approximately the same per sample across replicates (~8.26 x $10^{10}$ CFU/mL).

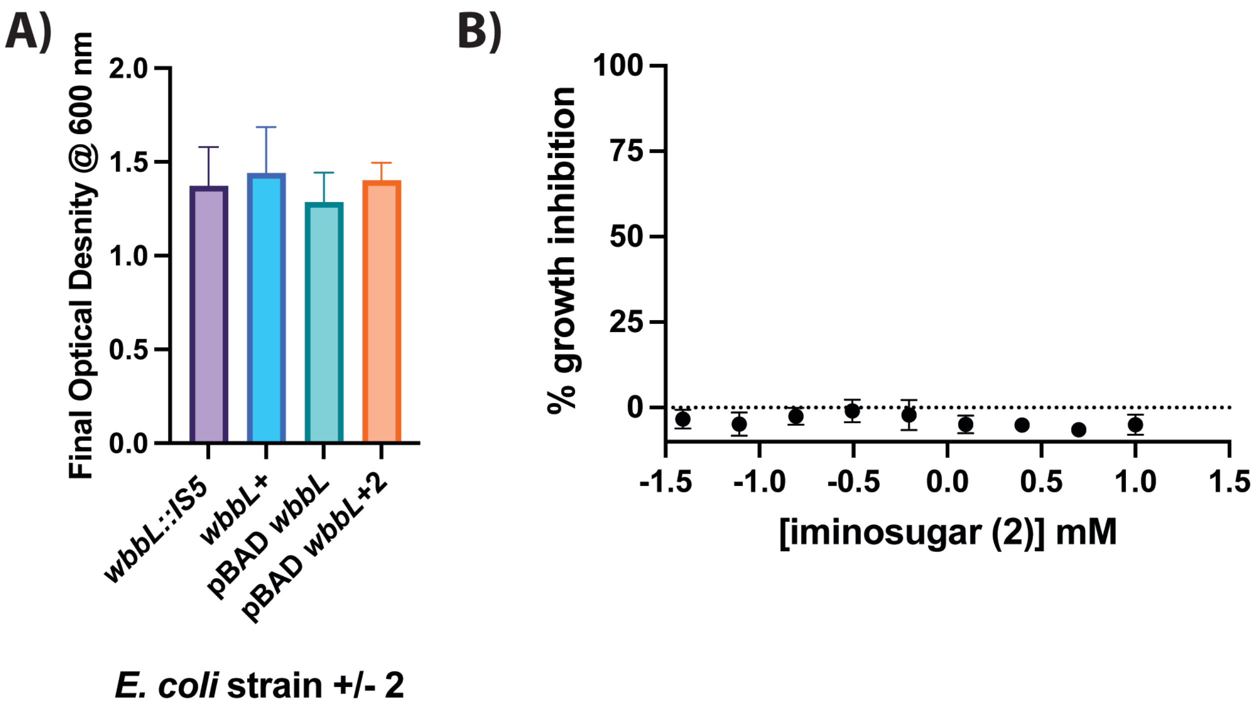


**Figure S25. Iminosugar 2 is not toxic to *E. coli* cells with or without *wbbL*.** (A) Optical density at 600 nm (OD600) measurements of *E. coli* strains shown in **Figures S23-S24** prior to normalization and LPS extraction, indicating that addition of **2** (~2 mM) to *E. coli* +/- *wbbL* did not inhibit cell growth. (B) Titration of compound **2** into *E. coli wbbL*+ indicates that the iminosugar is not toxic at the low millimolar concentrations used for cell-based experiments. Error bars represent SD (n = 3).

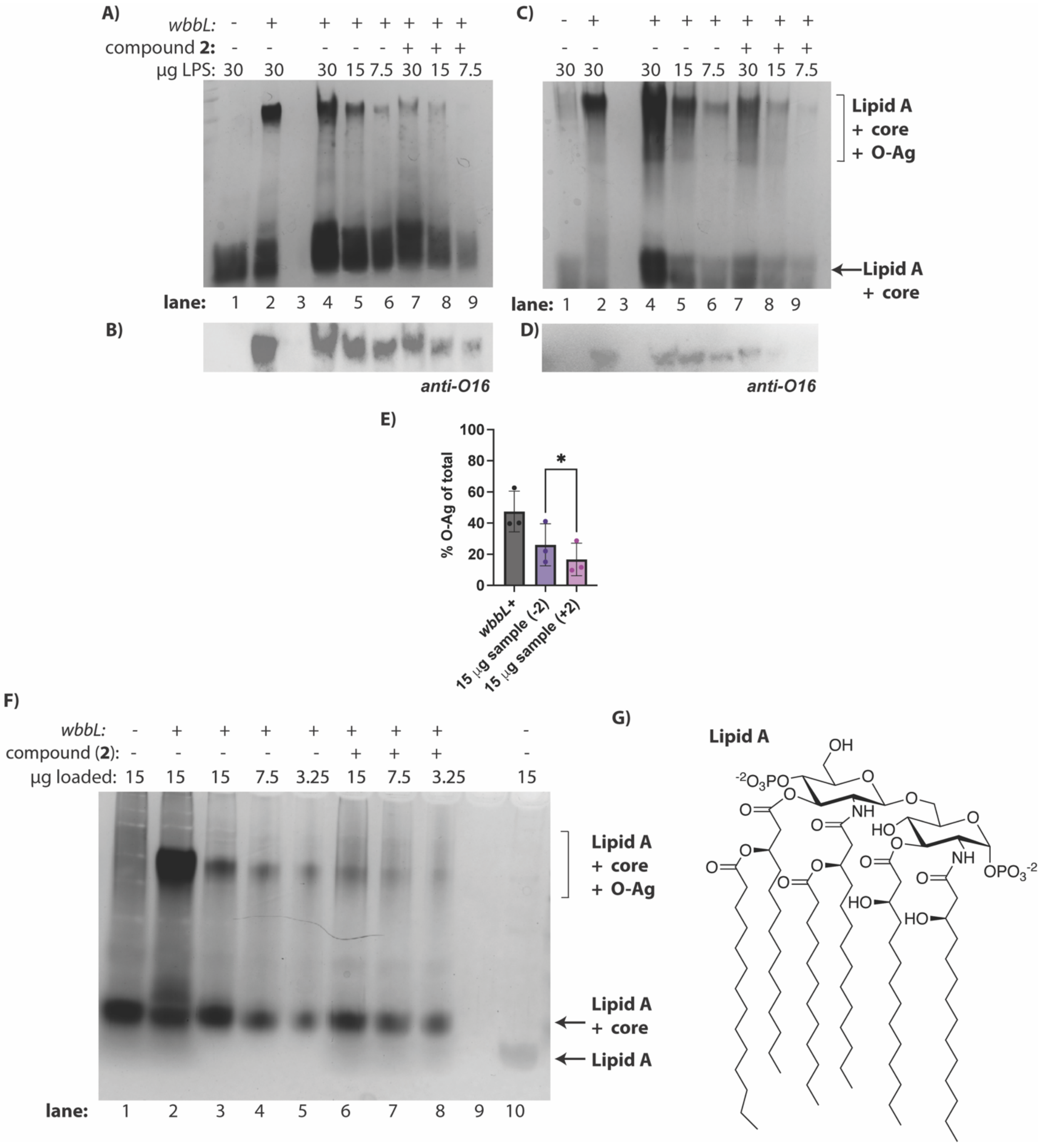


**Figure S26. Biological replicates of silver stain and O-16 blots following LPS extraction of *E. coli* cultures +/- 2 (Figure 5C).** LPS of *E. coli* +/- *wbbL* +/- **2** was extracted, separated by SDS-PAGE and visualized by silver stain (A, C) and anti-O16 immunoblotting (B, D). Replicate 2 corresponds to panels A and B, replicate 3 corresponds to panels C and D. (E) Quantification of O-Ag bands detected by silver staining of LPS from *E. coli* expressing *wbbL* +/- **2** indicates a trend of decreased production of O-Ag as a result of treatment with compound **2.** The % O-Ag of total (O-Ag = Lipid A + core + O-Ag bands) were calculated based on total signal (including Lipid A + core band) in

the same lane for lane 5 (minus **2**) and plotted versus that of lane 8 (plus **2**), which have equivalent total LPS loaded (the intermediate LPS mass loaded was chosen for this comparison). As a comparison, the % Lipid A + core + O-Ag in the control lane **2** (*wbbL*+) was also determined. Bars represent SD for n = 3 silver stained gels (shown). * $p<0.0332$. Paired t-test was used for comparison. Note that a similar method has been used for relative O-Ag quantification, and loading controls are not typically used.[8] (F) SDS-PAGE and silver stain analysis of samples from **Figure 5C** in addition to a standard, Lipid A, to aid in annotation of silver-stained gels, along with comparison to literature reports.[9] Lower concentrations of samples were loaded to minimize streaking. (G) Chemical structure of commercial Lipid A (diphosphoryl from *E. coli* F583 (Rd mutant)). Lipid A + core structure from *E. coli* K12 has been published elsewhere.[10] Strains used are described in **Figure S23**.

**Table S6. Plasmids used in this study.**

| No. | Plasmid Name | Relevant Features | Notes | References |
|---|---|---|---|---|
| 1 | pET27b | pET27b cloning vector | Novagen #69865 | N/A |
| 2 | pET-His-SUMO | pET His6 SUMO TEV LIC Cloning Vector | A gift from Scott Gradia (Addgene #29711) | N/A |
| 3 | pBAD33 | pBAD33 cloning vector | A gift from Rojas Lab (NYU) | N/A |
| 4 | pHYRS52 | His6-*S. cerevisiae* Ulp1 (res. 403-621) | Addgene #31122 | Muona et al., 2008 |
| 5 | pAPH208 | pET His6 SUMO *T. thermophilus* HB27 RfbF | Overlap extension PCR using pET-His-SUMO and primers pETHisSUMO_*T.thermophilus*_RfbF_fow and pETHisSUMO_*T.thermophilus*_RfbF_rev using *Thermus thermophilus* HB27 *rfbF* (GenBank: AAS80876.1) synthetic DNA template sequence (Twist Bioscience). | This work |
| 6 | pAPH19 | pET27b-C-term-His Mtb WbbL1 | Overlap extension PCR using pET27b and primers pET27b_Mtb_WbbL_fow and pET27b_Mtb_WbbL_rev using Mtb H37Rv genomic DNA (a gift from the Nathan Lab, Cornell Medicine) as a template (gene amplified: Rv3265c). | This work |
| 7 | pHK302 | pET27b-C-term-His *E. coli* WbbL | Overlap extension PCR using pET27b and primers pET27b_*E.coli*_WbbL_fow and pET27b_*E.coli*_WbbL_rev using genomic DNA purified from *E. coli* K-12 (Δ*rfbB51*) (strain 3) as a template. | This work |
| 8 | pAPH343 | pBAD33-C-term-FLAG *E. coli* WbbL | Overlap extension PCR using pBAD33 and primers pBAD33_*E.coli*_WbbL_fow and pBAD33_*E.coli*_WbbL_rev using genomic DNA purified from *E. coli* K-12 (Δ*rfbB51*) (strain 3) as a template. | This work |

**Table S7. Primers used in this study.**

| Oligo Name | 5' – 3' Sequence |
|---|---|
| pETHisSUMO_*T.thermophilus*_RfbF_fow | GAGGCTCACAGAGAACAGATTGGTGGGATGTCAGAAAGGGTATGTGCTGTTATAG |
| pETHisSUMO_*T.thermophilus*_RfbF_rev | CTTTCGGGCTTTGTTAGCAGCCGGATCAGTTAACACGGGCTCGTGGTCAAAC |
| pET27b_*E.coli*_WbbL_fow | AGGAAATACCTGCTGCCGACCGCTGCTGCTGGTCTGATGGTATATATAATAATCGTTTCCCACGGACATGA |
| pET27b_*E.coli*_WbbL_rev | TTAGCAGCCGGATCTCAGTGGTGGTGGTGGTGGTGCGGGTGAAAAACTGATG |
| pET27b_Mtb_WbbL_fow | AGGAAATACCTGCTGCCGACCGCTGCTGCTGGTCTGGTGGTAGCGGTGACCTACTC |
| pET27b_Mtb_WbbL_rev | TTAGCAGCCGGATCTCAGTGGTGGTGGTGGTGGTGCCGCCCTTCTACCAGCTTC |
| pBAD33_*E.coli*_WbbL_fow | TTTCTCCATACCCGTTTTTTTGGGCTAGCGAAGGAGATATATGGACTACAAAGACGATGACGACAAGGGGGGAGGGGGAGTATATATAATAATCG |
| pBAD33_*E.coli*_WbbL_rev | CTGAAAATCTTCTCTCATCCGCCAAAACAGCCTTACGGGTGAAAAACTGATGAAATTCGATCAAAGTTGC |

**Table S8. Strains used in this study.**

| No. | Strain Name | Strain Information | Source |
|---|---|---|---|
| 1 | *E. coli* MG1655 (*wbbL::*IS5) | Wildtype *E. coli* strain containing insertion in *wbbL* | Gift from Rojas lab (NYU) |
| 2 | *E. coli* MG1655 *wbbL+* | *E. coli* O-16 producing strain | Gift from Jorgenson lab (UAMS) |
| 3 | *E. coli* K-12 (Δ*rfbB51*) | Non O-Ag producing wild-type *E. coli* (contains *wbbL* but mutated in precursor biosynthesis) | *E. coli* genetic stock center (Yale) #5073 |
| 4 | BL21(DE3) | *E. coli* cells engineered for high level protein expression | Novagen |
| 5 | Rosetta2(DE3) | *E. coli* cells engineered to enhance expression of eukaryotic proteins | Novagen |
| 6 | Ec-His-SUMO-*T.thermophilus*-RfbF | Rosetta2(DE3) transformed with plasmid (5) | This work |
| 7 | Ec-pET27b-Mtb-WbbL | BL21(DE3) transformed with plasmid (6) | This work |
| 8 | Ec-pET27b-*E.coli*-WbbL | BL21(DE3) transformed with plasmid (7) | This work |
| 9 | *E. coli* MG1655 pBAD33-C-term-FLAG *E. coli* WbbL | *E. coli* MG1655 *wbbL::*IS5 transformed with plasmid (8) | This work |
| 10 | Mtb H37Rv | *Mycobacterium tuberculosis* wild-type strain | Nathan Lab (Cornell Medicine) |

**Supplemental Methods**

**General Methods.**

Primers were purchased from Invitrogen and sequencing was completed by GENEWIZ (Azenta) or EtonBio. Chemicals were purchased from Sigma Aldrich, CarboSynth, Fisher Scientific, and Cayman without further purification. $^{1}$H, $^{13}$C, and $^{31}$P NMR spectra were recorded on a Bruker Avance III 400, 500, or 600 MHz instrument. All spectra were calibrated using residual non-deuterated solvent as an internal reference and processed using MestReNova (authorized to NYU). Gel filtration purification and analyses was performing using at AKTA pure 15 L instrument (UNICORN™ software, GE Healthcare). HRMS analyses were acquired on an Agilent 6224 Accurate-Mass time-of-flight LC/MS (LC-TOF) spectrometer equipped with an electrospray ionization (ESI) source with an autosampler. GraphPad Prism software 9.0 was used for all plotting and data analysis unless otherwise noted. Statistical significance of grouped data was assessed using paired t-tests in Prism. For the IC50 calculation, data were fit in Prism using the equation log(inhibitor) versus response (three parameters): y = bottom + (top-bottom)/(1+10^(X-log IC50)) based on the assumption the Hill slope would be 1, as predicted by ITC results with n ~ 1 for donor ligands. Data was normalized to controls without enzyme and with no inhibitor added. Confidence intervals were calculated based on the standard error. 1M TEAB buffer was prepared by suspending triethylamine (1 mol, 139 mL) in water (700 mL) and passing carbon dioxide (from evaporated dry ice) through the mixture until the pH=7.5. The buffer was diluted to 1L and stored as a stock solution at 4 °C. Prior to use it was diluted to 50 mM in deionized $H_2O$ and filtered.

**Methods for protein and cellular biochemistry/bioinformatics used in this study.**

**Bioinformatic approach to identify *Thermus thermophilus* RfbF as a putative glycolipid RT.**

The Carbohydrate-Active Enzymes Database (CAZy.org) was used to identify thermophilic organisms with annotated glycosyltransferases (GTs).[11-12] Genes annotated as *wbbL* have been experimentally shown to encode dTDP-rhamnosyltransferases that transfer L-Rha onto *N*-acetyl--$\alpha$-D-glucosamine-1-diphospholipid acceptors.[13-14] In *Shigella flexneri*, the protein encoded by *rfbF* was noted to carry out the same biochemical activity as WbbL based on genetic and LPS analyses.[15-16] Both *E. coli* WbbL and *S. flexneri* RfbF belong to the GT2 family. A CAZy search for *rfbF* and *wbbL* was performed in the genomes of Gram-negative extreme thermophiles, which resulted in the identification of a GT2 in *Thermus thermophilus* (strain HB27) annotated as *rfbF* (GenBank: AAS80876.1), which had not yet been biochemically characterized. Protein ANalysis THrough Evolutionary Relationships (PANTHER) database analysis indicated that *T. thermophilus* RfbF belonged to the WbbL family.[17] Multiple sequence alignment of *E. coli* WbbL, *M. tuberculosis* WbbL, *S. flexneri* RfbF and *T. thermophilus* RfbF amino acid sequences were carried out using Clustal Omega (**Table S1**),[1] which showed that *T. thermophilus* RfbF had about ~14-28% sequence identity with each of the other glycosyltransferases. The alignment indicated conservation of a NXD/DXD motif and H residue predicted to mediate metal coordination in many GT-A fold members (**Figure S1**).[18] It has been documented that homologous GTs can show low sequence identities (such as $\leq$30 percent).[19-22] A PRotein Ortholog Search Tool (PROST) analysis of the *T. thermophilus* RfbF primary sequence was performed against sequences in the Swiss-

Prot database, as this tool was designed to detect potential homology with amino acid sequences that can have low sequence identities (<30 percent) (**Table S2**).[2] Out of the top 20 hits, 7 of the sequences are encoded by *wbbL* or *rfbF* annotated genes. Because there are no solved structures of glycosyltransferases with *N*-acetylglucosaminyl-diphospho-lipid L-rhamnosyltransferase function,[23] AlphaFold was use to model each of the GTs of interest (**Figure S2**).[3-4] Structural alignment (PyMoL, Version 2.0 Schrödinger, LLC.) of *E. coli* WbbL/Mtb WbbL, *E. coli* WbbL/*S. flexneri* RfbF and *S. flexneri* RfbF/*T. thermophilus* RfbF models demonstrated backbone root-mean-square deviation (RMSD) values of <2 Å, which is indicative of structural similarity that is typically observed for homologs.[5-6] Protein structure predictions were also compared to PHYRE2 models with PyMOL.[24-25] It should be noted that these analyses cannot confirm homology and our experimental analyses were necessary to show that RfbF and WbbL have shared biochemical function, which updates an existing functional prediction for *T. thermophilus* RfbF that suggested different substrate preferences.[26]

**Cloning of expression vectors for *E. coli* WbbL, Mtb WbbL1, and *T. thermophilus* RfbF overexpression in *E. coli.***

Overexpression plasmids (**Table S6**) were constructed by overlap extension PCR following a published protocol[27] with indicated plasmid and primer pairs (**Tables S6-S7**). *E. coli* genomic DNA was purified from the noted strain using the DNeasy Blood and Tissue Kit (Qiagen) following the manufacturer's protocol. Mtb genomic DNA was purified from the indicated strain using a published extraction technique.[28] PCR products were digested with DpnI (New England Biolabs), purified with a PCR purification kit (Qiagen), and transformed into Mach1 competent cells (Invitrogen). Upon DNA sequencing confirmation (GENEWIZ (Azenta), EtonBio) the plasmids were transformed into respective competent cells (**Table S8**) for overexpression.

**Overexpression and purification of *T. thermophilus* RfbF.**

pETHisSUMO-*T. thermophilus* RfbF was overexpressed in Rosetta2 chemically competent cells (Novagen). Overnight cultures (5 mL of LB with carbenicillin (50 µg/mL), 1 mM $MgSO_4$, and 0.2% glucose) were used to inoculate 0.5 L (1:100) of LB media supplemented with carbenicillin (50 µg/mL) and 1 mM $MgSO_4$. Cells were grown to log-phase ($OD_{600}$ = 0.6, optical density at 600 nm) with shaking (37°C, 200 rpm). Cultures were induced with 1 mM isopropyl-β-D-1-thiogalactopyranoside (IPTG) for 3 hours (25°C, 300 RPM) and harvested by centrifugation (1857 x g, 25 min, 4°C).

pETHisSUMO-*T. thermophilus* RfbF cell pellets were resuspended on ice in lysis buffer (25 mM tris(hydroxymethyl)aminomethane hydrochloride (Tris-HCl), pH 7.5, 400 mM NaCl, 10% glycerol, 1% Triton X-100 (v/v), 5 mM $MgCl_2$, 1 mM phenylmethylsulfonyl fluoride (PMSF), 5 mM tris(2-carboxyethyl)phosphine (TCEP)) and supplemented with 100 µg/mL lysozyme and 6.6 µg/mL DNase I. Cells were rocked for 30 min at 4 °C and lysed by sonication (10 min, 30 sec on,30 sec off) on ice. Cell debris was cleared by centrifugation (Beckman Coulter Allegra X-15R at 10,975 x g, 20 min at 4 °C). Supernatant was applied to 2 mL of pre-washed/equilibrated $Ni^{2+}$-NTA resin (Qiagen) and rocked for 20 min at 4 °C. The column was washed with one column volume (CV) of purification buffer (25 mM Tris-HCl, pH 7.5, 400 mM NaCl, 10% glycerol, 0.06% (w/v) n-Dodecyl-β-D-Maltoside (DDM), 5 mM $MgCl_2$, 5 mM TCEP) and subsequently washed with 2 CVs of purification buffer supplemented with 30 mM imidazole. The SUMO His-tagged protein was

eluted with 8 mL purification buffer supplemented with 200 mM imidazole and collected in 2 mL fractions. Additional SUMO His-tagged protein was eluted with 8 mL of purification buffer with 300 mM imidazole and collected in 2 mL fractions. Elution fractions containing SUMO His-tagged protein were pooled and cleaved overnight at 4°C with 300 µg of His-Ulp1[29] against 4L dialysis buffer (25 mM Tris-HCl, pH 7.5, 400 mM NaCl, 10% glycerol, 5 mM $MgCl_2$, 5 mM TCEP) in 10 kDa MWCO Slide-A-Lyzer dialysis cassettes (Thermo Scientific). The next day, the dialyzed solution was added to 2 mL of washed/equilibrated Ni-NTA agarose (Qiagen) resin and incubated at 4°C with rocking for 15 min. The flow-through and 3 x 1 mL wash fractions in purification buffer were collected. The samples containing *T. Thermophilus* RfbF were pooled and concentrated with an Amicon Ultra Centrifugal Filter Device (Millipore, 10 kDa MWCO, 1857 x g, 4 °C). Samples containing 1 mg/mL of protein were further purified by size exclusion chromatography (SEC) on a Superdex 200 Increase 20/300 GL column (GE Healthcare) using a flow rate of 0.3 mL/min with the storage buffer (25 mM Tris-HCl pH 7.5, 400 mM NaCl, 10% glycerol, 0.06% DDM, 5 mM $MgCl_2$). RfbF was injected (500 µL) and fractions containing the elution peak were concentrated as described above. The concentration of purified protein was determined by the DC protein assay (Bio-Rad) using bovine serum albumin (BSA) as a standard. Protein was aliquoted, flash frozen with $N_{2(l)}$, and stored at -80 °C. Milligram quantities of RfbF could be purified as a soluble protein in DDM-containing buffer for ITC and activity assays.

**Overexpression and membrane purification of *E. coli* WbbL and Mtb WbbL.**

pET27b-*E. coli* WbbL and pET27b-Mtb WbbL were overexpressed in BL21(DE3) chemically competent cells (Novagen). For each, overnight cultures (5 mL of LB with kanamycin (25 µg/mL), 1 mM $MgSO_4$, and 0.2% glucose) were used to inoculate 0.5 L of LB media (1:100) supplemented with kanamycin (25 µg/mL) and 1 mM $MgSO_4$. Cells were grown to log-phase ($OD_{600}$= 0.6) with shaking (37°C, 200 rpm). Cultures were induced with 1 mM IPTG for 1 hour (30°C, 300 RPM) and harvested by centrifugation (1857 x g, 25 min, 4 °C).

Cell envelope fractions were resuspended on ice in lysis buffer B (25 mM Tris-HCl, pH 7.5, 400 mM NaCl). Suspensions were sonicated for 3 minutes (1 sec on; 2 sec off). Cell debris was cleared by centrifugation (2,500 x g, 10 min). The supernatant was subjected to ultracentrifugation (Beckman Optima TLX Ultracentrifuge 120**,** 45,000 x g, 1 h, 4 °C). Pellets were extracted and resuspended in lysis buffer B. The concentration of cell envelope fractions was determined by the DC protein assay (Bio-Rad) using bovine serum albumin (BSA) as a standard. Cell envelope fractions were aliquoted, flash frozen with $N_{2(l)}$, and stored at -80 °C.

**Isothermal Titration Calorimetry (ITC) binding measurements.**

Tagless recombinant RfbF was purified as described above, and ITC was carried out at 25 °C in storage buffer using a Nano ITC low volume calorimeter (TA instruments). The following concentrations of ligand samples were used: 750 µM (dTDP-β-L-6dTal, GDP-β-L-Rha), 1 mM (dTDP-β-L-Rha, dTDP-β-L-Fuc, UDP-β-L-Rha), 1.1 mM (dTDP-β-L-Man), 1.5 mM (dTDP-α-L-Rha), 5 mM (dTDP), and 10 mM (L-Rha-1-phosphate). Ligands were titrated into 130 µL of 110 µM *T. thermophilus* RfbF. Twenty-five injections (1.78 µL/injection) were performed to achieve measurable binding events when possible. The heat of dilution was recorded by each injection of ligand into buffer under the same titration conditions. This background was subtracted from the heat of protein-ligand interactions to determine the final thermodynamic values. All

thermodynamic values were evaluated using ITCRun software (TA Instruments) and fit with a one-site binding model.

**Specific activity assays for bacterial rhamnosyltransferases from different species.**

Specific activities were determined by modification of the protocol outlined by Li et al. Specifically, 0.155 mg/mL RfbF, 0.081 mg/mL *E. coli* WbbL, or 0.111 mg/mL Mtb WbbL were used to initiate the following reaction mixtures: 25 mM Tris-HCl pH 7.5, 1 mM GlcNAc-PP-lipid, 1 mM donor-substrate, 5 mM $MgCl_2$, 1 U rSAP (1000 U/mL stock solution), 1% (v/v) Triton X-100, 5% (v/v) DMSO (for reactions containing C55, C20, C15 lipid) or MeOH (for reactions containing C35 lipid) in a total volume of 20 µL. The reaction was incubated at 37 °C (*T. thermophilus* RfbF) or 25 °C (*E. coli* WbbL and Mtb WbbL) for indicated time points (up to t = 30 min) and quenched by heating samples to 105 °C for 5 minutes at the following intervals: t = 5-, 15-, and 30-min. Controls were conducted under the same reaction conditions by omitting $MgCl_2$ and including 5 mM EDTA.

Consumption of NDP-sugar donors was monitored on reverse phase HPLC equipped with a Phenomenex 5 µm, 21.2 x 150 mm, NX-C18 Å Gemini column with solvent A (50 mM TEAB pH 8.0) and solvent B (acetonitrile) using the following linear gradient: 0% B (t = 0-5 min), 0-5% B (t = 5-25 min), 5-90% B (t = 25-35 min). Absorbance at wavelength ($\lambda$) = 254 nm was monitored. Consumption was quantified by integration of dTDP-sugar donors relative to the total amount of thymidine-containing products, which was then subtracted from the starting dTDP-sugar concentration at the t = 0 time point. Product formation was confirmed by HRMS after lyophilization (Labconco Freezone Benchtop Freeze Dryer) of reaction sample following suspension in 20 µL of 40% $NH_4HCO_3$ and 60% acetonitrile (for C15, C20, C35 lipid product) or isopropanol (for C55 lipid product) prior to transfer to a mass spectrometry V-bottom vial (Phenomenex). HRMS analyses were acquired on a LC/TOF spectrometer with an electrospray (ESI) ionization source equipped with an autosampler. Each sample (3 µL) was injected with 40% dd$H_2O$ and 60% acetonitrile as the mobile phase. Characterization of each reaction was monitored by scanning in the negative ion mode and analyzed using Agilent MassHunter software.

***In vitro* inhibition assay of *E. coli* WbbL with monosaccharides and compound 2.**

The initial linear phase of *E. coli* WbbL was determined by performing a time course with 250 µM dTDP-β-L-Rha, 1 mM GlcNAc-PP-undecaprenol and varying concentrations of WbbL. An initial rate could be determined using 0.0101 mg/mL of *E. coli* WbbL. Inhibition of *E. coli* WbbL was determined under the same conditions as the specific activity assays except 5 mM of compound **2**, L-Rha, or L-Man was incubated for 10 minutes on ice in reaction mixtures with 0.0101 mg/mL of *E. coli* WbbL and 1 mM GlcNAc-PP-undecaprenol prior to initiation of the reaction with 250 µM dTDP-β-L-Rha. Samples were quenched with heat after 8 minutes and monitored by HPLC as described above. The half maximal inhibitory concentrations ($IC_{50}$) of compound **2** were measured by incubating *E. coli* WbbL reactions with a range of concentrations of **2** from 0.3125-80 mM. Each reaction was normalized to reactions containing no enzyme (100% inhibition) or no inhibitor (0% inhibition).

**Cellular inhibition of *E. coli* WbbL with compound 2 followed by LPS extraction.**

Overnight cultures (1 mL) of *E. coli* MG1655 *wbbL*:IS5 and MG1655 *wbbL+* were used to inoculate 5 mL of LB and grown for approximately 6 hours at 37 °C. Cells were collected and frozen prior to LPS extraction as described below. C-term-FLAG *E. coli* WbbL was expressed in *E. coli* MG1655 *wbbL*:IS5 cells transformed by electroporation with pBAD33-C-term-FLAG *E. coli* WbbL. For the *wbbL* expression strains, overnight cultures (1 mL of LB with chloramphenicol (30 µg/mL) and 1 mM $MgSO_4$) with or without 2.5 mM compound **2** were used to inoculate 5 mL LB supplemented with chloramphenicol (30 µg/mL) and 1 mM $MgSO_4$ with or without 1.75 mM compound **2**. Cells were grown to log-phase ($OD_{600}$= 0.6) with shaking (37 °C, 200 rpm). Cultures were induced with 1.33 mM arabinose for 3 hour (25 °C, 200 RPM). 4.5 mL of cultures were normalized to an equivalent number of cells and harvested by centrifugation (1857 x g, 25 min, 4 °C). Cell pellets were stored at -80 °C prior to cell lysis and LPS extraction.

LPS extraction and silver staining were achieved by adapted published protocols.[30-31] Briefly, cells were washed with 2 x 2 mL PBS supplemented with 0.15 mM $CaCl_2$ and 0.5 mM $MgCl_2$. Cells were sonicated in 2 mL PBS for 5 min (30 sec on; 30 sec off). Lysed cells were incubated at 65 °C for one hour with Proteinase K (100 µg/mL, New England Biolabs). Cells were supplemented with RNase A (40 µg/mL), DNase I (20 µg/mL), $MgSO_4$ (1 µL/mL), and chloroform (4 µL/mL) and incubated at 37 °C overnight. The next day, cells were treated with phenol (2 g) pre-heated at 65 °C and mixed vigorously for 15 min. The mixture was cooled to 0 °C for 15 min and subjected to centrifugation (1857 x g, 10 min, 4 C). The aqueous phase was removed, and the organic phase was extracted with dd$H_2O$ (2 mL) preheated to 65 °C. Cells were cooled and centrifuged as described above. The combined aqueous phases (~4 mL) were diluted with ethanol (10 mL) and treated with sodium acetate (final concentration 0.5 M) and cooled overnight (-20 °C). The sample was centrifuged (1857 x g, 10 min, 4 °C), the pellet was resuspended in dd$H_2O$ (1 mL) and dialyzed against dd$H_2O$ (4 L) for 4 days. The resulting product was lyophilized to yield a white powder and stored at 4 °C indefinitely.

**Analysis of potential minimum inhibitory concentration of 2 with *E. coli*.**

*E. coli wbbL+* was grown in 5 mL of LB overnight to late log phase and the $OD_{600}$ was recorded prior to washing. Cells were pelleted by centrifugation (1857 x g, 5 min, 4 °C) prior to washing with 10 mL 1x phosphate buffer saline (PBS). Following washing and centrifugation, cells were diluted to a $OD_{600}$ of 0.01. Iminosugar (**2**) (100x stock) was added to cultures (200 µL cells per well) in a 96-clear bottom plate (Corning) with control wells containing water as the vehicle or LB only. Cells were grown at 37 °C with shaking and $OD_{600}$ was measured using a plate reader (SpectraMax iD5 Multi-Mode Microplate Reader, Molecular Devices) over t = 6 hr. Percent growth inhibition shown was calculated at late log phase ($OD_{600}$ ~ 0.8). Percent growth inhibition for each well was calculated using the following equation: $((OD_{600,\ vehicle} - OD_{600,\ well})/(OD_{600,\ vehicle}))$ X 100, where the $OD_{600,\ vehicle}$ is the well that contained only vehicle for the tested condition. Resulting data was plotted in Prism.

**Silver staining and western blot visualization of LPS extractions.**

30, 15, or 7.5 µg of purified LPS that had been treated with or without compound **2** was loaded onto 4-12% gradient gels and analyzed by SDS-PAGE by running gel at 200 V for 40 min in 1x SDS-PAGE running buffer (25 mM Tris, 191 mM glycine, 0.1% (w/v) SDS). The gel was rocked

overnight in fixing solution (55% dd $H_2O$, 40% EtOH, 5% acetic acid, 200 mL), which was replaced the following day with fixing solution supplemented with 0.7% periodic acid (5 min). The gel was washed 3x in dd$H_2O$ (200 mL, 15 min) and treated with freshly prepared staining solution (0.019 M NaOH, concentrated 0.193 M $NH_4OH$, 0.67% $AgNO_3$, 150 mL). The gel was washed 3x in dd$H_2O$ (200 mL, 15 min) and developed with freshly prepared developing solution (0.26 mmol citric acid, 0.0185% (v/v) formaldehyde, 500 mL), and was allowed to stain until the desired intensity had been achieved.

For western blot analysis, samples were analyzed by SDS-PAGE as described above for silver staining. Immunoblotting was performed by transfer onto polyvinylidene difluoride membrane (PVDF) at 25V, 10A, for 30 min and incubated with blocking buffer (2.5% milk fat in 1x TBS buffer supplemented with 0.1% Tween) for 1 hr at 25 °C. Primary antibody was incubated overnight at 4 °C blocking buffer with the following dilution, (anti-O16, 1:2k, SSI Dianostica). Secondary antibody (anti-Rabbit HRP, 1:10k, BioLegend) in blocking buffer was also incubated overnight at 4 °C prior to imaging with Clarity Max™ Western ECL Substrate (Bio-Rad).

**Determination of colony forming unit/mL (CFU/mL) for *E. coli* +/- *wbbL* grown +/- compound 2.**

Cells (100 µL) from each strain grown +/- **2** as described above were normalized according to $OD_{600}$ values and ten-fold serially diluted (10-fold) in PBS, 2 µL of each dilution ($10^0$-$10^{-8}$) was plated on LB and incubated overnight at 37 °C. Normalized cells were used for LPS extractions described above. Cells were counted to determine the CFU for each strain based on known dilution factors.

**Docking of ligands to *E. coli* WbbL model.**

Molecular docking was performed using Glide (Schrödinger Release 2021–2) loaded with the AlphaFold predicted structure of *E. coli* WbbL.[3-4] $Mg^{2+}$ ion was introduced after alignment with Mtb Gal*f*T2 (PDB ID: 4fiy). Gal*f*T2 was chosen because it has similar biochemical function to the top hit from the PROST search (**Table S2**) and there are solved crystal structures available. Default parameters were used for optimization, and an OPLS3e force field was employed. The Glide docking grid of the receptor was generated to include the $Mg^{2+}$ and predicted substrate binding pockets based on conserved residues found by multiple sequence alignments (**Figure S2**). Default van der Waals radii parameters were used. 3D structure of dTDP-β-L-Rha, α/β-L-Man and iminosugar (**2**) were generated following LigandPrep Wizard using the structure of rhamnosyltransferase-bound dTDP-β-L-Rha from a published crystal structure (PDB ID: 6J7K). 10 poses per ligand were generated. The indicated scores are GlideScores, which approximate the free binding energies of each ligand.

**Methods for syntheses of compounds used in this study.**

**Nucleotide sugars.** Unless otherwise noted, nucleotide sugars were purchased (Carbosynth, Sigma). dTDP-β-L-Fuc, dTDP-α-Glc, and dTDP-β-L-Man were obtained chemoenzymatically and purified following a published protocol; dTDP-β-L-Rha, dTDP-β-L-6dTal, dTDP-α-L-Rha, GDP-β-L-Rha, and UDP-β-L-Rha were accessed through synthetic routes and purified as recently reported.[32-33]

**2-Acetamido-3, 4, 6-tri-*O*-acetyl-2-deoxy-D-glucopyranose (S1)**

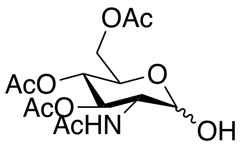


Compound **S1** was prepared according to an adapted published protocol.[34] Briefly, to a flask containing 2-Acetamido-1, 3, 4, 6-tetra-*O*-acetyl-2-deoxy-β-D-glucopyranose (1 g, 3 mmol), hydrazine acetate (300 mg, 3 mmol) in DMF (15 mL) was added and stirred at 25 °C for 2 hr. The reaction was diluted with water, extracted with ethyl acetate (3X) and washed with 1 M HCl. The resulting organic fractions were dried over $Na_2SO_4$, filtered, and concentrated under pressure. The remaining dimethylformamide (DMF) was evaporated using toluene to afford a 9:1 α/β mixture of the desired compound as a white foam (742 mg, 80%). Spectral values were observed in accordance with the literature. $^1$H NMR (400 MHz, $CDCl_3$) δ 6.18 (d, *J* = 9.3 Hz, 1H), 5.27 (t, *J* = 9.3 Hz, 1H), 5.21 (d, *J* = 3.5 Hz, 1H), 5.09 (t, *J* = 9.4 Hz, 1H), 4.28 – 4.22 (m, 1H), 4.22 – 4.17 (m, 2H), 4.10 – 4.04 (m, 1H), 2.06 (s, 3H), 2.01 (d, *J* = 2.3 Hz, 3H), 2.00 (s, 3H), 1.94 (s, 3H). $^{13}$C NMR (126 MHz, $CDCl_3$) δ 171.55, 171.20, 171.02, 169.65, 91.77, 71.09, 68.42, 67.41, 62.24, 52.43, 23.12, 21.14, 20.82, 20.49. HRMS (*m/z*): $[M+H]^+$ calcd. for $C_{14}H_{22}NO_9$ 348.1288; found 348.1289; deviation (ppm): -0.17

**2-Acetamido-3,4,6-tri-*O*-acetyl-2-deoxy-α-D-glucopyranose 1-dibenzylphosphate (S2)**

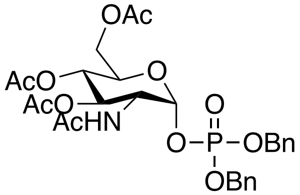


Compound **S2** was prepared according to an adapted published protocol.[35] Briefly, to a solution of compound **S1** (550 mg, 1.58 mmol) in anhydrous tetrahydrofuran (THF) (10 mL) was added a solution of 1*H*-tetrazole in acetonitrile (10 mL, 0.45 M 4.5 mmol) followed by dibenzyl *N,N*-diisoproylphosphoramidite (1.05 mL, 3.17 mmol) at 25 °C for 14 h. The solution was cooled to -78 °C and (30% v/v) $H_2O_2$ (297 µL) was added dropwise. The reaction mixture was stirred at -78 °C for one hour and allowed to warm to room temperature prior to quenching with saturated aqueous $Na_2SO_3$. The reaction mixture was diluted with diethyl ether and washed with brine. The resulting organic layer was dried over $Na_2SO_4$, filtered, and concentrated under reduced pressure. The product was purified by silica column chromatography (2:3, 1:3, 0:1 hexanes/EtOAc with 1% $Et_3N$) that afforded compound **S2** (600 mg, 62%) as a white solid. $^1$H NMR (400 MHz,

$CDCl_3$) δ 7.35 (dq, *J* = 7.7, 4.8, 4.2 Hz, 10H), 5.84 (d, *J* = 9.2 Hz, 1H), 5.66 (dd, *J* = 6.0, 3.3 Hz, 1H), 5.16 – 5.11 (m, 2H), 5.11 – 5.00 (m, 4H), 4.42 – 4.32 (m, 1H), 4.12 (dd, *J* = 12.5, 3.9 Hz, 1H), 3.99 (dt, *J* = 5.8, 3.6 Hz, 1H), 3.91 (dd, *J* = 12.5, 2.0 Hz, 1H), 2.01 (s, 3H), 2.00 – 1.98 (m, 6H), 1.70 (s, 3H). $^{13}$C NMR (126 MHz, $CDCl_3$) δ 171.24, 170.67, 170.39, 169.24, 135.40 (d, *J* = 6.4 Hz), 135.25 (d, *J* = 6.5 Hz), 129.06, 128.93, 128.89, 128.21, 128.17, 96.33 (d, *J* = 6.5 Hz), 70.14, 70.09, 70.04, 69.70, 67.40, 61.34, 51.81 (d, *J* = 7.8 Hz), 22.81, 20.75, 20.71, 20.66. $^{31}$P NMR (162 MHz, $CDCl_3$) δ -2.52. HRMS (*m/z*): $[M+Na]^+$ calcd. for $C_{28}H_{34}NO_{12}PNa$ 630.1711; found 630.1735; deviation (ppm): 3.84

**2-Acetamido-3,4,6-tri-*O*-acetyl-2-deoxy-α-D-glucopyranose 1-phosphate (S3)**

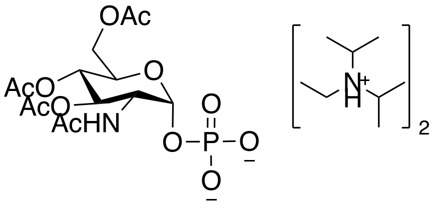

Compound **S3** was prepared according to an adapted published protocol.[34-35] Briefly, compound **S2** (430 mg, 0.708 mmol) was dissolved in anhydrous methanol (3 mL) and added to a flask containing $Pd(OH)_2$ (72 mg, 0.510 mmol) on carbon (20 %, wt/vol) moistened with ethyl acetate and diluted with anhydrous methanol (2 mL). The mixture was stirred at 25 °C for 1 hr under $H_2$. The flask was evacuated with $N_2$ followed by the addition of *N,N*-diispropylethylamine (0.185 mL, 1.06 mmol). The reaction mixture was diluted with methanol (3 mL) and allowed to stir for 1 hr. The solution was filtered through a syringe containing a Celite pad and concentrated under pressure to afford compound **S3** (300 mg, 99%) as a colorless oil. $^1$H NMR (400 MHz, MeOD) δ 5.47 (dd, *J* = 7.0, 3.4 Hz, 1H), 5.32 – 5.23 (m, 1H), 5.06 (t, *J* = 9.8 Hz, 1H), 4.30 – 4.25 (m, 2H), 4.24 – 4.20 (m, 1H), 4.12 – 4.06 (m, 1H), 3.73 – 3.66 (m, 2H, DIPEA CH), 3.19 (t, *J* = 7.4 Hz, 4H, DIPEA $CH_2$), 2.02 (s, 3H), 1.98 (s, 3H), 1.94 (s, 3H), 1.92 (s, 3H), 1.37 – 1.34 (m, 30H, DIPEA $CH_3$). $^{13}$C NMR (101 MHz, MeOD) δ 173.25, 172.13, 171.67, 171.04, 94.78 (d, *J* = 5.9 Hz), 72.57, 69.64, 69.27, 62.76, 55.32 (DIPEA CH), 53.23 (d, *J* = 7.8 Hz), 43.33 (DIPEA $CH_2$), 22.39, 20.48, 20.43, 17.90 (DIPEA $CH_3$), 12.91 (DIPEA $CH_3$). $^{31}$P NMR (162 MHz, MeOD) δ -1.34 ppm. HRMS (*m/z*): $[M-H]^-$ calcd. for $C_{14}H_{21}NO_{12}P$ 426.0807; found 426.0815; deviation (ppm): 1.89

**2-Acetamido-3,4,6-tri-*O*-acetyl-2-deoxy-α-D-glucopyranose 1-phosphoimidazolide (S4)**

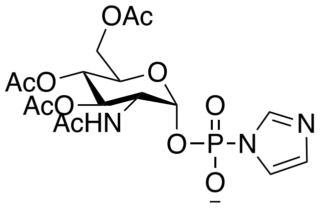

Compound **S4** was prepared according to a published protocol as briefly described.[34] A flask containing a solution of compound **S3** (80 mg, 0.19 mmol) and *N*, *N'*-Carbonyldiimidazole (CDI) (80 mg, 0.5 mmol) in THF (3 mL), under argon, was mixed at 25 °C for 2 hr. The formation of CDI-activated peracetylated GlcNAc-1-phosphate was initially monitored using LRMS under negative mode. The reaction was also monitored by $^{31}$P NMR. The reaction was quenched with methanol

(50 μL) for 1 hr at 25 °C to remove excess CDI, and then concentrated to afford **S4** crude product that was used immediately in the diphosphate coupling steps. $^{31}$P NMR (202 MHz, MeOD) δ -11.85. LRMS (*m/z*): [M-H]$^-$ calcd. For $C_{17}H_{22}N_2O_{12}P$ 476.1; found 475.9

**Farnesyl-, geranylgeranyl-, heptaprenyl- and undecaprenyl monophosphates**
Farnesol and geranylgeraniol were from Sigma-Aldrich-Fluka, Poznan; while, heptaprenol and undecaprenol were from the Collection of Polyprenols, IBB PAS, Warsaw. Farnesyl-, geranylgeranyl-, heptaprenyl- and undecaprenyl monophosphates were synthesized as described previously.[36]

**P$^1$-2-Acetamido-3,4,6-tri-*O*-acetyl-2-deoxy-α-D-glucopyranosyl P$^2$-(2Z,6Z)-3,7,11-trimethyldodeca-2,6,10-trien-1-yl diphosphate (S5)**

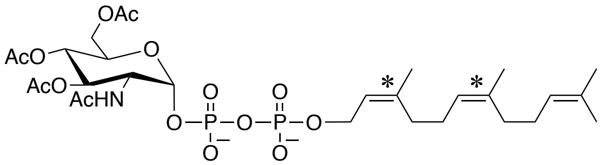


Glycolipids **S5-S8** and **1a-d** were prepared according to adapted published protocols[34, 37] as described in the sections below. A solution of **S4** (39 mg, 0.083 mmol) was added to a flask containing monophosphate-farnesyl (*trans:cis* 40:60) (10 mg, 0.033 mmol) in 2 mL of anhydrous THF and the reaction was stirred for 2 days at 25 °C, under inert atmosphere. The reaction was monitored with $^{31}$P NMR. The resulting solution was concentrated under pressure and resuspended in 0.5 mL of water. The product was purified using a 4 mL C-18 reverse phase column and eluted with 1.7% $NH_4HCO_3$(aq):acetonitrile (100:0 to 65:35, stepwise). Fractions containing **S5** were lyophilized and resulted in 7.5 mg of **S5** as a white powder (32% yield). $^1$H NMR (500 MHz, MeOD) δ 5.58 (dd, *J* = 4.3, 2.1 Hz, 1H), 5.46 (t, *J* = 6.9 Hz, 1H), 5.33 (ddd, *J* = 10.6, 9.3, 1.1 Hz, 1H), 5.18 – 5.04 (m, 3H), 4.54 (q, *J* = 6.8 Hz, 2H), 4.39 (dt, *J* = 10.2, 2.7 Hz, 1H), 4.32 (ddd, *J* = 15.5, 12.9, 3.1 Hz, 2H), 4.18 (dt, *J* = 12.4, 1.9 Hz, 1H), 2.28 – 2.02 (m, 8H), 2.03 (s, 3H), 2.00 (s, 3H), 1.98 (dd, *J* = 5.0, 1.5 Hz, 3H), 1.95 (s, 3H), 1.73 (dd, *J* = 19.4, 1.3 Hz, 3H), 1.67 (d, *J* = 1.5 Hz, 3H), 1.66 – 1.50 (m, 6H). $^{13}$C NMR (151 MHz, MeOD) δ 174.39, 172.84, 172.17, 171.65, 141.13, 136.74, 136.48, 132.39, 125.74, 125.53, 125.45*, 123.62 (d, *J* = 8.5 Hz), 122.74, 96.04, 73.30, 70.17, 69.99, 64.25*, 63.97, 63.14, 53.51 (d, *J* = 8.2 Hz), 41.17, 40.95, 33.39, 31.06*, 28.10, 28.08, 27.79, 26.17, 24.03, 23.05, 20.97, 20.93, 20.92, 18.05, 18.03, 16.90, 16.40, 16.38. $^{31}$P NMR (202 MHz, MeOD) δ -10.33 (d, *J* = 21.6 Hz), -13.43 (d, *J* = 21.3 Hz). HRMS (*m/z*): [M-H]$^-$ calcd. for $C_{29}H_{46}NO_{15}P_2$ 710.2348; found 710.2340; deviation (ppm): -4.58
*indicates mixture of cis:trans (60:40) isomer form, as reported for farnesyl-monophosphate starting material

0.1% Sodium methoxide in methanol (2.5 mL) was added to **S5** (4 mg, 0.0056 mmol) and mixed for 1 hr at 25 °C. The solvent was removed in vacuo and the product was lyophilized to produce a **1a** as a white powder (3 mg, 91%). HRMS (*m/z*): [M-H]$^-$ calcd. for $C_{23}H_{40}NO_{12}P_2$ 584.2031; found 584.2015; deviation (ppm): -2.86

P[1]-2-Acetamido-2-deoxy-α-D-glucopyranosyl P[2]-(2Z,6Z)-3,7,11-trimethyldodeca-2,6,10-trien-1-yl diphosphate **(1a)**

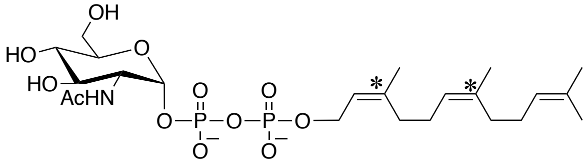

**P[1]-2-Acetamido-3,4,6-tri-*O*-acetyl-2-deoxy-α-D-glucopyranosyl P[2]-(2E,6E,10E)-3,7,11,15-tetramethyl hexadeca-2,6,10,14-tetraen-1-yl diphosphate (S6)**

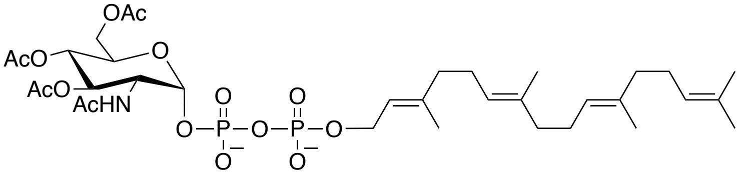

A solution of **S4** (40 mg, 0.084 mmol) was added to a flask containing monophosphate-geranylgeranyl (10 mg, 0.0271 mmol) and the reaction was stirred for 2.5 days at 25 °C in DMF (1.5 mL), under inert atmosphere ($N_2$). The resulting solution was concentrated under pressure and resuspended in 0.8 mL water. The product was purified using a C-18 reverse phase column and eluted with 1.7% $NH_4HCO_3$(aq):acetonitrile (100:0 to 70:30). Fractions containing **S6** were lyophilized and resulted as a white powder (21 mg, 66 %). $^1$H NMR (500 MHz, MeOD) δ 5.55 (dd, *J* = 7.3, 3.4 Hz, 1H), 5.42 (t, *J* = 6.9 Hz, 1H), 5.30 (t, *J* = 10.0 Hz, 1H), 5.12 – 5.03 (m, 4H), 4.51 (t, *J* = 6.5 Hz, 2H), 4.35 (dd, *J* = 10.1, 2.8 Hz, 1H), 4.34 – 4.21 (m, 2H), 4.16 (dd, *J* = 12.6, 2.4 Hz, 1H), 2.13 – 2.01 (m, 12H), 1.98 (s, 3H), 1.96 (s, 3H), 1.94 (s, 3H), 1.68 (s, 3H), 1.64 (s, 3H), 1.58 (s, 3H), 1.57 (s, 6H). $^{13}$C NMR (151 MHz, MeOD) δ 174.41, 172.96, 172.35, 171.76, 141.32, 136.47, 136.15, 132.36, 125.68, 125.51, 122.47 (d, *J* = 9.2 Hz), 95.98 (d, *J* = 6.3 Hz), 73.19, 70.14, 69.92, 64.23 (d, *J* = 5.6 Hz), 63.10, 53.46 (d, *J* = 8.5 Hz), 41.08, 41.05, 40.95, 28.03, 27.87, 27.80, 26.19, 23.06, 21.00, 20.96, 18.06, 16.93, 16.40. $^{31}$P NMR (202 MHz, MeOD) δ -10.37 (d, *J* = 21.3 Hz), -13.34 (d, *J* = 21.3 Hz). HRMS (*m/z*): $[M-H]^-$ calcd. for $C_{34}H_{54}NO_{15}P_2$ 778.2974; found 778.2974; deviation (ppm): -1.73

0.1% Sodium methoxide (1 mL) in methanol was added to **S6** (1 mg, 0.00319 mmol) and mixed for 1 hr at 25 °C. Product was lyophilized to produce a white powder (0.8 mg, 64%) and stored at -20 °C until further use. HRMS (*m/z*): $[M-H]^-$ calcd. for $C_{28}H_{49}NO_{12}P_2$ 653.2730; found 653.2751; deviation (ppm): 3.18

P[1]-2-Acetamido-2-deoxy-α-D-glucopyranosyl P[2]-(2E,6E,10E)-3,7,11,15-tetramethyl hexadeca-2,6,10,14-tetraen-1-yl diphosphate **(1b)**

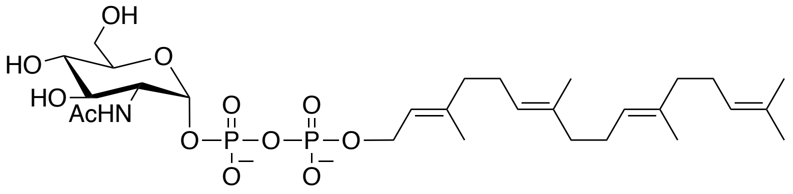


**P[1]-2-Acetamido-3,4,6-tri-*O*-acetyl-2-deoxy-α-D-glucopyranosyl P[2] -(2Z,6Z,10Z,14Z,18E,22E)-3,7,11,15,19,23,27-heptamethyloctacosa-2,6,10,14,18,22,26-heptaen-1-yl diphosphate (S7)**

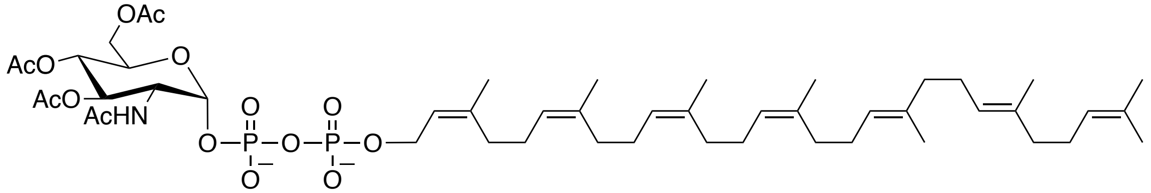


A solution of **S4** (44 mg, 0.0910 mmol) was added to a flask containing monophosphate-heptaprenyl (15 mg, 0.0260 mmol) and the reaction was stirred for 1 day at 25 °C in THF (2 mL), under inert atmosphere. The resulting solution was concentrated under pressure and resuspended in 0.8 mL water. The product was purified using a C-18 reverse phase column and eluted with 1.7% $NH_4HCO_3$(aq):acetonitrile (100:0 to 30:70). Fractions containing **S7** were lyophilized and resulted in a white powder (5.5 mg, 22%). $^1$H NMR (500 MHz, MeOD) δ 5.58 (dd, *J* = 7.3, 3.4 Hz, 1H), 5.46 (td, *J* = 6.9, 1.6 Hz, 1H), 5.33 (dd, *J* = 10.6, 9.4 Hz, 2H), 5.16 – 5.08 (m, 6H), 4.53 (t, *J* = 6.7 Hz, 2H), 4.39 (dt, *J* = 10.2, 2.8 Hz, 1H), 4.32 (ddd, *J* = 10.6, 8.6, 2.9 Hz, 2H), 4.20 (dd, *J* = 12.5, 2.3 Hz, 1H), 2.15 – 2.06 (m, 24H), 2.06 (s, 3H), 2.00 (s, 3H), 1.99 (s, 3H), 1.96 (s, 3H), 1.75 (s, 3H), 1.68 (s, 3H), 1.68 (s, 9H), 1.62 (s, 3H), 1.60 (s, 6H). $^{13}$C NMR (151 MHz, MeOD) δ 172.64, 171.13, 170.48, 169.90, 139.24, 138.48, 135.02, 134.91, 134.83, 134.65, 134.48, 130.64, 124.79, 124.75, 124.54, 124.09, 124.05, 122.03 (d, *J* = 8.4 Hz), 94.36 (d, *J* = 6.0 Hz), 71.54, 68.53, 68.28, 62.30 (d, *J* = 5.5 Hz), 61.43, 51.83 (d, *J* = 8.3 Hz), 39.48, 39.44, 31.92, 31.87, 31.51, 26.45, 26.27, 26.25, 26.21, 26.15, 24.56, 22.45, 22.40, 22.36, 22.35, 21.38, 19.31, 19.27, 19.25, 16.43, 14.79. $^{31}$P NMR (202 MHz, MeOD) δ 1.37, -10.05 (d, *J* = 20.7 Hz), -13.00 (d, *J* = 20.9 Hz). HRMS (*m/z*): [M-H]$^-$ calcd. for $C_{49}H_{78}NO_{15}P_2$ 982.4852; found 982.4860; deviation (ppm): 0.83

0.1% Sodium methoxide (1 mL) in methanol was added to **S7** (2.5 mg, 0.00254 mmol) and was mixed for 90 min at 0 °C. Product was lyophilized to produce a white powder (0.8 mg, 95% yield) and stored at -20 °C until further use. HRMS (*m/z*): [M-H]$^-$ calcd. for $C_{43}H_{72}NO_{12}P_2$ 854.4635; found 856.4630; deviation (ppm): -0.584

P[1]-2-Acetamido-2-deoxy-α-D-glucopyranosyl P[2]-(2Z,6Z,10Z,14Z,18E,22E)-3,7,11,15,19,23,27-heptamethyloctacosa-2,6,10,14,18,22,26-heptaen-1-yl diphosphate **(1c)**

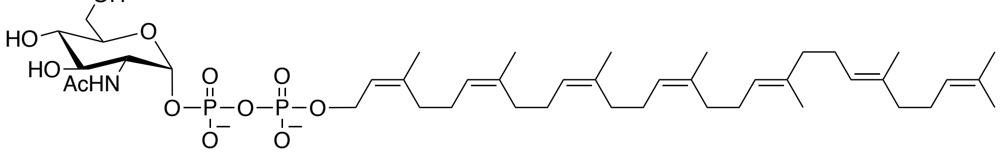

**$P^1$-2-Acetamido-3,4,6-tri-*O*-acetyl-2-deoxy-α-D-glucopyranosyl $P^2$ -(2Z,6Z,10Z,14Z,18Z,22Z,26Z,30E,34E,38E)-3,7, 11,15,19,23,27,31,35,39,43-undecamethyltetratetraconta-2,6,10,14,18,22,26,30,34,38,42-undecaen-1-yl diphosphate (S8)**

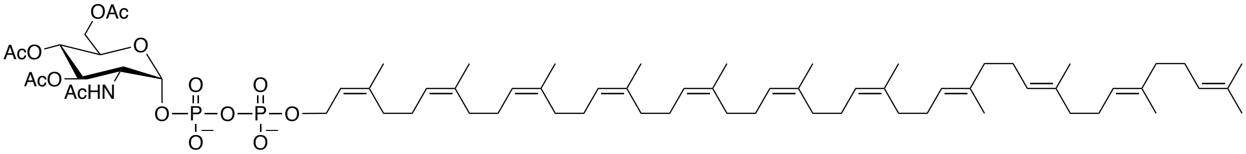

A solution of **S4** (35 mg 0.062mmol) was added to a flask containing monophosphate-undecaprenyl (15.0 mg, 0.018 mmol) and stirred in THF (3 mL) for 2.5 days at 25 °C, under argon. The resulting solution was concentrated under pressure and resuspended in 0.5 mL water. The product was purified using C-18 reverse phase column and eluted with 1.7% $NH_4HCO_3$ (aq):isopropanol (100:0 to 20:80). Fractions containing **S8** were lyophilized and resulted in a white powder (14 mg, 64% yield). $^1$H NMR (600 MHz $CD_3OD$) δ 5.55 (d, *J* = 7.6 Hz, 1H,), 5.44 (t, *J* = 6.9 Hz, 1H), 5.31 (t, *J* = 10.0 Hz, 1H), 5.13 – 5.07 (m, 10H), 4.51 (t, *J* = 6.9 Hz, 2H**),** 4.37 (d, *J* = 10.4 Hz, 1H), 4.33 – 4.23 (m, 3H), 4.15 (d, *J* = 12.6 Hz, 1H), 2.14 – 1.98 (m, 40H), 1.96 (t, *J* = 6.9 Hz, 9H), 1.92 (s, 3H), 1.72 (s, 3H), 1.65 (d, *J* = 7.6 Hz, 21H), 1.59 (s, 3H), 1.57 (s, 9H). $^{13}$C NMR (151 MHz, DMSO) δ 173.82, 172.22, 171.53, 171.03, 140.22, 136.07, 135.99, 135.98, 135.94, 135.93, 135.89, 135.73, 135.52, 135.51, 131.74, 125.92, 125.69, 125.23, 125.21, 125.17, 123.22 (d, *J* = 9.4 Hz), 95.51, 72.71, 69.60, 69.37, 63.40 (d, *J* = 5.6 Hz), 62.53, 52.94 (d, *J* = 8.4 Hz), 40.62, 40.60, 40.55, 33.06, 33.01, 32.99, 32.97, 32.64, 30.51, 30.21, 27.57, 27.46, 27.41, 27.38, 27.36, 27.33, 27.26, 25.70, 23.64, 23.62, 23.55, 23.50, 23.48, 22.49, 20.42, 20.39, 20.37, 17.57, 15.94, 15.92, 14.19. $^{31}$P NMR (162 MHz, $CDCl_3$) δ -8.85 (d, *J* = 21.2 Hz), -10.52 (d, *J* = 20.5 Hz). HRMS (*m/z*): $[M-H]^-$ calcd. for $C_{69}H_{110}NO_{15}P_2$ 1254.7356; found 1254.7410; deviation (ppm): 4.32

0.1% Sodium methoxide (1 mL) in methanol was added to **S8** (4 mg, 0.003 mmol) and was mixed for 1 hr at 25 °C. Product was lyophilized to produce a white powder (3.4 mg, 90% yield) stored at -20 °C until further use. HRMS (*m/z*): $[M-H]^-$ calcd. for $C_{63}H_{104}NO_{12}P_2$ 1128.7039; found 1128.6997; deviation (ppm): -3.72

$P^1$-2-Acetamido-2-deoxy-α-D-glucopyranosyl $P^2$-(2Z,6Z,10Z,14Z,18Z,22Z,26Z,30E,34E,38E)-3,7, 11,15,19,23,27,31,35,39,43-undecamethyltetratetraconta-2,6,10,14,18,22,26,30,34,38,42-undecaen-1-yl diphosphate **(1d)**

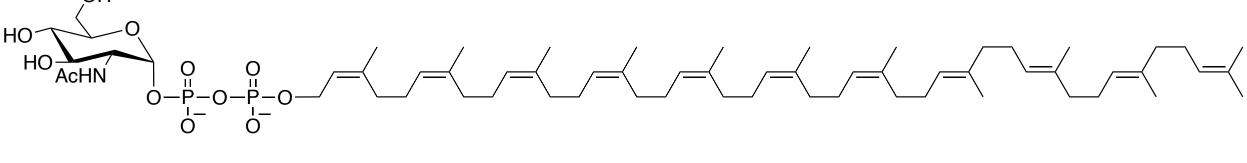

**1,5-dideoxy-1,5-imino-L-rhamnitol (2)**

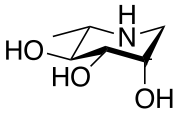


Compound **2** was synthesized as reported by Crich *et. al.*[38] and the NMR spectra were consistent with previous reports. $^{1}$H NMR (400 MHz, MeOD) δ 4.10 (td, *J* = 3.1, 1.5 Hz, 1H), 3.61 (t, *J* = 9.6 Hz, 1H), 3.50 (dd, *J* = 9.2, 2.9 Hz, 1H), 3.27 (dd, *J* = 13.2, 3.1 Hz, 1H), 3.17 (dd, *J* = 13.2, 1.6 Hz, 1H), 2.99 (dq, *J* = 10.0, 6.5 Hz, 1H), 1.42 (d, *J* = 6.5 Hz, 3H).$^{13}$C NMR (101 MHz, MeOD) δ 74.41, 71.96, 67.70, 57.11, 49.13, 15.49. HRMS (*m/z*): $[M+H]^+$ calcd. for $C_6H_{14}NO_3$ 148.0968 ; found 148.0958.

**$^{1}$H NMR 2-Acetamido-3, 4, 6-tri-*O*-acetyl-2-deoxy-D-glucopyranose (S1)**

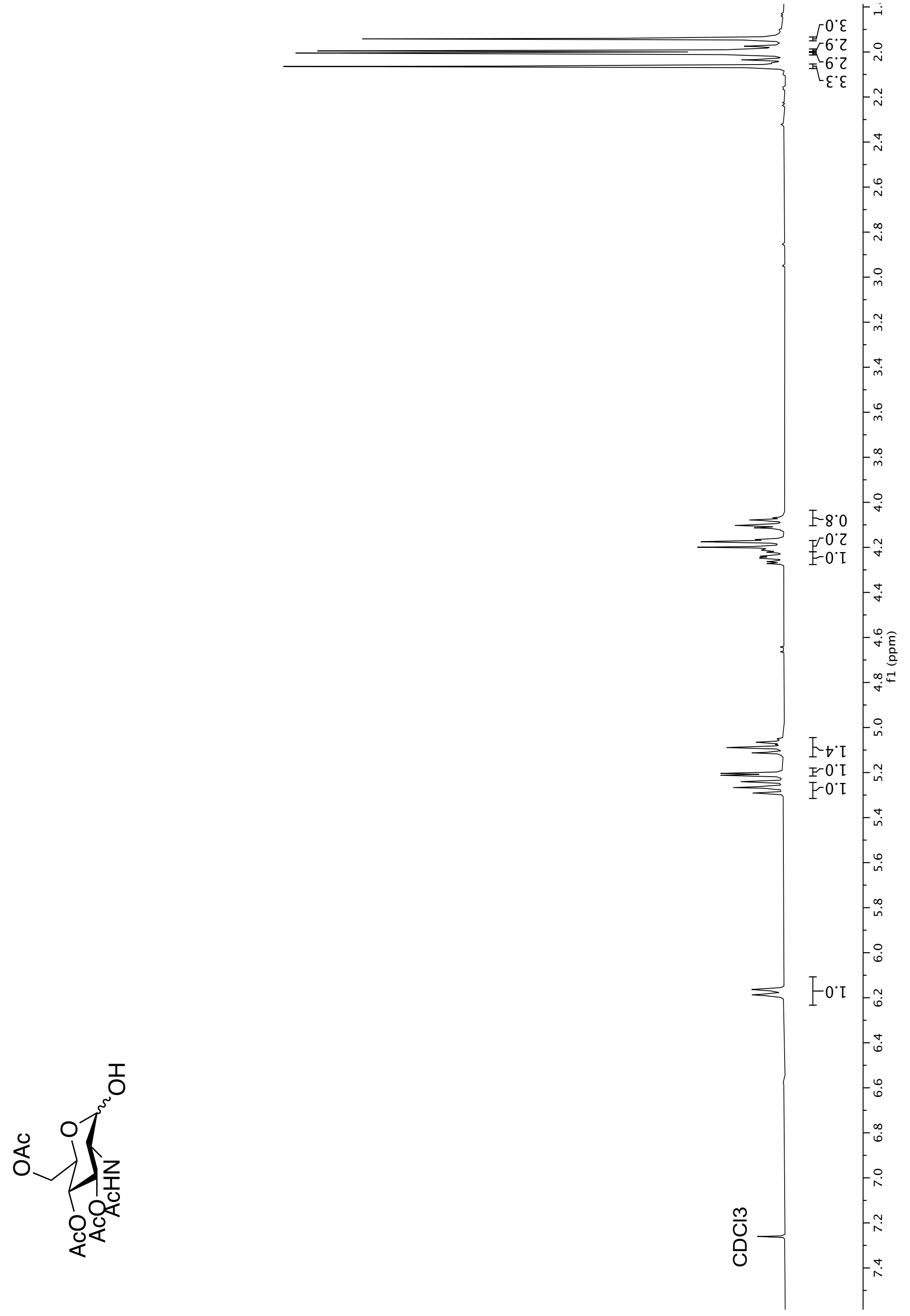

**$^{13}$C NMR 2-Acetamido-3, 4, 6-tri-*O*-acetyl-2-deoxy-D-glucopyranose (S1)**

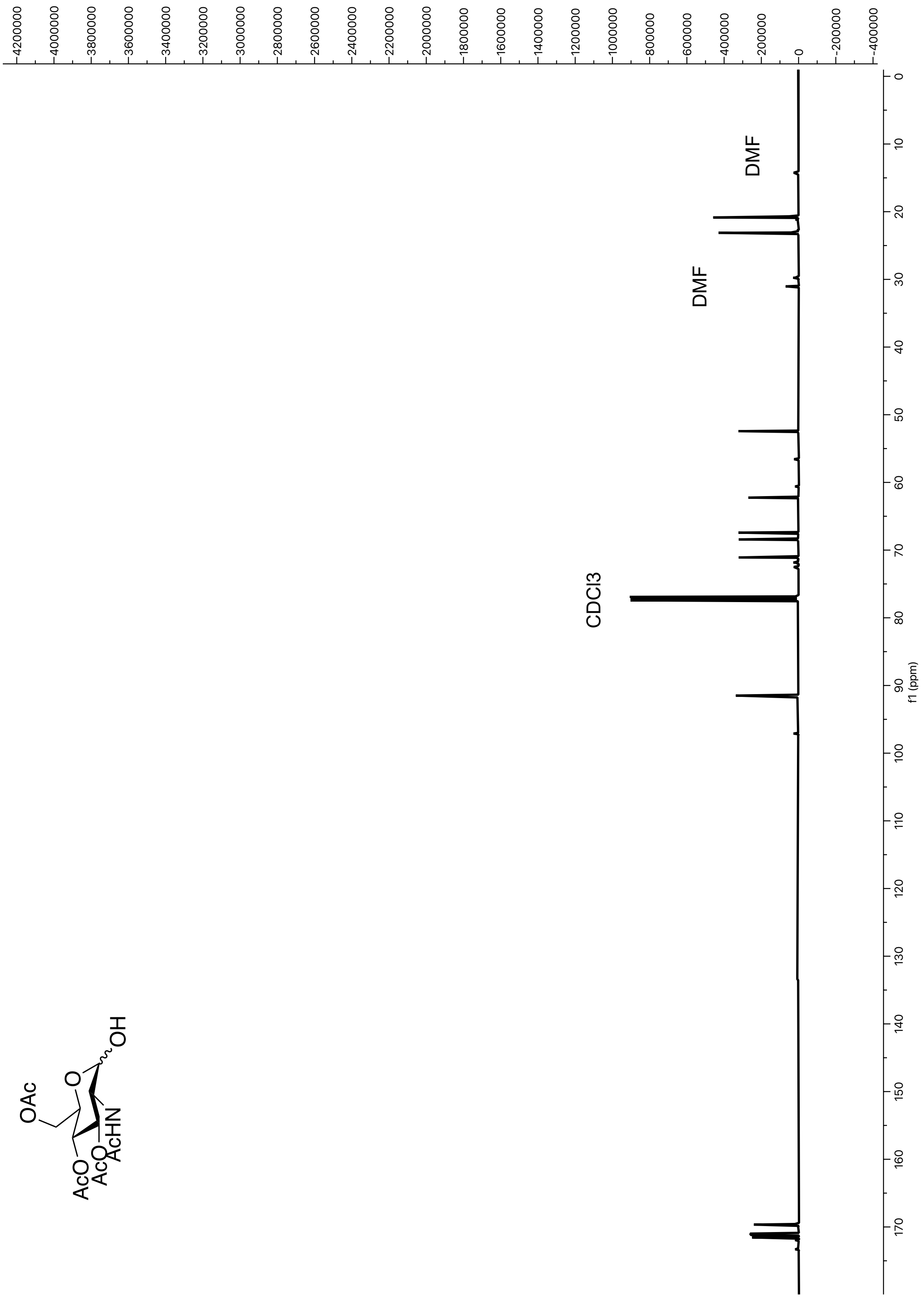

**$^{1}$H NMR 2-Acetamido-3,4,6-tri-*O*-acetyl-2-deoxy-α-D-glucopyranose 1-dibenzylphosphate (S2)**

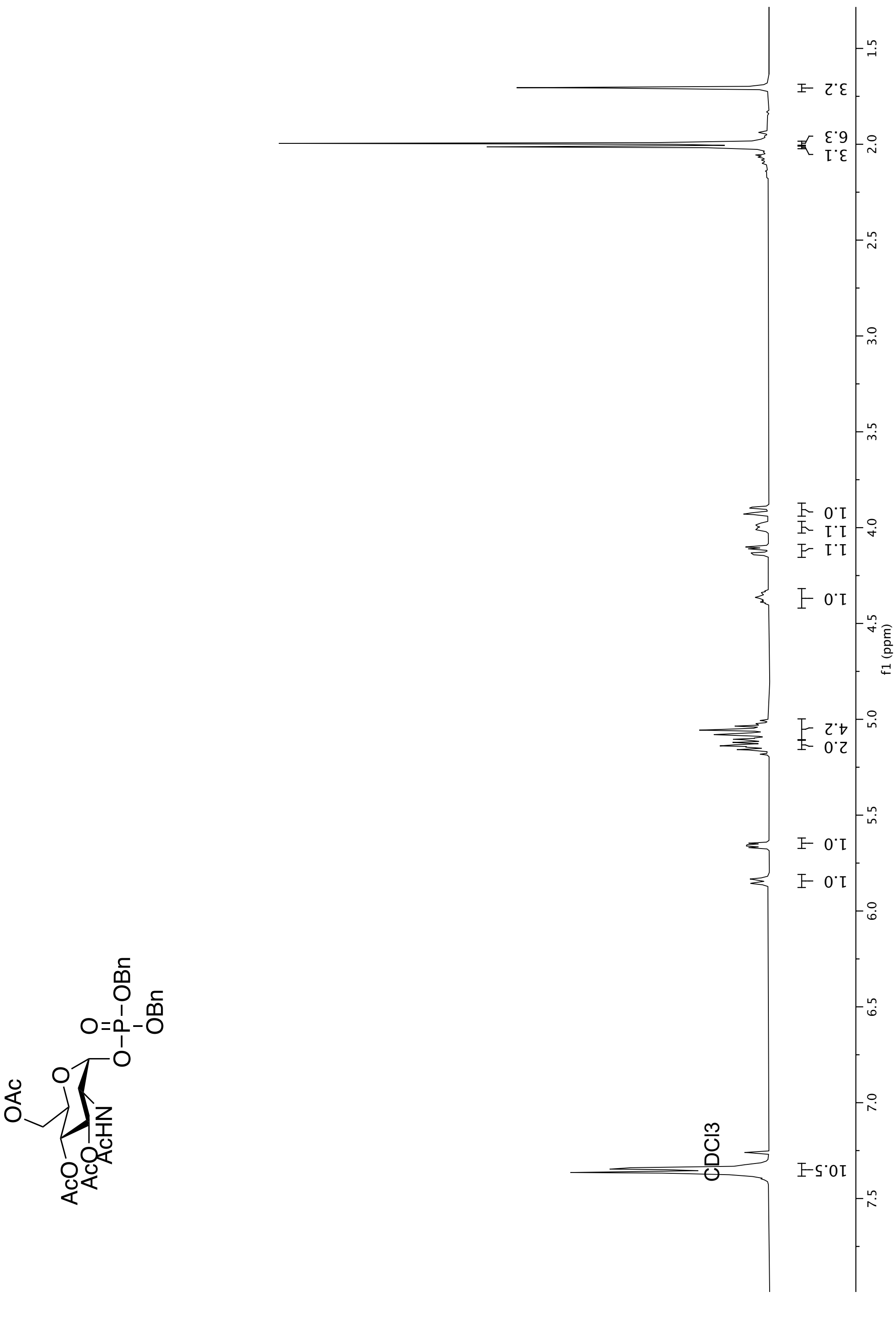

**$^{13}$C NMR 2-Acetamido-3,4,6-tri-*O*-acetyl-2-deoxy-α-D-glucopyranose 1-dibenzylphosphate (S2)**

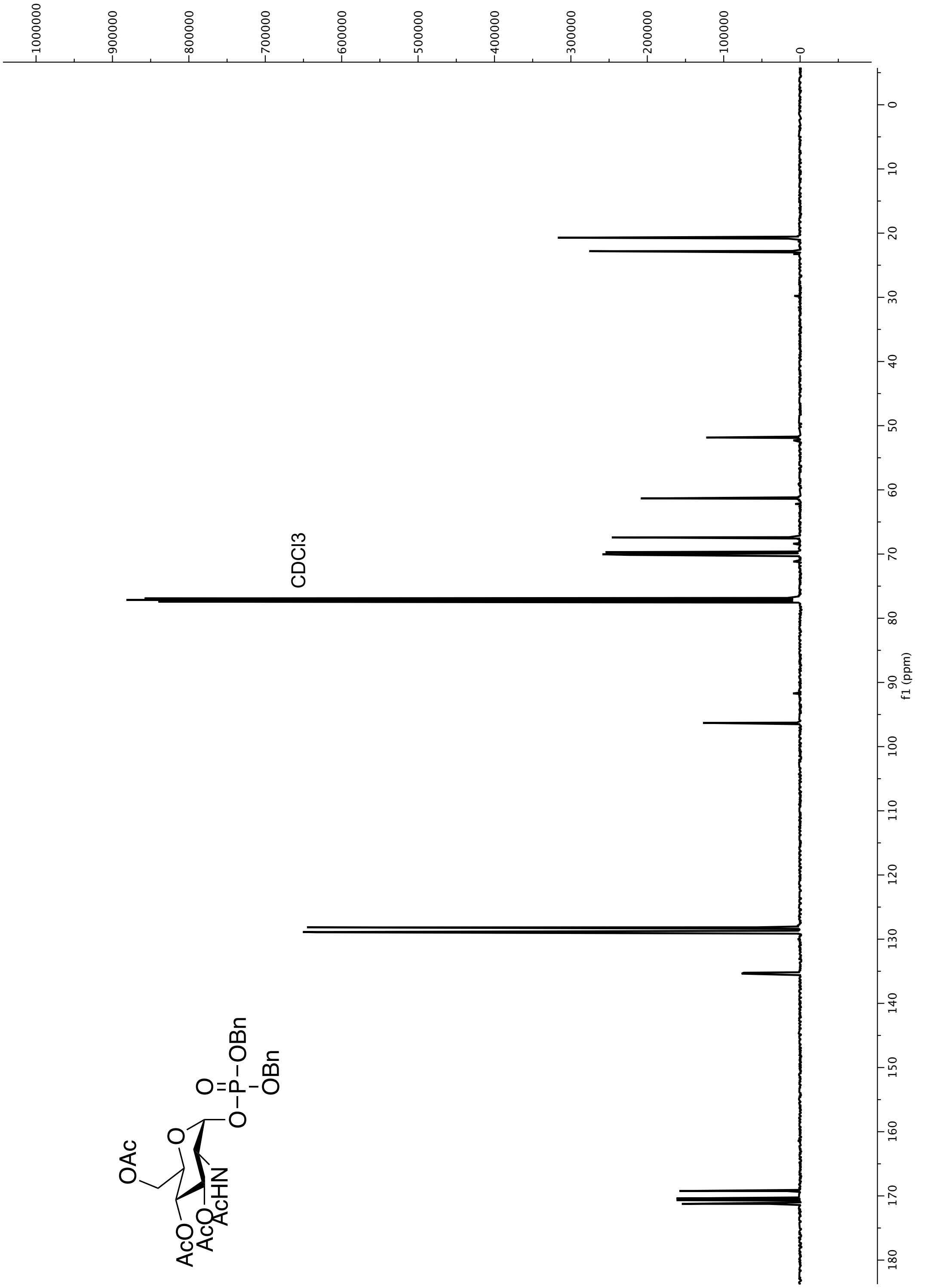

**$^{31}$P NMR 2-Acetamido-3,4,6-tri-*O*-acetyl-2-deoxy-α-D-glucopyranose 1-dibenzylphosphate (S2)**

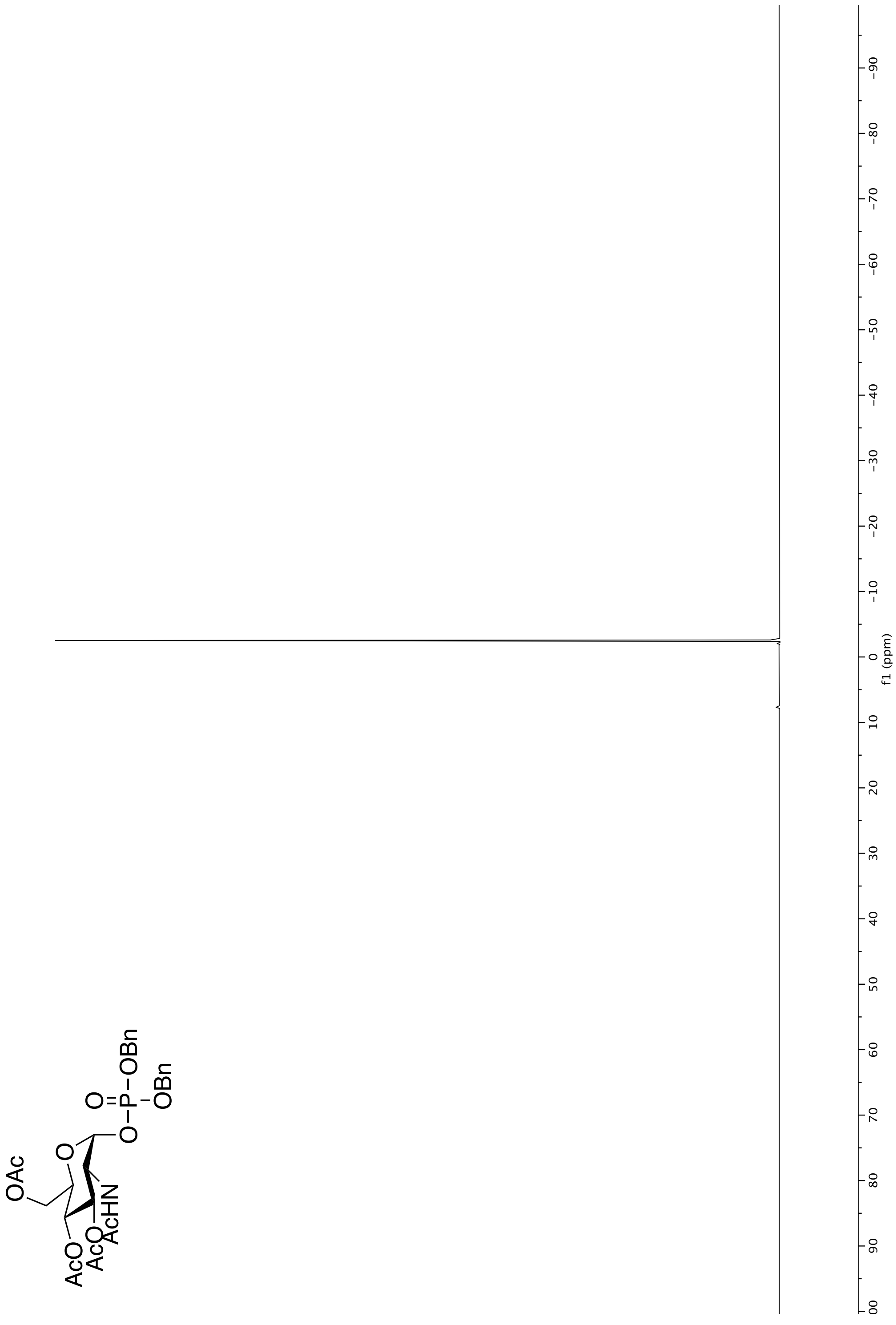

**$^{1}$H NMR 2-Acetamido-3,4,6-tri-*O*-acetyl-2-deoxy-α-D-glucopyranose 1-phosphate (S3)**

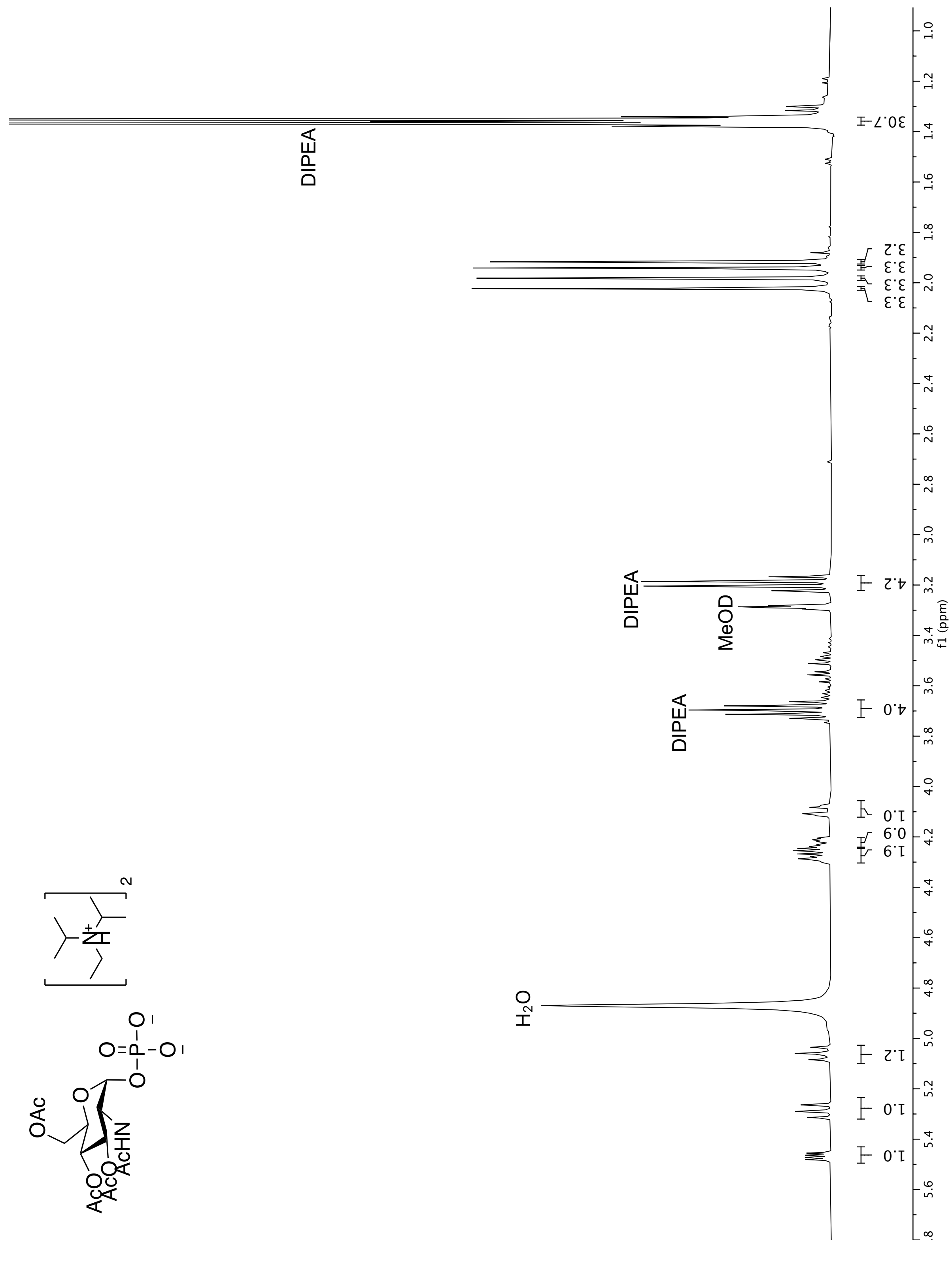

**$^{13}$C NMR 2-Acetamido-3,4,6-tri-*O*-acetyl-2-deoxy-α-D-glucopyranose 1-phosphate (S3)**

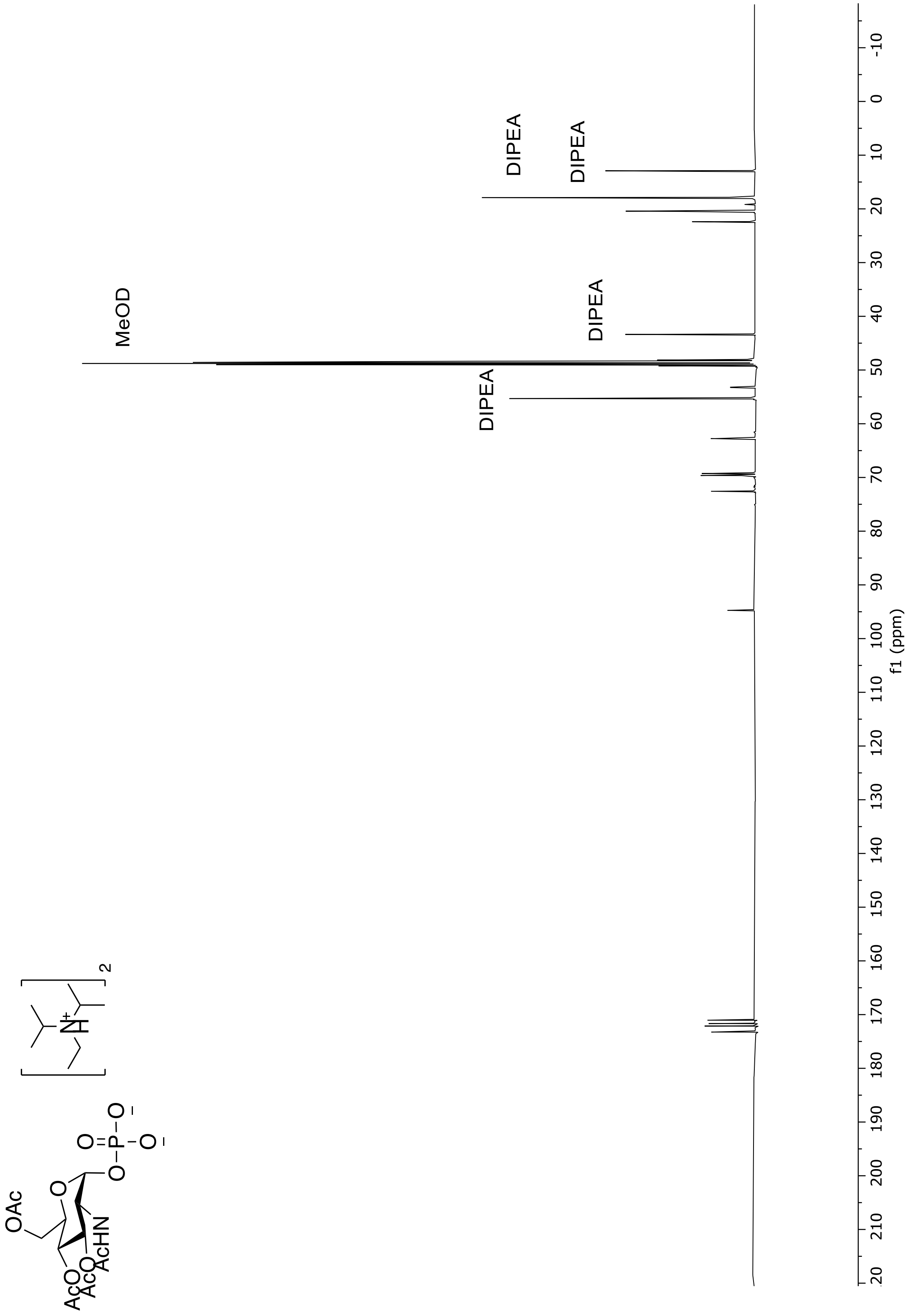

**$^{31}$P NMR 2-Acetamido-3,4,6-tri-*O*-acetyl-2-deoxy-α-D-glucopyranose 1-phosphate (S3)**

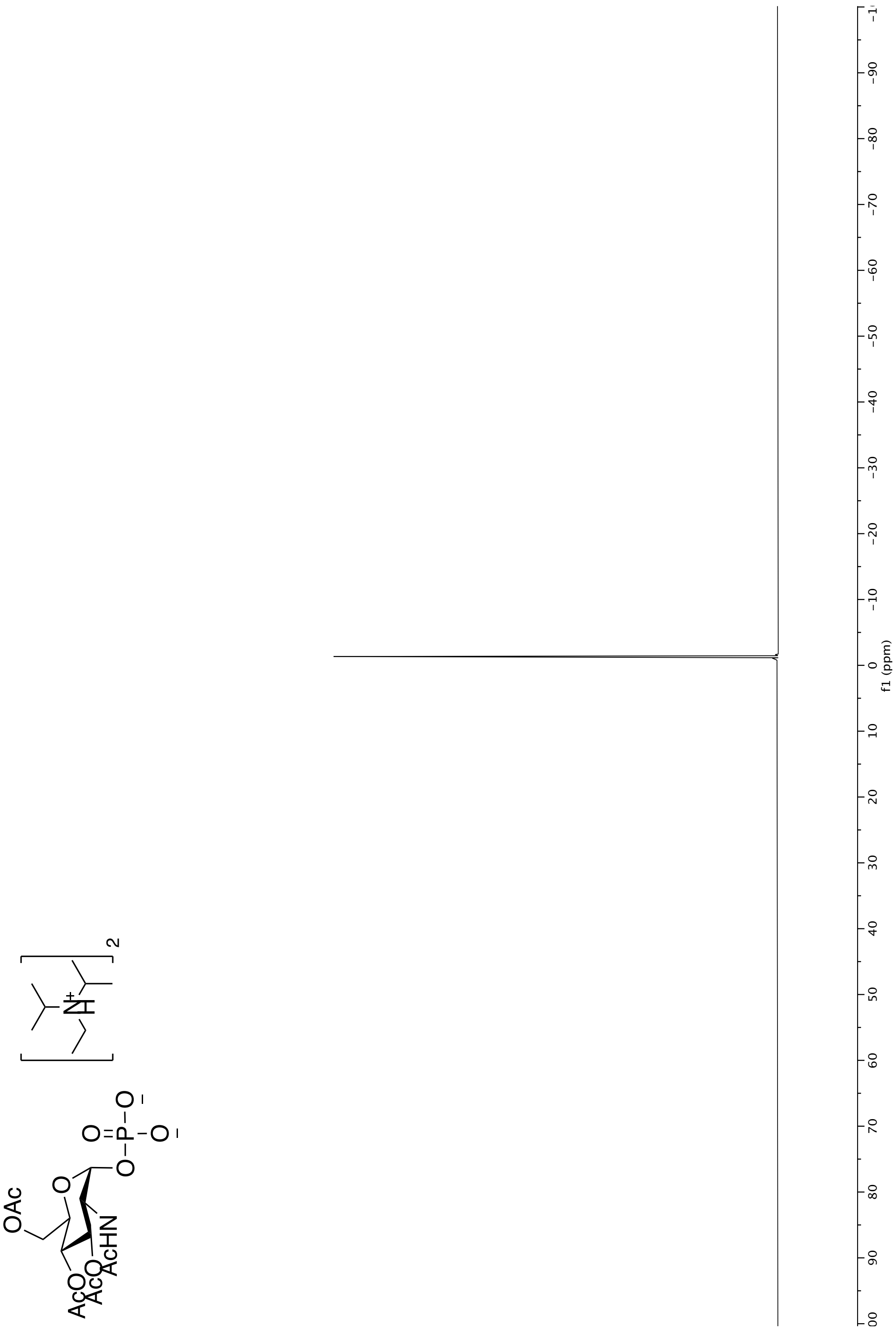

**$^{31}$P NMR 2-Acetamido-3,4,6-tri-*O*-acetyl-2-deoxy-α-D-glucopyranose 1-phosphoimidazolide (S4)**

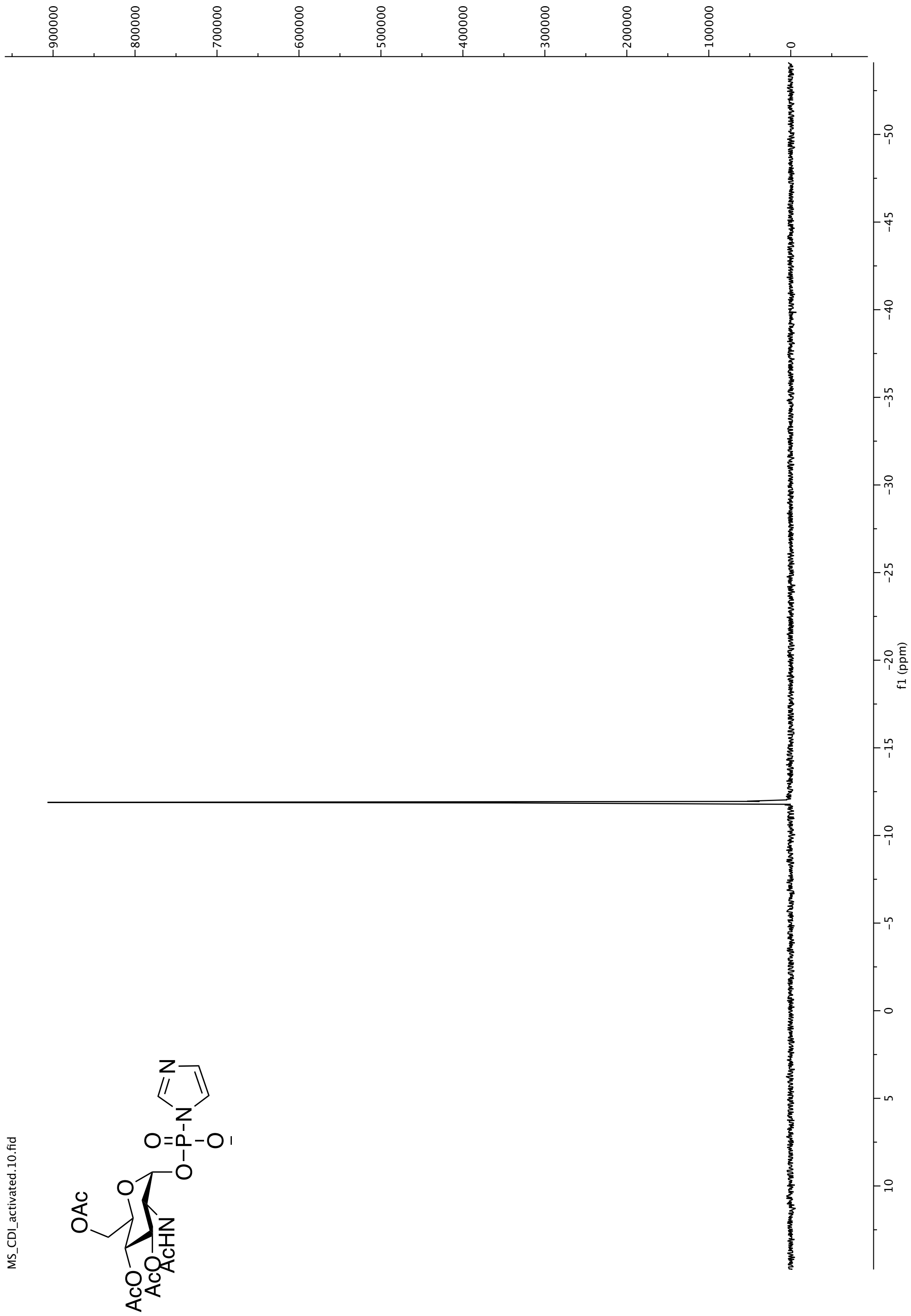

**$^1$H NMR P[1]-2-Acetamido-3,4,6-tri-*O*-acetyl-2-deoxy-α-D-glucopyranosyl P[2] -(2Z,6Z)-3,7,11-trimethyldodeca- 2,6,10-trien-1-yl diphosphate (S5)**

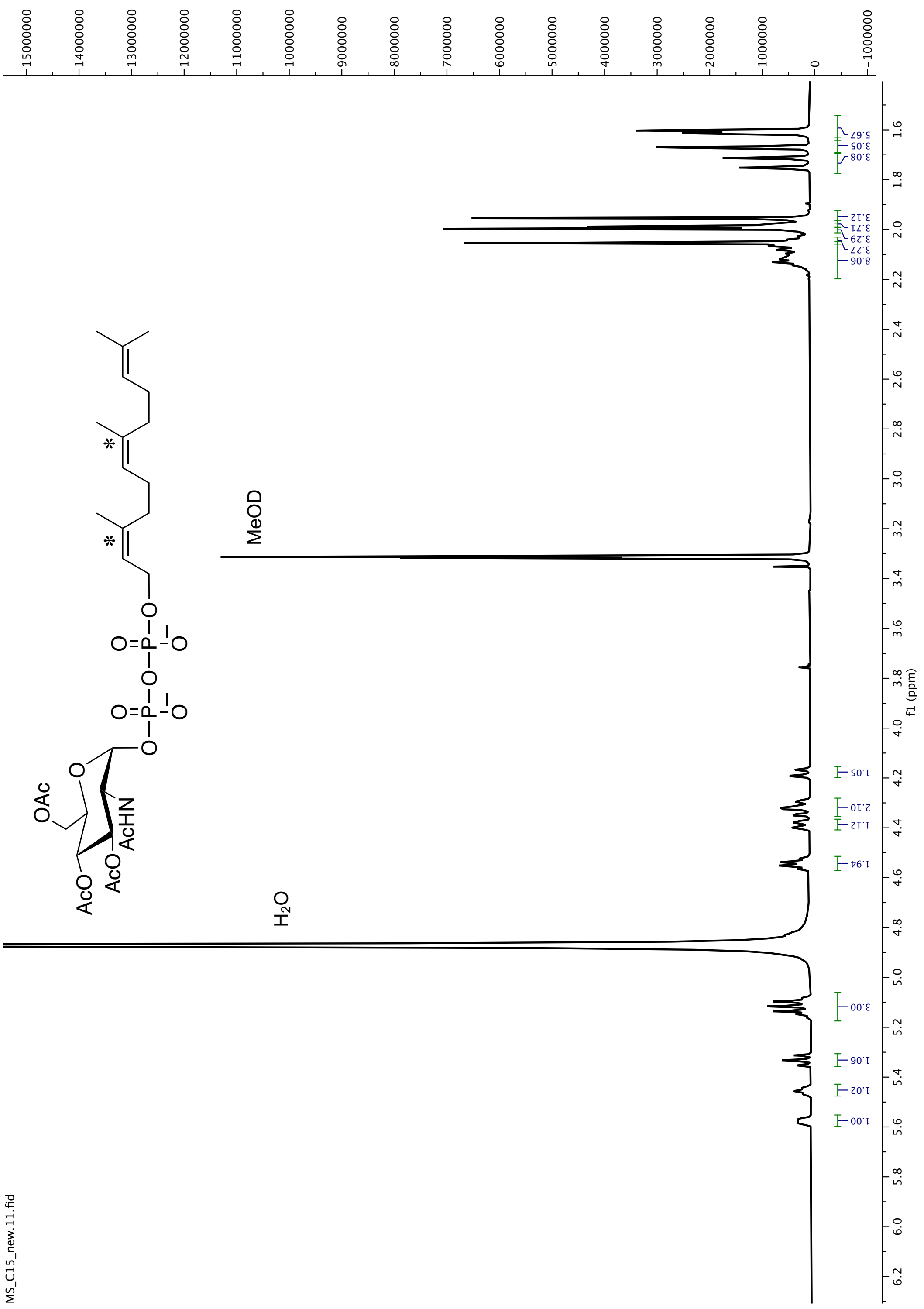

**$^{13}$C NMR P[1]-2-Acetamido-3,4,6-tri-*O*-acetyl-2-deoxy-α-D-glucopyranosyl P[2] -(2Z,6Z)-3,7,11-trimethyldodeca- 2,6,10-trien-1-yl diphosphate (S5)**

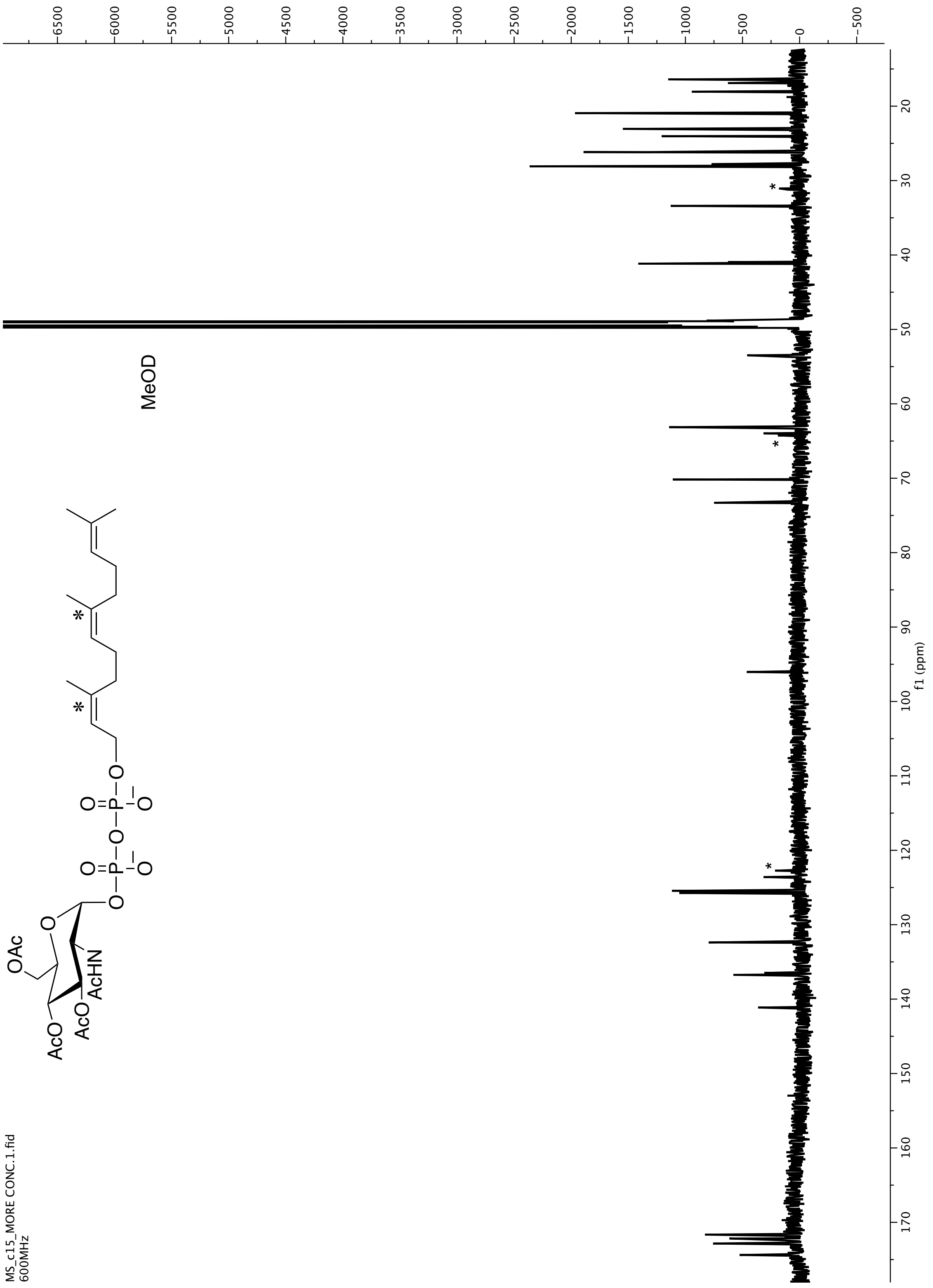

**$^{31}$P NMR P$^{1}$-2-Acetamido-3,4,6-tri-*O*-acetyl-2-deoxy-α-D-glucopyranosyl P$^{2}$ -(2Z,6Z)-3,7,11-trimethyldodeca- 2,6,10-trien-1-yl diphosphate (S5)**

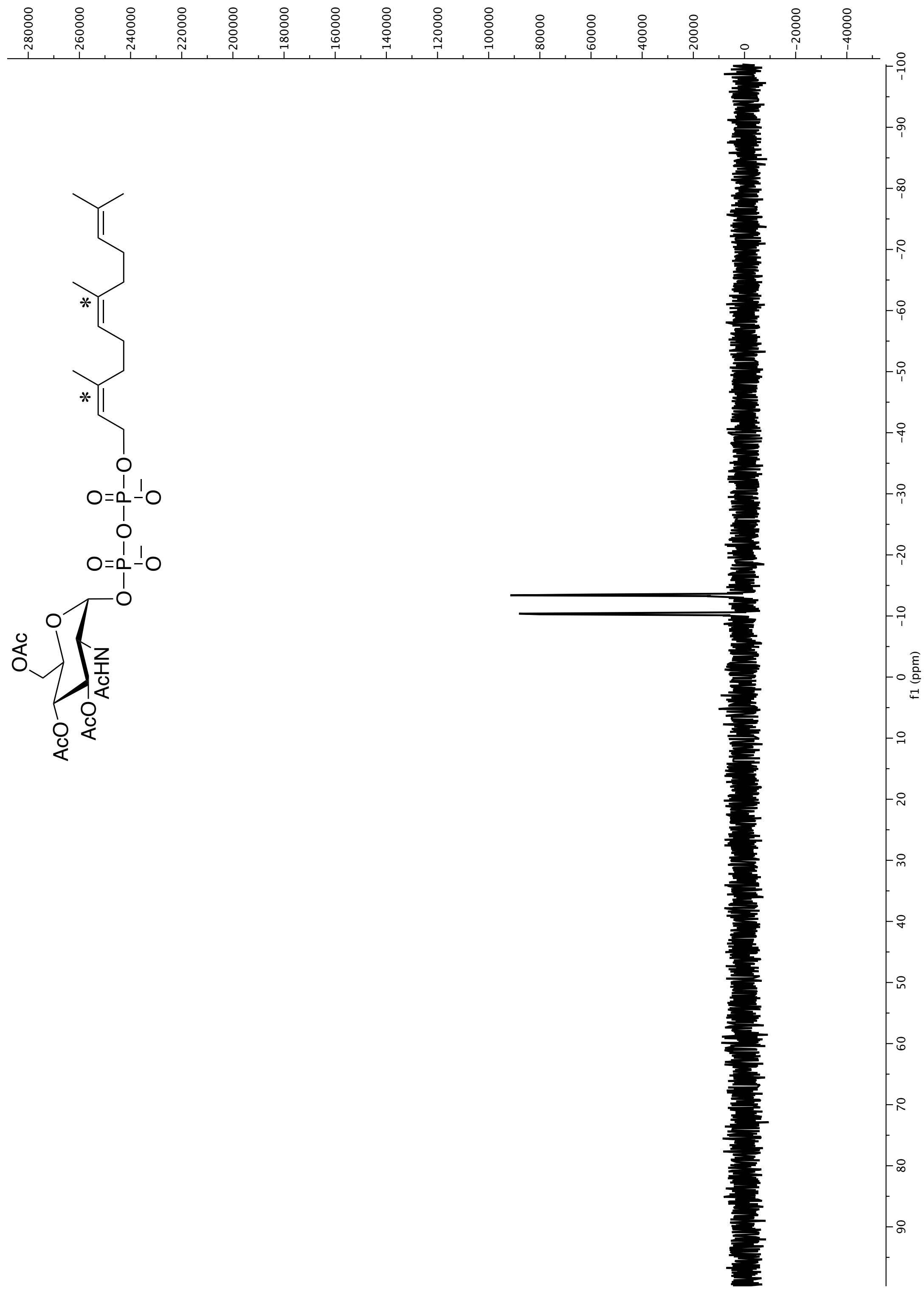

**$^{1}$H NMR $P^1$-2-Acetamido-3,4,6-tri-*O*-acetyl-2-deoxy-α-D-glucopyranosyl $P^2$-(2E,6E,10E)-3,7,11,15-tetramethyl hexadeca-2,6,10,14-tetraen-1-yl diphosphate (S6)**

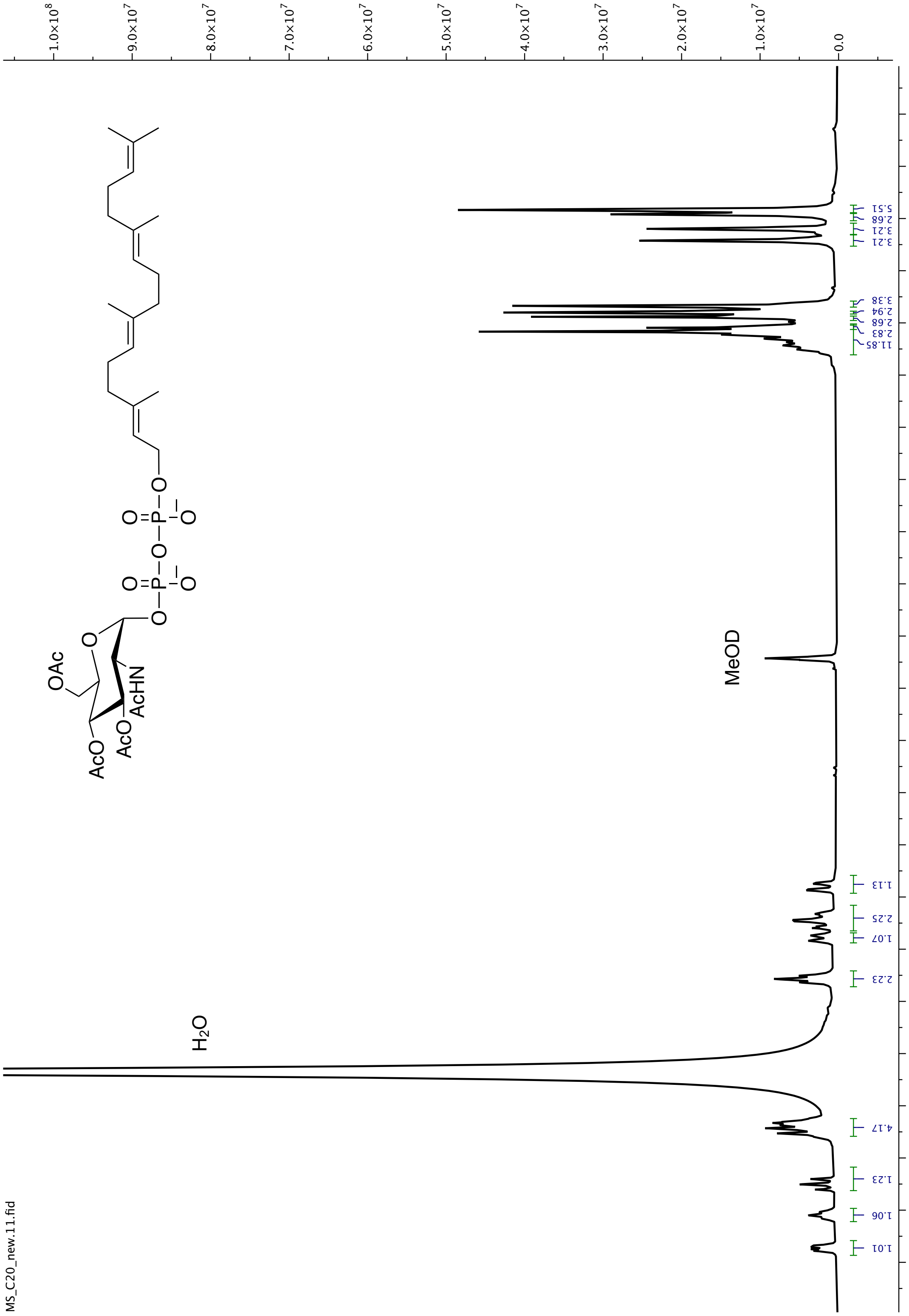

**$^{13}$C NMR P[1]-2-Acetamido-3,4,6-tri-*O*-acetyl-2-deoxy-α-D-glucopyranosyl P[2]-(2E,6E,10E)-3,7,11,15-tetramethyl hexadeca-2,6,10,14-tetraen-1-yl diphosphate (S6)**

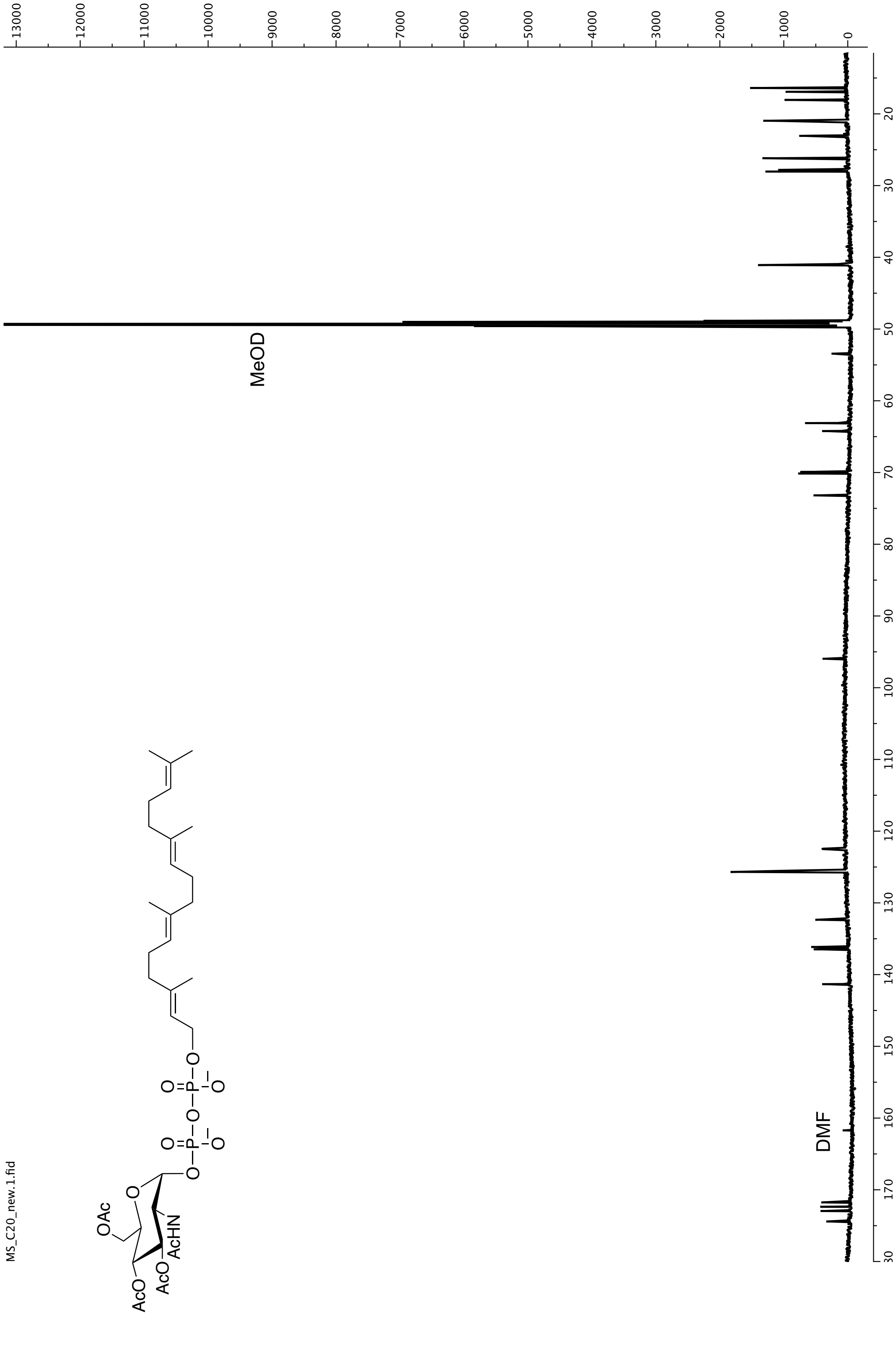

**$^{31}$P NMR P[1]-2-Acetamido-3,4,6-tri-*O*-acetyl-2-deoxy-α-D-glucopyranosyl P[2]-(2E,6E,10E)-3,7,11,15-tetramethyl hexadeca-2,6,10,14-tetraen-1-yl diphosphate (S6)**

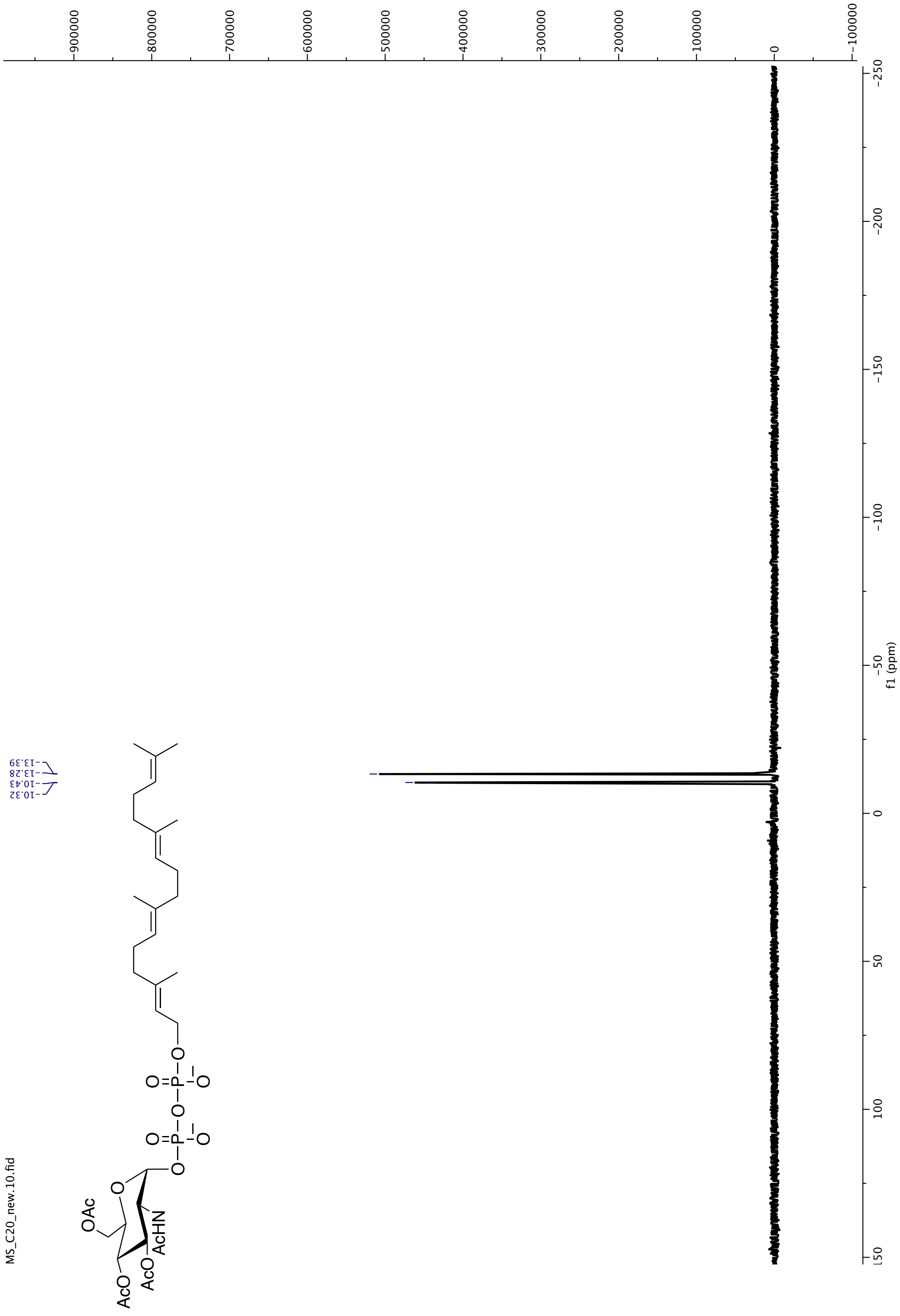

**$^1$H NMR P$^1$-2-Acetamido-3,4,6-tri-*O*-acetyl-2-deoxy-α-D-glucopyranosyl P$^2$ -(2Z,6Z,10Z,14Z,18E,22E)-3,7,11,15,19,23,27-heptamethyloctacosa-2,6,10,14,18,22,26-heptaen-1-yl diphosphate (S7)**

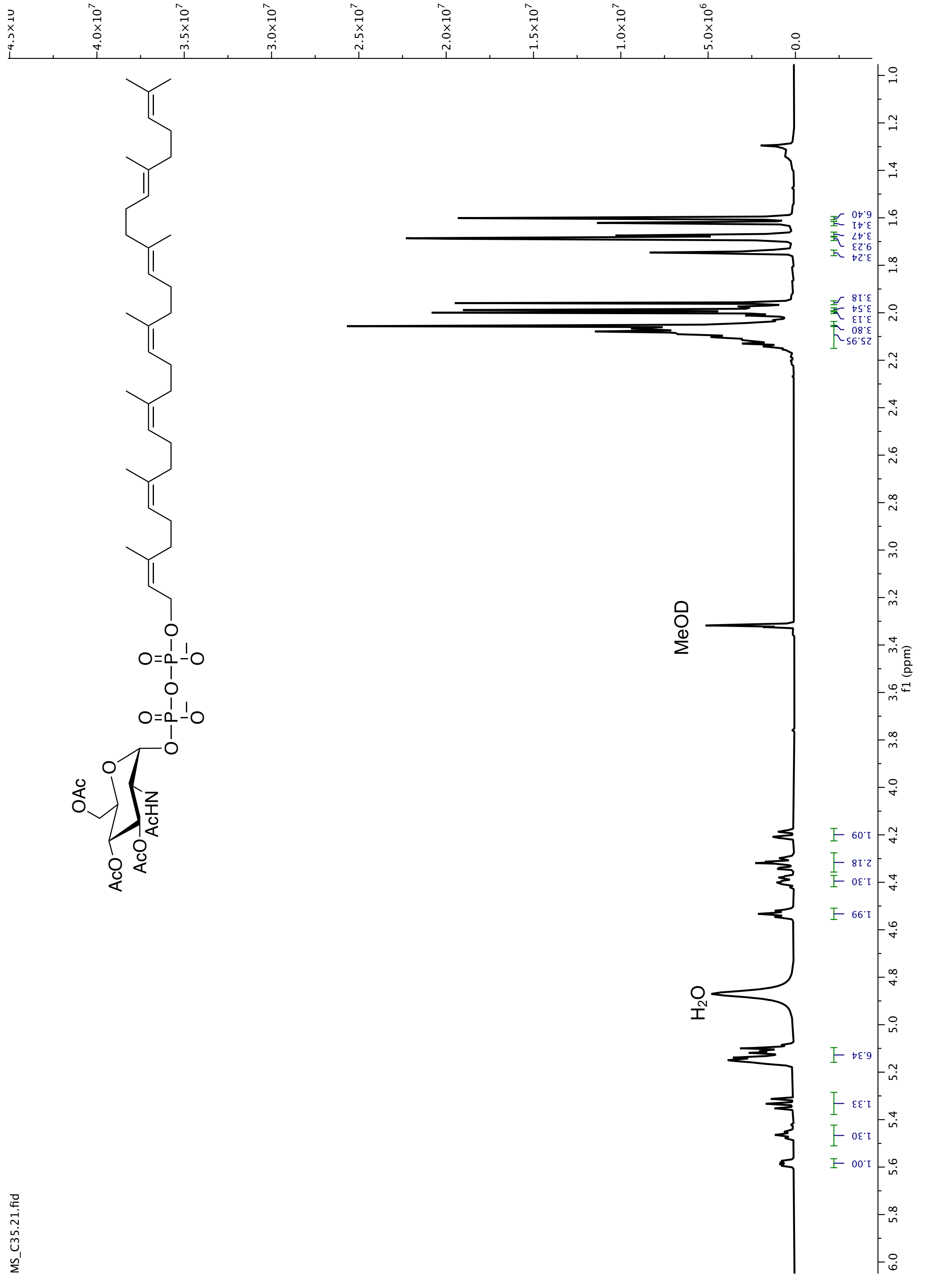

**$^{13}$C NMR P[1]-2-Acetamido-3,4,6-tri-*O*-acetyl-2-deoxy-α-D-glucopyranosyl P[2] -(2Z,6Z,10Z,14Z,18E,22E)-3,7,11,15,19,23,27-heptamethyloctacosa-2,6,10,14,18,22,26-heptaen-1-yl diphosphate (S7)**

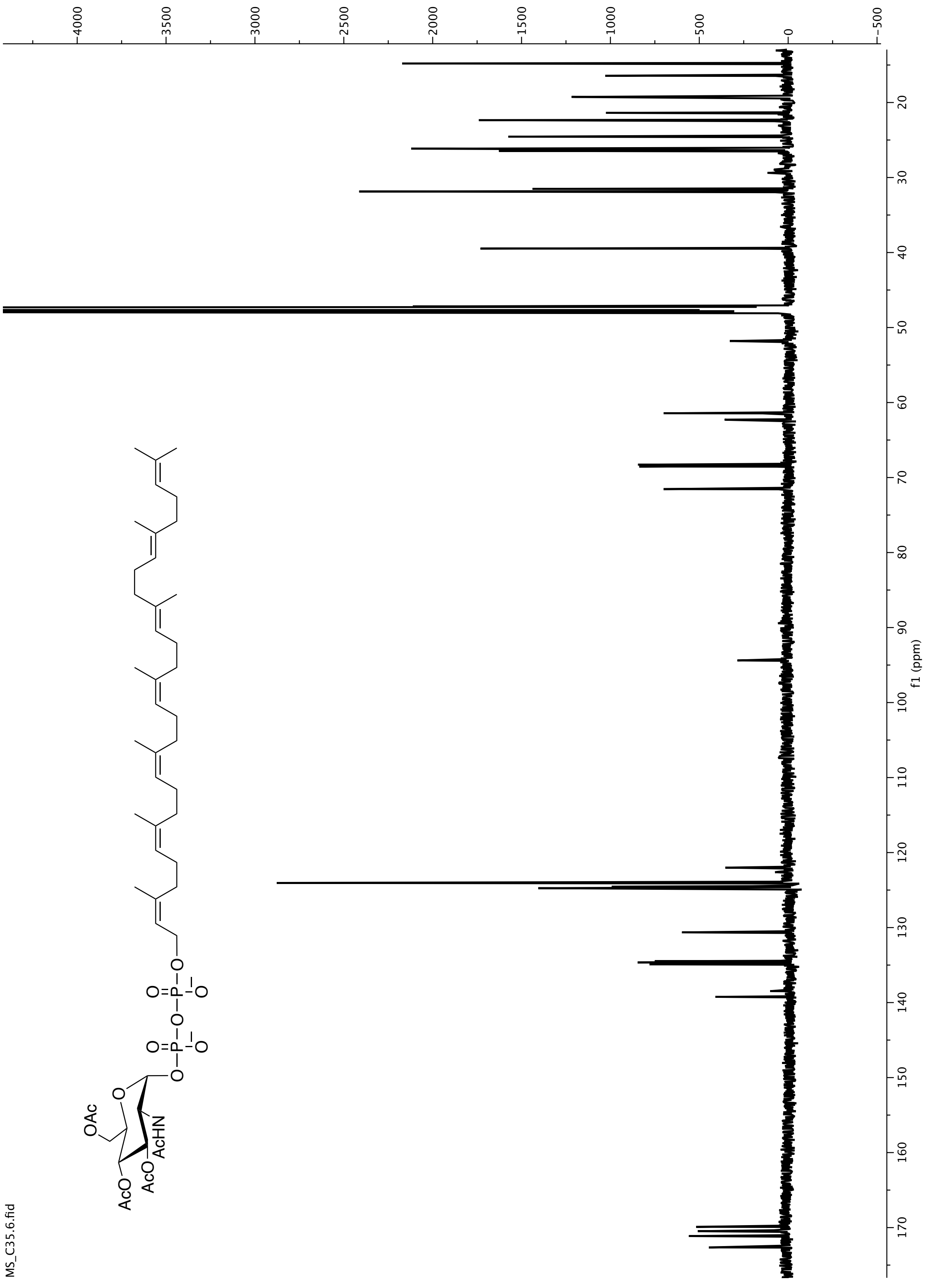

**$^{31}$P NMR P$^{1}$-2-Acetamido-3,4,6-tri-*O*-acetyl-2-deoxy-α-D-glucopyranosyl P$^{2}$ -(2Z,6Z,10Z,14Z,18E,22E)-3,7,11,15,19,23,27-heptamethyloctacosa-2,6,10,14,18,22,26-heptaen-1-yl diphosphate (S7)**

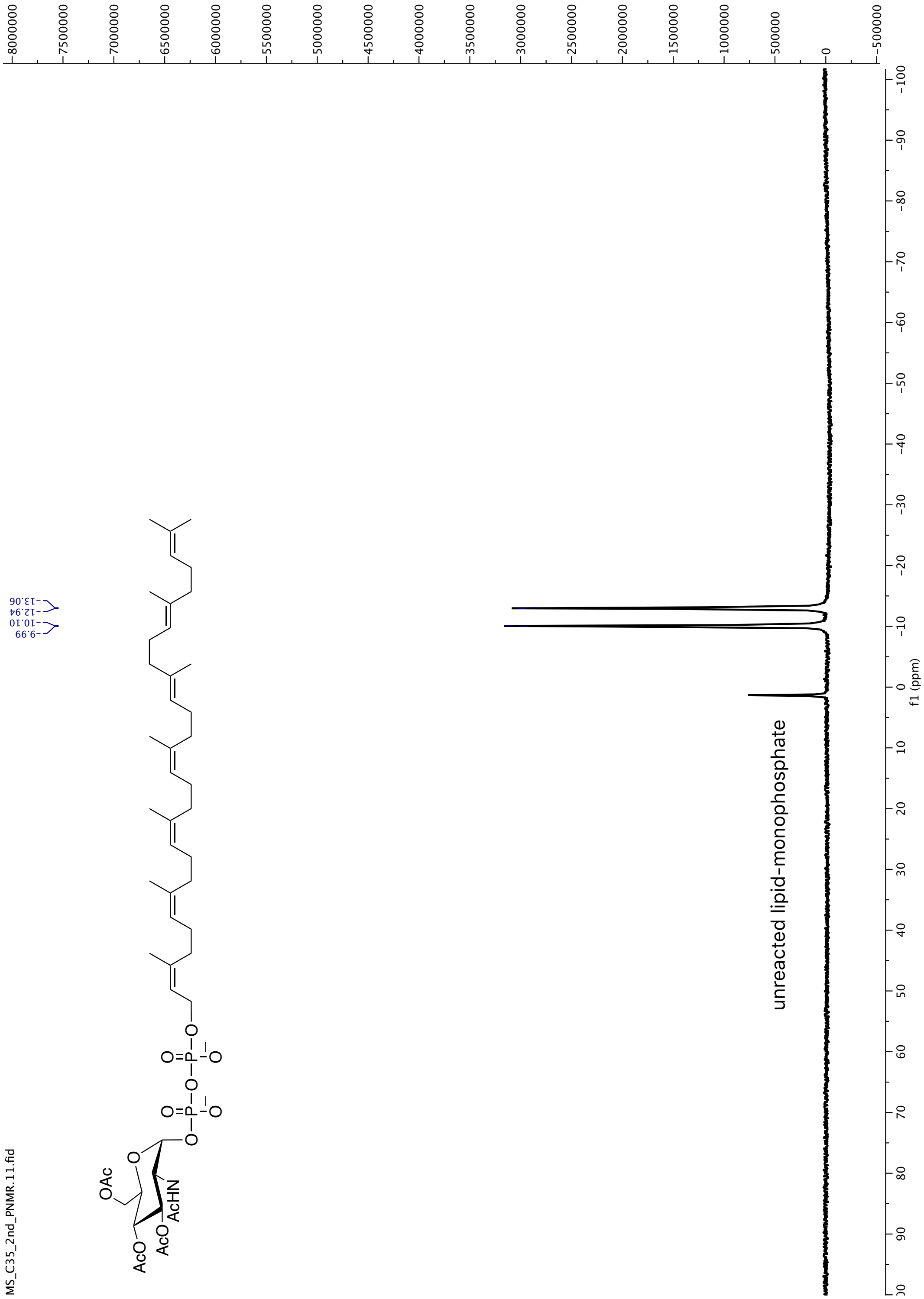

**$^{1}H$ NMR P[1]-2-Acetamido-3,4,6-tri-*O*-acetyl-2-deoxy-α-D-glucopyranosyl P[2] - (2Z,6Z,10Z,14Z,18Z,22Z,26Z,30E,34E,38E)-3,7, 11,15,19,23,27,31,35,39,43 - undecamethyltetratetraconta-2,6,10,14,18,22,26,30,34,38,42-undecaen-1-yl Diphosphate (S8)**

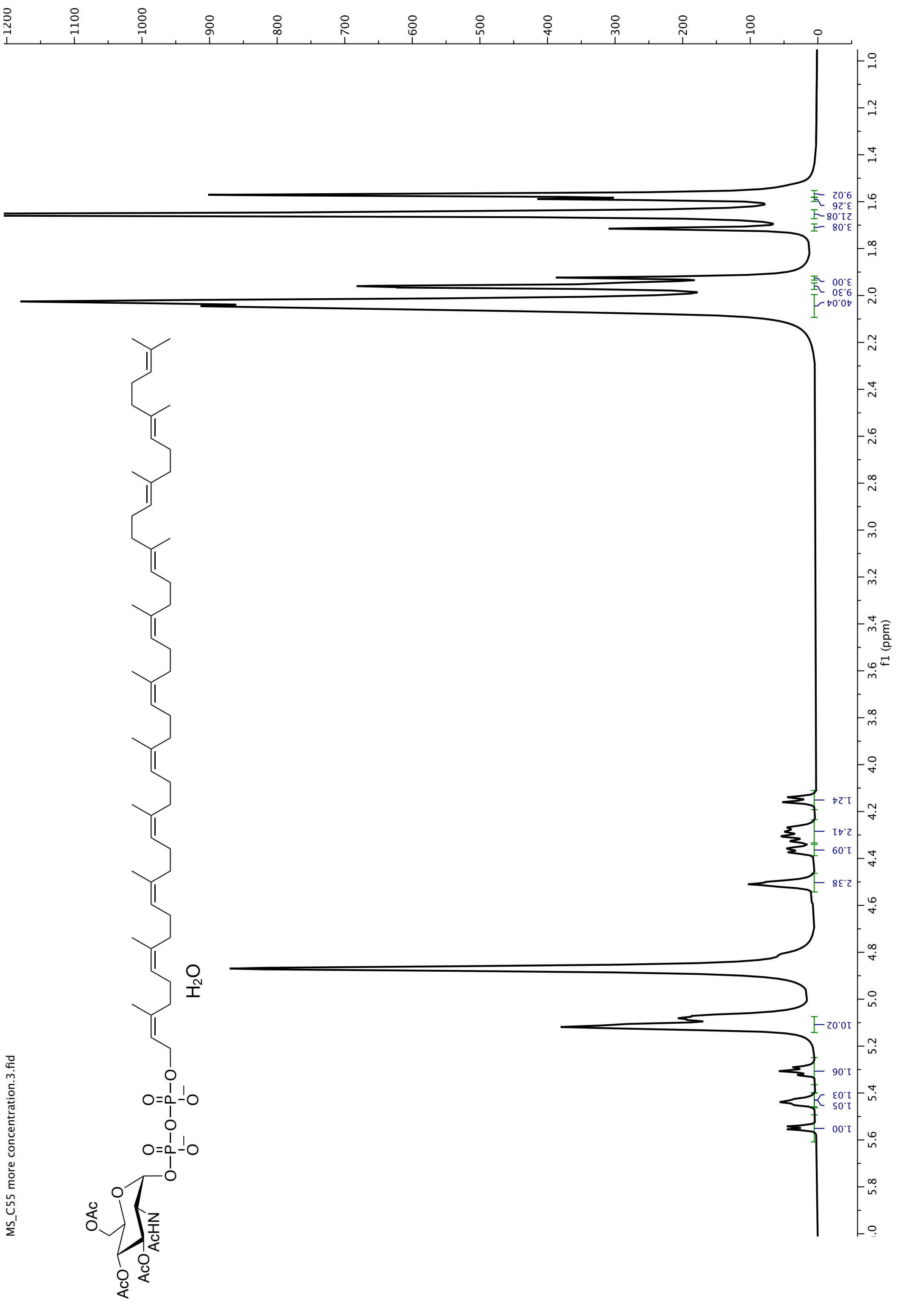

**$^{13}$C NMR P$^1$-2-Acetamido-3,4,6-tri-*O*-acetyl-2-deoxy-α-D-glucopyranosyl P$^2$ - (2Z,6Z,10Z,14Z,18Z,22Z,26Z,30E,34E,38E)-3,7, 11,15,19,23,27,31,35,39,43 - undecamethyltetratetraconta-2,6,10,14,18,22,26,30,34,38,42-undecaen-1-yl Diphosphate (S8)**

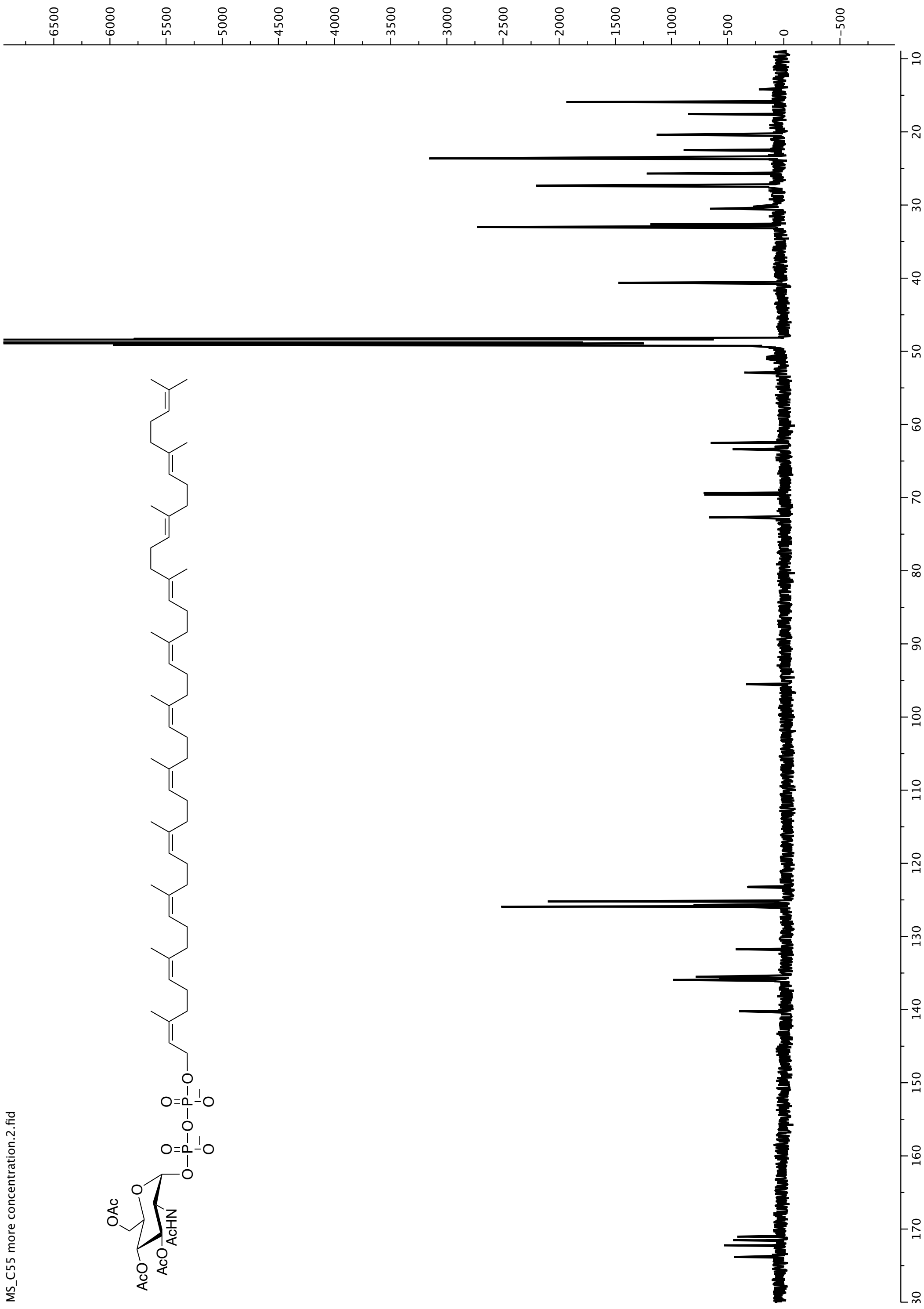

**$^{31}$P NMR P$^{1}$-2-Acetamido-3,4,6-tri-*O*-acetyl-2-deoxy-α-D-glucopyranosyl P$^{2}$ -(2Z,6Z,10Z,14Z,18Z,22Z,26Z,30E,34E,38E)-3,7, 11,15,19,23,27,31,35,39,43 -undecamethyltetratetraconta-2,6,10,14,18,22,26,30,34,38,42-undecaen-1-yl Diphosphate (S8)**

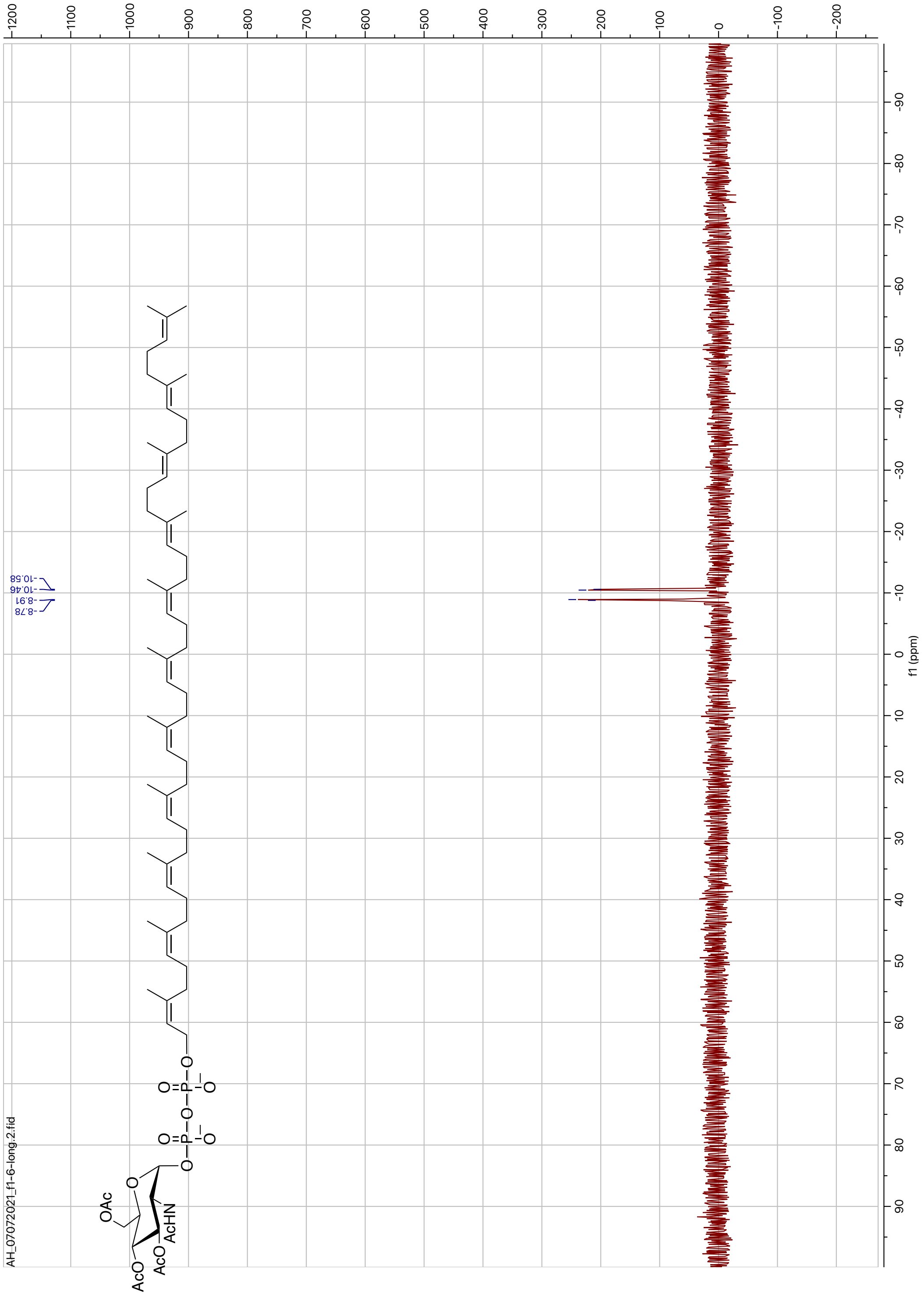

**$^{1}$H NMR 1,5-dideoxy-1,5-imino-L-rhamnitol (2)**

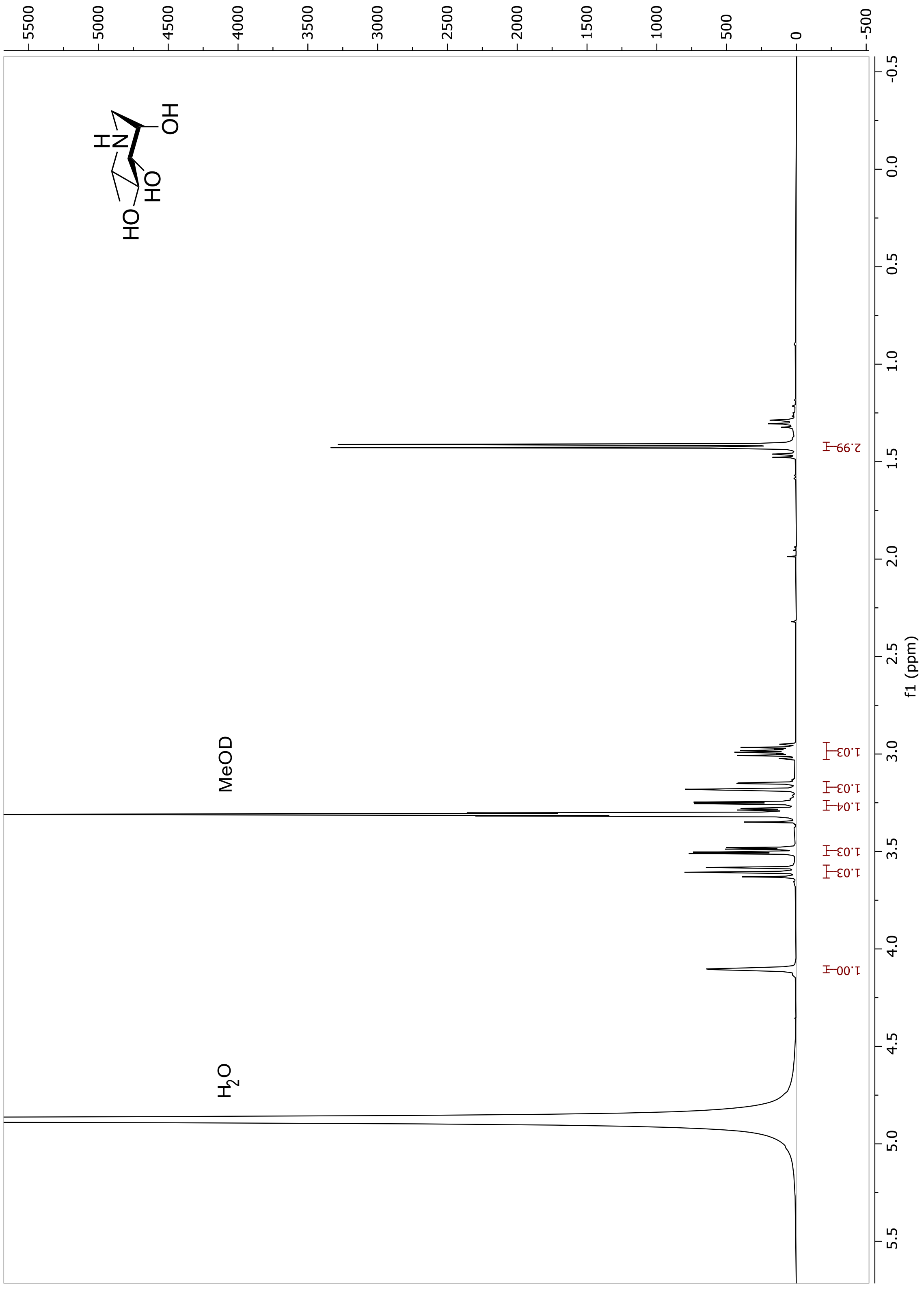

**$^{13}$C NMR 1,5-dideoxy-1,5-imino-L-rhamnitol (2)**

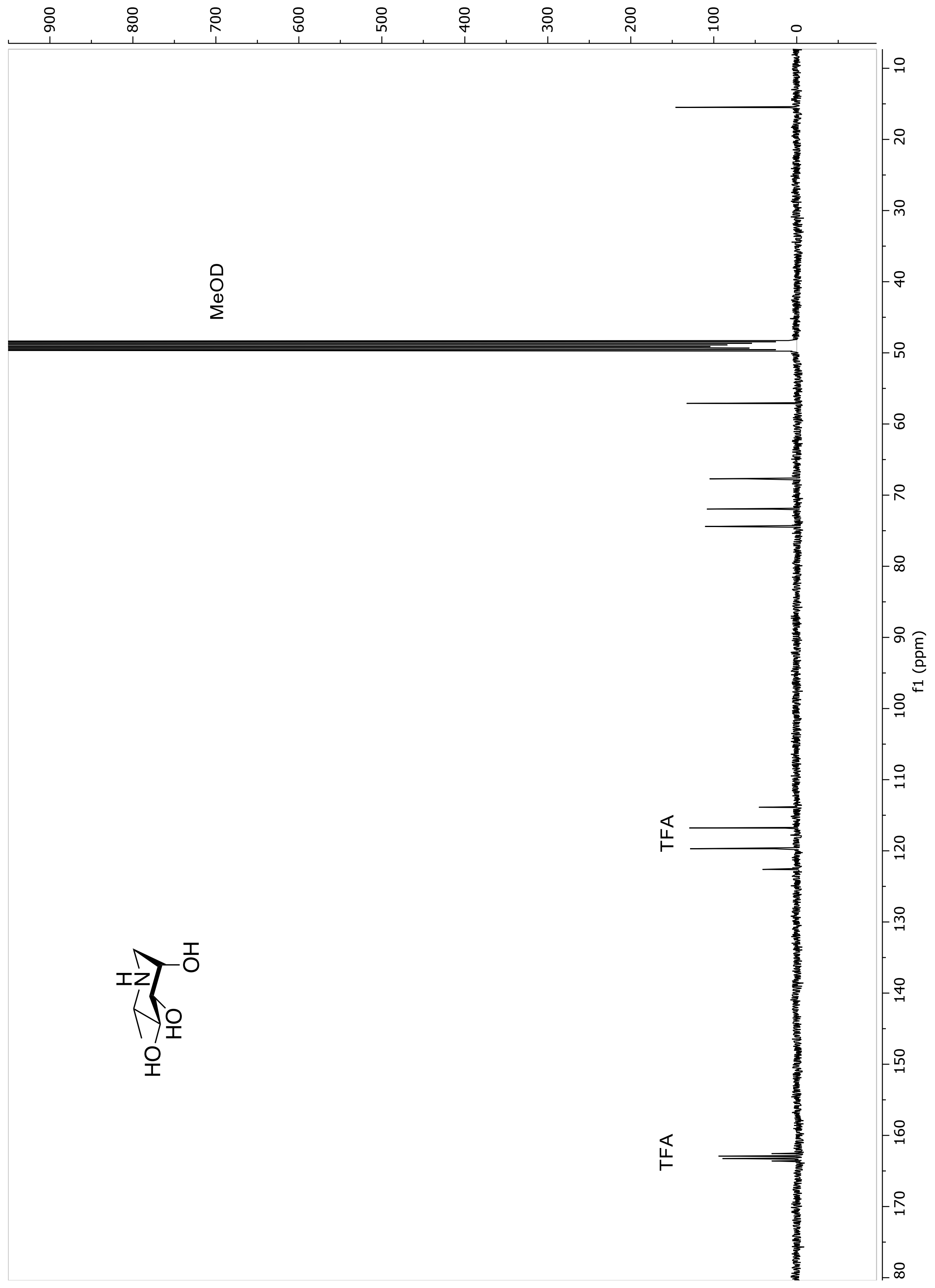

**References:**


1. Sievers, F.; Wilm, A.; Dineen, D.; Gibson, T. J.; Karplus, K.; Li, W.; Lopez, R.; McWilliam, H.; Remmert, M.; Soding, J.; Thompson, J. D.; Higgins, D. G., Fast, scalable generation of high-quality protein multiple sequence alignments using Clustal Omega. *Mol. Syst. Biol.* **2011,** *7*, 539-545.
2. Kilinc, M.; Jia, K.; Jernigan, R. L., Improved global protein homolog detection with major gains in function identification. *Proc. Natl. Acad. Sci. U.S.A.* **2023,** *120* (9), e2211823120.
3. Jumper, J.; Evans, R.; Pritzel, A.; Green, T.; Figurnov, M.; Ronneberger, O.; Tunyasuvunakool, K.; Bates, R.; Zidek, A.; Potapenko, A.; Bridgland, A.; Meyer, C.; Kohl, S. A. A.; Ballard, A. J.; Cowie, A.; Romera-Paredes, B.; Nikolov, S.; Jain, R.; Adler, J.; Back, T.; Petersen, S.; Reiman, D.; Clancy, E.; Zielinski, M.; Steinegger, M.; Pacholska, M.; Berghammer, T.; Bodenstein, S.; Silver, D.; Vinyals, O.; Senior, A. W.; Kavukcuoglu, K.; Kohli, P.; Hassabis, D., Highly accurate protein structure prediction with AlphaFold. *Nature* **2021,** *596* (7873), 583-589.
4. Varadi, M.; Anyango, S.; Deshpande, M.; Nair, S.; Natassia, C.; Yordanova, G.; Yuan, D.; Stroe, O.; Wood, G.; Laydon, A.; Zidek, A.; Green, T.; Tunyasuvunakool, K.; Petersen, S.; Jumper, J.; Clancy, E.; Green, R.; Vora, A.; Lutfi, M.; Figurnov, M.; Cowie, A.; Hobbs, N.; Kohli, P.; Kleywegt, G.; Birney, E.; Hassabis, D.; Velankar, S., AlphaFold Protein Structure Database: massively expanding the structural coverage of protein-sequence space with high-accuracy models. *Nucleic Acids Res.* **2022,** *50* (D1), D439-D444.
5. Chothia, C.; Lesk, A. M., The relation between the divergence of sequence and structure in proteins. *EMBO. J.* **1986,** *5* (4), 823-826.
6. Reva, B. A.; Finkelstein, A. V.; Skolnick, J., What is the probability of a chance prediction of a protein structure with an rmsd of 6 A? *Fold Des.* **1998,** *3* (2), 141-147.
7. Jorgenson, M. A.; Young, K. D., Interrupting Biosynthesis of O Antigen or the Lipopolysaccharide Core Produces Morphological Defects in Escherichia coli by Sequestering Undecaprenyl Phosphate. *J. Bacteriol.* **2016,** *198* (22), 3070-3079.
8. King, J. D.; Berry, S.; Clarke, B. R.; Morris, R. J.; Whitfield, C., Lipopolysaccharide O antigen size distribution is determined by a chain extension complex of variable stoichiometry in Escherichia coli O9a. *Proc. Natl. Acad. Sci. U.S.A.* **2014,** *111* (17), 6407-6412.
9. Whitfield, C.; Williams, D. M.; Kelly, S. D., Lipopolysaccharide O-antigens-bacterial glycans made to measure. *J. Biol. Chem.* **2020,** *295* (31), 10593-10609.
10. Raetz, C. R.; Whitfield, C., Lipopolysaccharide endotoxins. *Annu. Rev. Biochem.* **2002,** *71*, 635-700.
11. Lombard, V.; Golaconda Ramulu, H.; Drula, E.; Coutinho, P. M.; Henrissat, B., The carbohydrate-active enzymes database (CAZy) in 2013. *Nucleic Acids Res.* **2014,** *42* (Database issue), D490-D495.
12. Drula, E.; Garron, M. L.; Dogan, S.; Lombard, V.; Henrissat, B.; Terrapon, N., The carbohydrate-active enzyme database: functions and literature. *Nucleic Acids Res.* **2022,** *50* (D1), D571-D577.
13. Grzegorzewicz, A. E.; Ma, Y.; Jones, V.; Crick, D.; Liav, A.; McNeil, M. R., Development of a microtitre plate-based assay for lipid-linked glycosyltransferase products using the mycobacterial cell wall rhamnosyltransferase WbbL. *Microbiology* **2008,** *154* (Pt 12), 3724-3730.

14. Mills, J. A.; Motichka, K.; Jucker, M.; Wu, H. P.; Uhlik, B. C.; Stern, R. J.; Scherman, M. S.; Vissa, V. D.; Pan, F.; Kundu, M.; Ma, Y. F.; McNeil, M., Inactivation of the mycobacterial rhamnosyltransferase, which is needed for the formation of the arabinogalactan-peptidoglycan linker, leads to irreversible loss of viability. *J. Biol. Chem.* **2004,** *279* (42), 43540-43546.
15. Morona, R.; Mavris, M.; Fallarino, A.; Manning, P. A., Characterization of the *rfc* region of *Shigella flexneri*. *J. Bacteriol.* **1994,** *176* (3), 733-747.
16. Maczuga, N.; Tran, E. N. H.; Qin, J.; Morona, R., Interdependence of *Shigella flexneri* O Antigen and Enterobacterial Common Antigen Biosynthetic Pathways. *J. Bacteriol.* **2022,** *204* (4), e0054621.
17. Mi, H.; Muruganujan, A.; Ebert, D.; Huang, X.; Thomas, P. D., PANTHER version 14: more genomes, a new PANTHER GO-slim and improvements in enrichment analysis tools. *Nucleic Acids Res.* **2019,** *47* (D1), D419-D426.
18. Lairson, L. L.; Henrissat, B.; Davies, G. J.; Withers, S. G., Glycosyltransferases: structures, functions, and mechanisms. *Annu. Rev. Biochem.* **2008,** *77*, 521-555.
19. Hu, Y.; Walker, S., Remarkable structural similarities between diverse glycosyltransferases. *Chem. Biol.* **2002,** *9* (12), 1287-1296.
20. Breton, C.; Imberty, A., Structure/function studies of glycosyltransferases. *Curr. Opin. Struct. Biol.* **1999,** *9* (5), 563-571.
21. Zhang, Z.; Kochhar, S.; Grigorov, M., Exploring the sequence-structure protein landscape in the glycosyltransferase family. *Protein. Sci.* **2003,** *12* (10), 2291-2302.
22. Helbert, W.; Poulet, L.; Drouillard, S.; Mathieu, S.; Loiodice, M.; Couturier, M.; Lombard, V.; Terrapon, N.; Turchetto, J.; Vincentelli, R.; Henrissat, B., Discovery of novel carbohydrate-active enzymes through the rational exploration of the protein sequences space. *Proc. Natl. Acad. Sci. U.S.A.* **2019,** *116* (13), 6063-6068.
23. Wagstaff, B. A.; Rejzek, M.; Kuhaudomlarp, S.; Hill, L.; Mascia, I.; Nepogodiev, S. A.; Dorfmueller, H. C.; Field, R. A., Discovery of an RmlC/D fusion protein in the microalga Prymnesium parvum and its implications for NDP-β-L-rhamnose biosynthesis in microalgae. *J. Biol. Chem.* **2019,** *294* (23), 9172-9185.
24. Kelley, L. A.; Mezulis, S.; Yates, C. M.; Wass, M. N.; Sternberg, M. J., The Phyre2 web portal for protein modeling, prediction and analysis. *Nat. Protoc.* **2015,** *10* (6), 845-858.
25. Schrödinger, L., The PyMOL Molecular Graphics System, Version 2.0. **2022**.
26. Wagstaff, B. A.; Zorzoli, A.; Dorfmueller, H. C., NDP-rhamnose biosynthesis and rhamnosyltransferases: building diverse glycoconjugates in nature. *Biochem. J.* **2021,** *478* (4), 685-701.
27. Bryksin, A. V.; Matsumura, I., Overlap extension PCR cloning: a simple and reliable way to create recombinant plasmids. *Biotechniques* **2010,** *48* (6), 463-465.
28. van Soolingen, D.; Hermans, P. W.; de Haas, P. E.; Soll, D. R.; van Embden, J. D., Occurrence and stability of insertion sequences in *Mycobacterium tuberculosis* complex strains: evaluation of an insertion sequence-dependent DNA polymorphism as a tool in the epidemiology of tuberculosis. *J. Clin. Microbiol.* **1991,** *29* (11), 2578-2586.
29. Muona, M.; Aranko, A. S.; Iwai, H., Segmental isotopic labelling of a multidomain protein by protein ligation by protein trans-splicing. *Chembiochem.* **2008,** *9* (18), 2958-2961.
30. Westphal, O., Jann K., Bacterial lipopolysaccharides extraction with phenol-water and further applications of the procedure. *Methods in Carbohydr. Chem.* **1965,** *5*, 83-91.
31. Tsai, C. M.; Frasch, C. E., A sensitive silver stain for detecting lipopolysaccharides in polyacrylamide gels. *Anal. Biochem.* **1982,** *119* (1), 115-119.

32. Zheng, M., Zheng, M., Kim H., Lupoli T., Feedback inhibition of bacterial nucleotidylransferases by rare nucleotide L-sugars restricts substrate promiscuity. *J Am Chem Soc.* **2023**. DOI: 10.1021/jacs.3c02319.
33. Zheng, M.; Zheng, M.; Lupoli, T. J., Expanding the Substrate Scope of a Bacterial Nucleotidyltransferase via Allosteric Mutations. *ACS Infect. Dis.* **2022,** *8* (10), 2035-2044.
34. Li, L.; Woodward, R. L.; Han, W.; Qu, J.; Song, J.; Ma, C.; Wang, P. G., Chemoenzymatic synthesis of the bacterial polysaccharide repeating unit undecaprenyl pyrophosphate and its analogs. *Nat. Protoc.* **2016,** *11* (7), 1280-1298.
35. Bosco, M.; Massarweh, A.; Iatmanen-Harbi, S.; Bouhss, A.; Chantret, I.; Busca, P.; Moore, S. E.; Gravier-Pelletier, C., Synthesis and biological evaluation of chemical tools for the study of Dolichol Linked Oligosaccharide Diphosphatase (DLODP). *Eur. J. Med. Chem.* **2017,** *125*, 952-964.
36. Danilov, L. L.; Druzhinina, T. N.; Kalinchuk, N. A.; Maltsev, S. D.; Shibaev, V. N., Polyprenyl phosphates: synthesis and structure-activity relationship for a biosynthetic system of Salmonella anatum O-specific polysaccharide. *Chem. Phys. Lipids* **1989,** *51* (3-4), 191-203.
37. Liu, F.; Vijayakrishnan, B.; Faridmoayer, A.; Taylor, T. A.; Parsons, T. B.; Bernardes, G. J.; Kowarik, M.; Davis, B. G., Rationally designed short polyisoprenol-linked PglB substrates for engineered polypeptide and protein N-glycosylation. *J. Am. Chem. Soc.* **2014,** *136* (2), 566-569.
38. Dharuman, S.; Wang, Y.; Crich, D., Alternative synthesis and antibacterial evaluation of 1,5-dideoxy-1,5-imino-L-rhamnitol. *Carbohydr. Res.* **2016,** *419*, 29-32.
